# Regio‐ and Enantioconvergent Hydroallylation of Acrylates Enabled by γ‐Silyl‐Substituted Allyl Acetates

**DOI:** 10.1002/anie.202425256

**Published:** 2025-04-21

**Authors:** Hirotsugu Suzuki, Ryoichi Nishikawa, Yuki Sato, Kaisei Sekino, Takanori Matsuda

**Affiliations:** ^1^ Tenure‐Track Program for Innovative Research University of Fukui 3‐9‐1 Bunkyo, Fukui‐shi Fukui 910‐8507 Japan; ^2^ Department of Applied Chemistry Tokyo University of Science 1–3 Kagurazaka Shinjuku‐ku Tokyo 162‐8601 Japan

**Keywords:** Copper, Enantioconvergent catalysis, Hydroallylation, Palladium, Vinylsilanes

## Abstract

Transition‐metal‐catalyzed asymmetric allylic substitution provides an efficient route to chiral organic molecules featuring an allyl moiety, key intermediates in the synthesis of biologically active compounds. However, the use of unsymmetrical 1,3‐disubstituted allyl electrophiles has been severely constrained by the challenges of achieving both regio‐ and stereoselectivity simultaneously. Herein, we present γ‐silyl‐substituted allyl acetates as highly effective electrophiles for a regio‐ and enantioconvergent hydroallylation, enabling the construction of vicinal stereogenic centers. This method delivers allylated products in 44%–93% yield with 79:21 to >95:5 dr and 88% to >99% ee. Additionally, the silyl group in the products can be readily converted into other functional groups, such as acyl and aryl groups, enhancing their synthetic utility.

## Graphical abstract

Regio‐ and enantioconvergent hydroallylation of acylates under Cu/Pd synergistic catalysis is reported. The incorporation of a silyl group into allyl acetates precisely controls regio‐ and enantioselectivity through steric hindrance. Subsequent transformation of the silyl group in the product enables the regioselective synthesis of allylated products bearing two distinct yet structurally similar aryl groups.

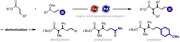

Transition‐metal‐catalyzed asymmetric allylic substitution has emerged as a powerful and efficient approach for enantioselective carbon–carbon (C─C) bond formation in a single step.^[^ ^1^, ^2^, ^3^, ^4^, ^5^, ^6^ ^]^ Despite the broad utility of this strategy, the use of unsymmetrical 1,3‐disubstituted allyl electrophiles remains significantly unexplored compared to their monosubstituted counterparts^[^ ^7^, ^8^, ^9^, ^10^, ^11^, ^12^ ^]^ or symmetrical analogues.^[^ ^13^, ^14^, ^15^, ^16^, ^17^, ^18^, ^19^, ^20^, ^21^, ^22^ ^]^ This limited exploration stems from the inherent challenges of achieving precise control over both regio‐ and stereoselectivity. Regioselectivity issues arise from the dual electrophilic sites in unsymmetrical 1,3‐disubstituted allyl acetates, where catalysts often struggle to differentiate substituents with similar steric or electronic properties,^[^ ^23^, ^24^ ^]^ thereby restricting the variety of usable substrates. Achieving stereoselectivity is equally challenging, as many reactions proceed via net retention (double inversion)^[^ ^25^, ^26^, ^27^, ^28^, ^29^, ^30^ ^]^ or kinetic resolution,^[^ ^31^, ^32^, ^33^, ^34^ ^]^ necessitating the use of chiral starting materials or resulting in the loss of half of the starting material. Enantioconvergent allylic substitution provides a promising solution but has predominantly focused on the formation of a single stereogenic center.^[^ ^35^, ^36^, ^37^, ^38^ ^]^ While Liao and Zhang et al. have demonstrated a stereodivergent approach capable of generating two stereogenic centers (Scheme 1a),^[^ ^39^ ^]^ their method is restricted to combinations of small alkyl and aryl groups in allyl acetate. Consequently, despite recent progress, the enantioconvergent construction of vicinal stereogenic centers employing versatile unsymmetrical 1,3‐disubstituted allyl electrophiles remains in its infancy.

**Scheme 1:**
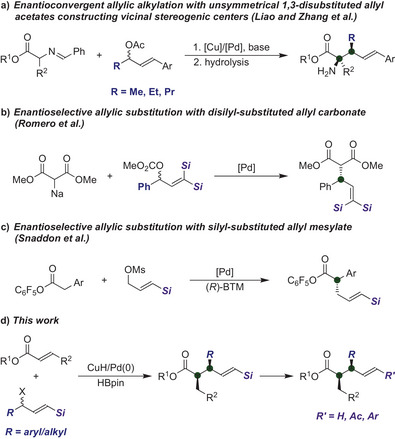
Enantioconvergent allylic substitutions using unsymmetrical 1,3‐disubstituted allyl electrophiles.

To address these challenges, we hypothesized that introducing a vinylsilane group to allyl electrophiles could expand the scope of asymmetric allylic substitutions while enabling dual control over regio‐ and stereoselectivity. Specifically, the silyl group, which is easily removable^[^ ^40^, ^41^, ^42^ ^]^ and highly transformable,^[^ ^43^, ^44^, ^45^, ^46^ ^]^ offers significant synthetic flexibility. Additionally, its steric bulk allows for effective differentiation of electrophilic sites and enantiotopic faces. The allylic substation with a silyl‐substituted allyl electrophile was initially reported by Hirao et al.,^[^ ^47^, ^48^, ^49^ ^]^ and subsequent studies have applied these electrophiles to enantioselective reactions.^[^ ^50^, ^51^ ^]^ Romero and coworkers have demonstrated the enantioselective allylic substitution of dimethyl malonate (Scheme 1b).^[^ ^50^ ^]^ However, this transformation requires geminal disilyl groups on the allyl electrophile to facilitate the formation of a chiral π‐allyl palladium intermediate. Moreover, this approach constructed only a single chiral carbon center. Most recently, Snaddon et al. investigated the enantioselective allylic substitution of C1‐ammonium enolates with silyl‐substituted allyl mesylates, which results in the formation of a single chiral carbon center (Scheme 1c).^[^ ^51^ ^]^ Despite these advancements in enantioselective allylic substitution,^[^ ^50^, ^51^, ^52^, ^53^, ^54^ ^]^ the stereoselective construction of vicinal stereogenic centers using silyl‐substituted allyl electrophiles remains an unresolved challenge. To overcome this limitation, we report the development of a regio‐ and enantioconvergent hydroallylation^[^ ^55^, ^56^, ^57^, ^58^, ^59^, ^60^, ^61^, ^62^, ^63^, ^64^, ^65^, ^66^ ^]^ of acrylates employing racemic γ‐silyl‐substituted allyl acetates under Cu/Pd synergistic catalysis (Scheme 1d).^[^ ^67^, ^68^, ^69^, ^70^, ^71^, ^72^, ^73^, ^74^, ^75^ ^]^ The γ‐silyl‐substituted allyl acetates introduced here can accommodate both alkyl and aryl substituents at the α‐position. Moreover, this strategy enables the efficient formation of vicinal stereogenic centers with complete regioselectivity and excellent diastereo‐ and enantioselectivities, while demonstrating the synthetic versality of these products. Notably, the silyl group can be converted into an aryl group, facilitating the efficient synthesis of an allylated compound bearing two distinct but structurally similar aryl groups regioselectively.

Our investigation began with *tert*‐butyl acrylate (**1a**) and γ‐silyl allyl acetate **2a** as model substrates (Table 1). Using Pd(dba)_2_/XPhos (Pd‐**L1**) as the palladium catalyst in THF at 40 °C for 18 h, the reaction produced *tert*‐butyl 4‐pentenoate **3a** as the sole product in 88% yield. Furthermore, the stereoselectivities were well controlled, affording **3a** with 87:13 dr and 99% ee (entry 1). Encouraged by these promising results, we optimized bisphosphine ligands for the copper catalyst, focusing on both BINAP‐ and SEGPHOS‐type ligands (entries 2–6). BINAP‐type ligands generally provided higher enantioselectivities than SEGPHOS‐type ligands. Notably, DTBM‐SEGPHOS (Cu‐**L6**) failed to yield any product, likely due to steric hindrance from its bulky substituents (entry 6).^[^ ^55^, ^56^ ^]^ Among the tested ligands, (*R*)‐tol‐BINAP (Cu‐**L2**) delivered the best diastereo‐ and enantioselectivities (entry 2). Next, we evaluated biaryl phosphine ligands for the palladium catalyst (entries 7 and 8), identifying RuPhos (Pd‐**L3**) as the optimal ligand (entry 8). Switching the solvent to 1,2‐dimethoxyethane (DME) further enhanced both the yield and diastereoselectivity (entry 9). Finally, to confirm the necessity of synergistic catalysis, we performed control reactions omitting either the copper or palladium catalyst (entries 10 and 11). These experiments revealed that both catalysts are essential for the reaction to proceed. Importantly, no regioisomers, such as δ‐phenyl‐β‐silyl‐4‐pentenoate, were observed in any of the optimization reactions.

**Table 1:** Optimization of reaction conditions.^a)^

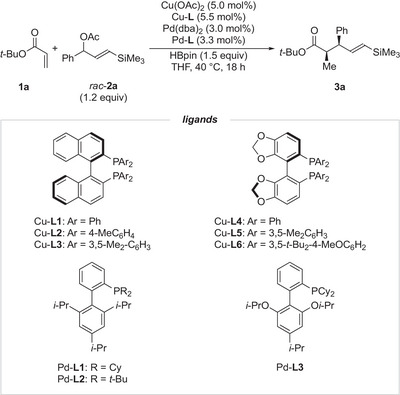					
Entry	Cu‐L	Pd‐L	Yield (%)^b)^	dr^c)^	ee (%)
1	Cu‐**L1**	Pd‐**L1**	88	87:13	99
2	Cu‐**L2**	Pd‐**L1**	86	89:11	99
3	Cu‐**L3**	Pd‐**L1**	75	86:14	99
4	Cu‐**L4**	Pd‐**L1**	83	87:13	94
5	Cu‐**L5**	Pd‐**L1**	74	84:16	98
6	Cu‐**L6**	Pd‐**L1**	Trace	–	–
7	Cu‐**L2**	Pd‐**L2**	76	88:12	95
8	Cu‐**L2**	Pd‐**L3**	87	89:11	99
9^d)^	Cu‐**L2**	Pd‐**L3**	93	90:10	99
10^d)^, ^e)^	–	Pd‐**L3**	0	–	–
11^d)^, ^f)^	Cu‐**L2**	–	0	–	–

^a)^ Reaction Conditions: **1a** (0.2 mmol), **2a** (0.24 mmol), Cu(OAc)_2_ (5.0 mol%), Cu‐**L** (5.5 mol%), Pd(dba)_2_ (3.0 mol%), Pd‐**L** (3.3 mol%), and HBpin (0.3 mmol) were reacted in THF (0.8 mL) at 40 °C for 18 h, unless otherwise noted.

^b)^ Isolated yield.

^c)^ The diastereomeric ratio was determined by ^1^H NMR analysis of the crude mixture.

^d)^ DME was used instead of THF.

^e)^ The reaction was conducted without Cu(OAc)_2_.

^f)^ The reaction was conducted without Pd(dba)_2_. dba: dibenzylideneacetone; HBpin: 4,4,5,5‐tetramethyl‐1,3,2‐dioxaborolane.

With the optimized reaction conditions in hand, we explored the substrate scope of the hydroallylation (Scheme 2). Initially, a model reaction on 1 and 5 mmol scales confirmed that the diastereo‐ and enantioselectivities were comparable to those on the 0.2 mmol scale. The influence of α‐aryl groups on the allyl acetates was then investigated. Introducing a methyl group at the *para* or *meta* position of the phenyl group had no significant impact on yields or stereoselectivities, producing **3b** and **3c** with 85% yield each. In contrast, an *ortho*‐methyl group on the aryl group reduced reactivity, yielding **3d** with 69% but improved diastereoselectivity to >95:5. These results suggest that while steric hindrance on the aryl group negatively affects reactivity, it enhances diastereoselectivity. Allyl acetates with electron‐deficient aryl groups resulted in slightly lower yields and diastereoselectivities (**3e** and **3f**), whereas an electron‐rich aryl group afforded **3g** with a similar yield and stereoselectivities to **3a**. Consistent with the behavior observed for **3d**, the diastereoselectivity of **3h**, bearing a sterically hindered 1‐naphthyl group, increased to >95:5. Furthermore, a 2‐naphthyl‐substituted allyl acetate delivered **3i** in 73% yield with 86:14 dr and 99% ee, demonstrating that allyl acetates having polyaromatic groups are suitable substrates. Conversely, the diastereoselectivity of **3j** decreased slightly to 79:21, suggesting that a coordinating heteroatom on the aryl group may negatively influence diastereoselectivity. Notably, allyl acetates bearing α‐alkyl groups also produced **3k** and **3l**, albeit with slightly reduced yields of 58% and 54%, respectively. Next, we assessed the effect of the silyl group on the allyl acetate substrates. Interestingly, bulkier silyl groups, such as triethylsilyl, triisopropylsilyl, and dimethyl(phenyl)silyl, enhanced diastereoselectivities to >95:5 (**3m**–**o**). This indicates that steric hindrance from the silyl group likely plays a crucial role in the addition step, which is presumed to be the stereo‐determining step of the hydroallylation. In comparison, an allyl acetate with a benzyl(dimethyl)silyl group afforded **3p** with 91:9 dr, similar to the result observed for the trimethylsilyl‐substituted substrate.

**Scheme 2:**
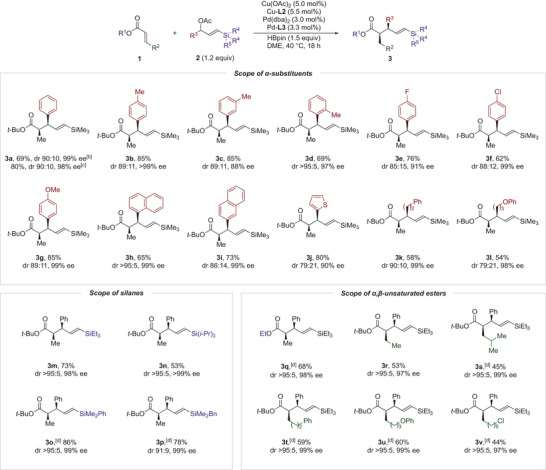
Substrate scope for regio‐ and enantioconvergent hydroallylation of acrylates with unsymmetrical 1,3‐disubstituted allyl acetates.^a)^ a) Reaction conditions: **1a** (0.2 mmol), **2a** (0.24 mmol), Cu(OAc)_2_ (5.0 mol%), Cu‐**L2** (5.5 mol%), Pd(dba)_2_ (3.0 mol%), Pd‐**L3** (3.3 mol%), and HBpin (0.3 mmol) were reacted in DME (0.8 mL) at 40 °C for 18 h, unless otherwise noted. b) 1 mmol scale. c) 5 mmol scale. d) THF was used instead of DME.

Subsequent experiments focused on expanding the scope of acrylates. Ethyl acrylate proved to be an effective substrate for this transformation, affording **3q** with 68% yield, >95:5 dr, and 98% ee. In contrast, β‐substituted acrylates resulted in reduced yields (**3r**–**v**), likely due to slower enolate formation compared to unsubstituted acrylates. Nonetheless, an acrylate with a bulky β‐substituent, such as an isopropyl group, successfully afforded **3s** with 45% yield. The presence of heteroatoms on the β‐alkyl chain had a negligible impact on stereoselectivity, delivering **3u** and **3v** with excellent stereoselectivities. Notably, a primary alkyl chloride group did not trigger any side reactions, such as dehalogenation or S_N_2 reaction. However, attempts to achieve hydroallylation with acrylates bearing β‐aryl substituents were unsuccessful, yielding only trace amounts or none of the desired products. Thus, while β‐aryl‐substituted acrylates remain a limitation of this method, this hydroallylation demonstrates broad applicability across a diverse range of allyl acetates and acrylates.

To demonstrate the utility and practicality of this process, we performed transformations of vinylsilane moiety in *tert*‐butyl 4‐pentenoate **3** (Scheme 3). Subjecting **3a** to acidic conditions effectively removed both the trimethylsilyl and the *tert*‐butyl groups. Subsequent methylation of the intermediate afforded methyl 4‐pentenoate **4** in 83% yield over two steps (Scheme 3a). Treatment of **3a** with acetic anhydride in the presence of a rhodium catalyst produced enone **5** in 60% yield, albeit with a slight decrease in diastereoselectivity (Scheme 3b).^[^ ^44^ ^]^ Finally, we conducted the arylation of vinylsilane. Using 4‐pentenoate **3p**, which features a benzyl(dimethyl)silyl group, in a palladium‐catalyzed arylation reaction successfully converted the silyl group into an aryl group, delivering the product **6** in 73% yield (Scheme 3c).^[^ ^46^ ^]^ Notably, this transformation enables the synthesis of an allylated product bearing two distinct aryl groups, a result that cannot be achieved regioselectively with previous asymmetric allylic substitution methods. These transformations of vinylsilane moieties clearly demonstrate the versatility of the silyl‐substituted allyl acetates developed in this study.

**Scheme 3:**
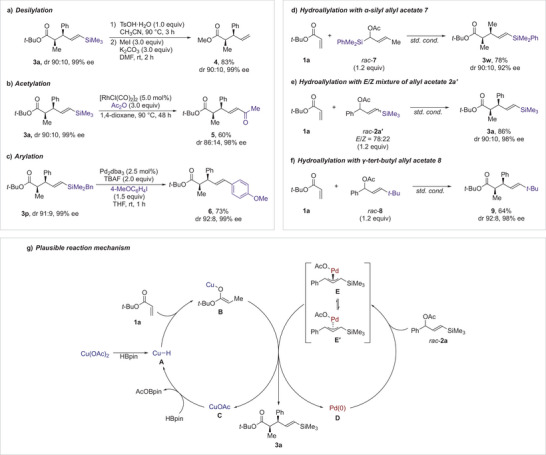
Derivatization of pent‐4‐enoate **3**, preliminary mechanistic investigations, and plausible reaction mechanism.

Next, a series of preliminary mechanistic investigations were performed to elucidate the reaction mechanism. A hydroallylation involving α‐dimethyl(phenyl)silyl‐γ‐methyl allyl acetate **7** yielded the γ‐allylated product **3w** exclusively, with a dr of 90:10 and an ee of 98% (Scheme 3d). Additionally, the use of an *E*/*Z* mixture of allyl acetate **2a** under the optimized reaction conditions resulted in the formation of the desired 4‐pentenoate **3a** without producing the *Z*‐isomer (Scheme 3e). These findings suggest that the hydroallylation proceeds via the formation of a common π‐allylpalladium species. Moreover, the optimized reaction conditions, which produce allylated products **3** with high yields and enantioselectivities using less than two equivalents of allyl acetate **2**, indicate that the lifetime of the π‐allylpalladium species is sufficiently long to allow interconversion between its enantiomers.^[^ ^35^, ^36^, ^37^, ^38^, ^39^ ^]^ Given the known α‐silicon effect,^[^ ^76^, ^77^, ^78^, ^79^, ^80^, ^81^, ^82^ ^]^ which stabilizes a carbon─metal bond at the α‐position of a silyl group, its influence on the hydroallylation was evaluated. To this end, γ‐*tert*‐butyl‐substituted allyl acetate **8** was subjected to the optimized reaction conditions, resulting in the exclusive formation of pentenoate **9** with a terminal *tert*‐butyl group (Scheme 3f). This outcome suggests that the stabilizing effect of a silyl group is minimal, while its steric bulk is crucial for controlling both regioselectivity and enantioselectivity.

Based on previous studies^[^ ^35^, ^36^, ^37^, ^38^, ^39^, ^55^, ^56^, ^57^, ^58^, ^59^, ^60^, ^61^, ^62^, ^63^, ^64^, ^65^, ^66^, ^67^, ^75^ ^]^ and preliminary mechanistic investigations, a reaction mechanism for the hydroallylation is proposed, as depicted in Scheme 3g. The mechanism begins with the reduction of the copper(II) acetate precatalyst to the active copper hydride species **A**. This species facilitates the reductive enolate formation of *tert*‐butyl acrylate (**1a**), producing copper enolate **B**.^[^ ^83^, ^84^, ^85^ ^]^ Simultaneously, the palladium(0) catalyst **D** generates the π‐allyl species **E** from allyl acetate **2**. One enantiomer of the π‐allyl species **E** preferentially reacts with copper enolate **B**, yielding the desired pentenoate **3a** with high regio‐ and stereoselectivities, along with the formation of copper(I) acetate **C** and the regeneration of palladium(0) **D**. Finally, a σ‐bond metathesis between copper(I) acetate **C** and pinacolborane regenerates copper hydride **A**. During the catalytic cycle, the interconversion between the enantiomers of the π‐allylpalladium species **E** and **E′** occurs,^[^ ^35^, ^36^, ^37^, ^38^, ^39^ ^]^ enabling the enantioconvergent hydroallylation while requiring less than two equivalents of allyl acetate **2**.

In conclusion, we have developed a hydroallylation of acrylates using α‐substituted γ‐silyl allyl acetate as novel allyl electrophiles, enabling precise control over both regio‐ and enantioselectivity. This method efficiently produces a diverse array of δ‐silyl‐4‐pentenoates with vicinal stereocenters, which serve as versatile intermediates, as demonstrated by their successful derivatization. Notably, the desilylative arylation allows access to an otherwise unattainable allylated product, facilitating the regioselective incorporation of two similar aryl groups at the β‐ and δ‐positions. Preliminary mechanistic investigations suggest that the reaction proceeds via a π‐allylpalladium species, which undergoes interconversion between two enantiomers during the catalytic cycle. Furthermore, the silyl group on the allyl acetate primarily exerts its influence through steric control rather than stabilization via an α‐silicon effect. Ongoing mechanistic studies in our laboratory aim to further elucidate the enantioconvergent process. The α‐substituted γ‐silyl allyl acetates developed in this work are anticipated to expand the scope of asymmetric allylic substitutions, opening new avenues for complex molecule synthesis.

## Supporting Information

The authors have cited additional references within the Supporting Information.^[^ ^86^, ^87^, ^88^, ^89^, ^90^, ^91^ ^]^

## Conflict of Interests

The authors declare no conflict of interest.

## Supporting information



Supporting Information

## Data Availability

The data that support the findings of this study are available in the supplementary material of this article.
