# Supplementary material for: Regio‐ and Enantioconvergent Hydroallylation of Acrylates Enabled by γ‐Silyl‐Substituted Allyl Acetates
Source: Angew Chem Int Ed Engl. 2025 Apr 21;64(19):e202425256. doi: 10.1002/anie.202425256 (PMC12051904; doi:10.1002/anie.202425256)
Supplement: Supplementary file 1 — Supporting Information [file ANIE-64-e202425256-s001.docx]

Supporting Information

**Regio- and Enantioconvergent Hydroallylation of Acrylates Enabled by γ-Silyl-Substituted Allyl Acetates**

Hirotsugu Suzuki, Ryoichi Nishikawa, Yuki Sato, Kaisei Sekino, and Takanori Matsuda

*Tenure-Track Program for Innovative Research, University of Fukui, 3-9-1 Bunkyo,*

*Fukui-shi, Fukui 910-8507, Japan*

*Department of Applied Chemistry, Tokyo University of Science, 1-3 Kagurazaka, Shinjuku-ku, Tokyo 162-8601, Japan*

E-mail: h-suzuki@u-fukui.ac.jp, mtd@rs.tus.ac.jp

Table of Contents

1. General Information S1
2. Preparation and Characterization of Starting Materials S2
3. Determination of Relative and Absolute Configurations of 4-Pentanoate **4** S19
4. Catalytic Regio- and Enantioselective Hydroallylation of Acrylates **1** using Allyl Acetates **2** S20
5. Derivatization of 4-Pentenoate **3** S31
6. Mechanistic Investigations S34
7. References S39
8. Copies of ^1^H, ^13^C and ^19^F NMR Spectra for the Products S40
9. Copies of HPLC Charts for the Products S144
10. **General Information**

All reactions were performed in an oven-dried flask using standard Schlenk techniques under an argon atmosphere, unless otherwise noted. ^1^H, ^13^C, and ^19^F NMR spectra were recorded on a JEOL ECA 500II spectrometer (500 MHz for ^1^H, 125 MHz for ^13^C, and 470 MHz for ^19^F) in CDCl_3_. Tetramethylsilane (TMS) served as an internal standard for ^1^H (δ = 0), CDCl_3_ served as an internal standard for ^13^C (δ = 77.0), and CF_3_CO_2_H served as an internal standard for ^19^F (δ = −76.55). IR spectra were recorded on a JASCO FT/IR-4600. ESI-MS were measured on a Bruker ESI-TOF-MS. Preparative thin-layer chromatography (PTLC) was performed using Wakogel^®^ B-5F, and flash column chromatography was performed using Wakogel^®^ C-200 (75–150 µm).

- 1. **Materials**

Cu(OAc)_2_ was purchased from FUJIFILM Wako Pure Chemicals Corporation. Pd(dba)_2_ was purchased from Tokyo Chemical Industry Co., Ltd. All chiral ligands (Cu-**L1**–**L6**) were purchased from Tokyo Chemical Industry Co., Ltd. XPhos (Pd-**L1**) was purchased from BLD Pharmatech Ltd. *t*-BuXPhos (Pd-**L2**) and RuPhos (Pd-**L3**) were purchased from Tokyo Chemical Industry Co., Ltd. Pinacolborane (HBpin) was purchased from Kanto Chemical Co., Inc. and distilled just before use. *tert*-Butyl and ethyl acrylates were purchased from Tokyo Chemical Industry Co., Ltd. and distilled just before use. Tetrahydrofuran (THF) was purchased from Kanto Chemical Co., Inc. as part of the “Dehydrated Solvent System.” 1,2-Dimethoxyethane (DME) was purchased from Tokyo Chemical Industry Co., Ltd. and stored with 4Å molecular sieves.

1. **Preparation and Characterization of Starting Materials**
   - 1. **General Procedure for Allyl Acetates 2a–n and 8**

**Procedure for the preparation of S1**: Propargyl alcohol **S1** was synthesized according to a modified literature procedure.^[86]^ To an oven-dried two-necked 50 mL flask equipped with a magnetic stirring bar, trimethylsilylacetylene (1.9 mL, 14 mmol) and THF (30 mL) were added. The solution was cooled to 0 °C (−78 °C for **2l**), and *n*-BuLi (8.1 mL, 13 mmol, 1.6 M in hexane) was added dropwise. After the completion of the addition, the reaction mixture was stirred at the same temperature for 1 h, followed by the addition of benzaldehyde (1.05 g, 9.94 mmol). After 1 h, the mixture was quenched with a saturated NaHCO_3_ aqueous solution. The aqueous phase was extracted with EtOAc (3 × 10 mL), washed with brine (10 mL), dried over anhydrous Na_2_SO_4_, and concentrated in vacuo to afford propargyl alcohol **S1** as a colorless oil, which was used without further purification in the next step.

**Procedure for the preparation of S2**: Allylic alcohol **S2** was synthesized according to a modified literature procedure.^[86]^ To an oven-dried two-necked 50 mL flask equipped with a magnetic stirring bar, the resulting colorless oil and Et_2_O (20 mL) were added. The reaction mixture was cooled to 0 °C, and Red-Al (4.7 mL, 15 mmol, 3.6 M in toluene) was added slowly. The mixture was then warmed to room temperature and stirred for 12 h. After the careful addition of H_2_O (10 mL), the aqueous layer was extracted with EtOAc (3 × 10 mL), washed with brine (10 mL), dried over anhydrous Na_2_SO_4_, and concentrated in vacuo to afford allylic alcohol **S2** as a colorless oil, which was used without further purification in the next step.

**Procedure for the preparation of 2a**–**n**: Allyl acetates **2a**–**n** and **8** were synthesized according to a modified literature procedure.^[86]^ An oven-dried two-necked 50 mL flask equipped with a magnetic stirring bar was charged with allylic alcohol **S2** (1.43 g, 6.93 mmol), DMAP (82.8 mg, 0.678 mmol), CH_2_Cl_2_ (20 mL), and Et_3_N (1.93 mL, 13.9 mmol). The solution was cooled to 0 °C, and acetic anhydride (0.707 g, 6.92 mmol) was added. The reaction mixture was stirred at room temperature for 1 h and then quenched with a saturated NH_4_Cl aqueous solution. The aqueous layer was extracted with EtOAc (3 × 10 mL), washed with brine (10 mL), dried over anhydrous Na_2_SO_4_, and concentrated in vacuo. The crude product was purified by flash column chromatography (hexane:EtOAc = 20:1) to afford allyl acetate **2a** as a colorless oil (1.29 g, 5.10 mmol, 51% yield over three steps).

- - 1. **Characterization of Allyl Acetates 2a–n and 8**

**(*E*)-1-Phenyl-3-(trimethylsilyl)allyl acetate (2a):**

Allyl acetate **2a** was obtained as a colorless oil (1.29 g, 5.10 mmol, 51% yield over three steps) after purification by flash column chromatography (hexane:EtOAc = 20:1); ^1^H NMR (500 MHz, CDCl_3_) δ: 7.45–7.33 (m, 5H), 6.30 (dd, *J* = 4.9, 1.4 Hz, 1H), 6.18 (dd, *J* = 18.3, 5.2 Hz, 1H), 5.96 (dd, *J* = 18.6, 1.4 Hz, 1H), 2.17 (s, 3H), 0.12 (s, 9H); ^13^C NMR (125 MHz, CDCl_3_) δ: 170.0, 142.8, 139.0, 132.0, 128.5, 128.0, 127.3, 77.6, 21.3, −1.4; IR (neat): 2984, 2955, 1743, 1669, 1370, 1259, 1235, 1023, 989, 874, 842, 761, 699 cm^−1^; HRMS (ESI-TOF): calcd for C_14_H_20_NaO_2_Si^+^: [M + Na]^+^ = 271.1125, found 271.1128.

**(*E*)-1-(*p*-Tolyl)-3-(trimethylsilyl)allyl acetate (2b):**

Allyl acetate **2b** was obtained as a colorless oil (968.1 mg, 3.69 mmol, 36% yield over three steps) after purification by flash column chromatography (hexane:Et_2_O = 40:1); ^1^H NMR (500 MHz, CDCl_3_) δ: 7.28 (d, *J* = 8.0 Hz, 2H), 7.22 (d, *J* = 8.0 Hz, 2H), 6.27 (dd, *J* = 5.2, 1.1 Hz, 1H), 6.18 (dd, *J* = 18.9, 5.2 Hz, 1H), 5.95 (dd, *J* = 18.6, 1.4 Hz, 1H), 2.40 (s, 3H), 2.16 (s, 3H), 0.12 (s, 9H); ^13^C NMR (125 MHz, CDCl_3_) δ: 170.0, 143.0, 137.9, 136.1, 131.6, 129.2, 127.4, 77.4, 21.3, 21.2, −1.4; IR (neat): 3026, 3001, 2955, 1745, 1371, 1233, 1052, 1018, 989, 958, 842 cm^−1^; HRMS (ESI-TOF): calcd for C_15_H_22_NaO_2_Si^+^: [M + Na]^+^ = 285.1281, found 285.1283.

**(*E*)-1-(*m*-Tolyl)-3-(trimethylsilyl)allyl acetate (2c):**

Allyl acetate **2c** was obtained as a colorless oil (981.9 mg, 3.74 mmol, 38% yield over three steps) after purification by flash column chromatography (hexane:Et_2_O = 40:1); ^1^H NMR (500 MHz, CDCl_3_) δ: 7.32–7.28 (m, 1H), 7.21–7.16 (m, 3H), 6.26 (d, *J* = 5.2 Hz, 1H), 6.17 (dd, *J* = 18.3, 5.2 Hz, 1H), 5.95 (dd, *J* = 18.6, 1.4 Hz, 1H), 2.41 (s, 3H), 2.17 (s, 3H), 0.12 (s, 9H); ^13^C NMR (125 MHz, CDCl_3_) δ: 170.0, 143.0, 139.0, 138.2, 131.7, 128.8, 128.4, 128.0, 124.3, 77.6, 21.4, 21.3, −1.4; IR (neat): 3026, 2999, 2955, 1746, 1370, 1235, 1020, 988, 867, 842, 784, 702 cm^−1^; HRMS (ESI-TOF): calcd for C_15_H_22_NaO_2_Si^+^: [M + Na]^+^ = 285.1281, found 285.1278.

**(*E*)-1-(*o*-Tolyl)-3-(trimethylsilyl)allyl acetate (2d):**

Allyl acetate **2d** was obtained as a colorless oil (1.03 g, 3.94 mmol, 40% yield over three steps) after purification by flash column chromatography (hexane:Et_2_O = 40:1); ^1^H NMR (500 MHz, CDCl_3_) δ: 7.41–7.37 (m, 1H), 7.29–7.25 (m, 2H), 7.24–7.21 (m, 1H), 6.49 (dd, *J* = 4.9, 1.4 Hz, 1H), 6.17 (dd, *J* = 18.6, 4.9 Hz, 1H), 5.89 (dd, *J* = 18.9, 1.7 Hz, 1H), 2.42 (s, 3H), 2.18 (s, 3H), 0.12 (s, 9H); ^13^C NMR (125 MHz, CDCl_3_) δ: 170.0, 142.2, 137.2, 136.0, 132.0, 130.5, 127.9, 127.1, 126.1, 74.9, 21.3, 19.3, −1.4; IR (neat): 3025, 2998, 2955, 2898, 1743, 1371, 1237, 1019, 989, 959, 842, 759, 729 cm^−1^; HRMS (ESI-TOF): calcd for C_15_H_22_NaO_2_Si^+^: [M + Na]^+^ = 285.1281, found 285.1283.

**(*E*)-1-(4-Fluorophenyl)-3-(trimethylsilyl)allyl acetate (2e):**

Allyl acetate **2e** was obtained as a colorless oil (1.12 g, 4.22 mmol, 43% yield over three steps) after purification by flash column chromatography (hexane:Et_2_O = 40:1); ^1^H NMR (500 MHz, CDCl_3_) δ: 7.39–7.32 (m, 2H), 7.13–7.05 (m, 2H), 6.27 (dd, *J* = 5.2, 1.1 Hz, 1H), 6.15 (dd, *J* = 18.6, 4.9 Hz, 1H), 5.93 (dd, *J* = 18.6, 1.4 Hz, 1H), 2.16 (s, 3H), 0.12 (s, 9H); ^13^C NMR (125 MHz, CDCl_3_) δ: 169.9, 162.4 (d, ^1^*J*_C–F_ = 247.1 Hz), 142.6, 134.8 (d, ^4^*J*_C–F_ = 3.6 Hz), 132.2, 129.1 (d, ^3^*J*_C–F_ = 8.4 Hz), 115.4 (d, ^2^*J*_C–F_ = 21.6 Hz), 76.8, 21.2, −1.5; ^19^F NMR (470 MHz, CDCl_3_) δ: −114.7; IR (neat): 3032, 2956, 2898, 1746, 1606, 1512, 1371, 1234, 1158, 1017, 989, 841 cm^−1^; HRMS (ESI-TOF): calcd for C_14_H_19_FNaO_2_Si^+^: [M + Na]^+^ = 289.1031, found 289.1018.

**(*E*)-1-(4-Chlorophenyl)-3-(trimethylsilyl)allyl acetate (2f):**

Allyl acetate **2f** was obtained as a colorless oil (903.5 mg, 3.19 mmol, 32% yield over three steps) after purification by flash column chromatography (hexane:Et_2_O = 50:1); ^1^H NMR (500 MHz, CDCl_3_) δ: 7.38 (d, *J* = 8.0 Hz, 2H), 7.33–7.29 (m, 2H), 6.25 (dd, *J* = 5.2, 1.1 Hz, 1H), 6.14 (dd, *J* = 18.9, 5.2 Hz, 1H), 5.94 (dd, *J* = 18.9, 1.1 Hz, 1H), 2.17 (s, 3H), 0.12 (s, 9H); ^13^C NMR (125 MHz, CDCl_3_) δ: 169.8, 142.4, 137.6, 133.9, 133.2, 132.6, 128.7, 76.8, 21.2, −1.5; IR (neat): 2999, 2955, 2898, 1743, 1494, 1371, 1236, 1092, 1054, 1015, 989, 842, 539 cm^−1^; HRMS (ESI-TOF): calcd for C_14_H_19_ClNaO_2_Si^+^: [M + Na]^+^ = 305.0735, found 305.0746.

**(*E*)-1-(4-Methoxyphenyl)-3-(trimethylsilyl)allyl acetate (2g):**

Allyl acetate **2g** was obtained as a colorless oil (1.11 g, 4.00 mmol, 40% yield over three steps) after purification by flash column chromatography (hexane:Et_2_O = 20:1); ^1^H NMR (500 MHz, CDCl_3_) δ: 7.26 (d, *J* = 4.0 Hz, 2H), 6.94 (d, *J* = 8.6 Hz, 2H), 6.26 (dd, *J* = 4.9, 1.4 Hz, 1H), 6.18 (dd, *J* = 18.6, 4.9 Hz, 1H), 5.93 (dd, *J* = 18.6, 1.4 Hz, 1H), 3.86 (s, 3H), 2.15 (s, 3H), 0.12 (s, 9H); ^13^C NMR (125 MHz, CDCl_3_) δ: 170.0, 159.4, 143.0, 131.5, 131.2, 128.9, 113.9, 77.2, 55.3, 21.3, −1.4; IR (neat): 2999, 2955, 2901, 1744, 1613, 1515, 1371, 1250, 1175, 1037, 841 cm^−1^; HRMS (ESI-TOF): calcd for C_15_H_22_NaO_3_Si^+^: [M + Na]^+^ = 301.1230, found 301.1233.

**(*E*)-1-(Naphthalen-1-yl)-3-(trimethylsilyl)allyl acetate (2h):**

Allyl acetate **2h** was obtained as a colorless solid (1.21 g, 4.07 mmol, 41% yield over three steps) after purification by flash column chromatography (hexane:Et_2_O = 40:1); mp: 50.1–50.9 °C; ^1^H NMR (500 MHz, CDCl_3_) δ: 8.18 (d, *J* = 8.0 Hz, 1H), 7.97–7.88 (m, 2H), 7.64–7.51 (m, 4H), 7.04 (dd, *J* = 4.6, 1.7 Hz, 1H), 6.36 (dd, *J* = 18.9, 4.6 Hz, 1H), 6.02 (dd, *J* = 18.9, 1.7 Hz, 1H), 2.22 (s, 3H), 0.12 (s, 9H); ^13^C NMR (125 MHz, CDCl_3_) δ: 170.1, 142.5, 134.7, 133.9, 132.1, 130.8, 128.9, 128.7, 126.2, 125.7, 125.7, 125.3, 124.0, 75.0, 21.2, −1.4; IR (neat): 3001, 2955, 2902, 1740, 1613, 1515, 1371, 1304, 1250, 1175, 1037, 842 cm^−1^; HRMS (ESI-TOF): calcd for C_18_H_22_NaO_2_Si^+^: [M + Na]^+^ = 321.1281, found 321.1274.

**(*E*)-1-(Naphthalen-2-yl)-3-(trimethylsilyl)allyl acetate (2i):**

Allyl acetate **2i** was obtained as a colorless oil (1.12 g, 3.77 mmol, 40% yield over three steps) after purification by flash column chromatography (hexane:Et_2_O = 50:1); ^1^H NMR (500 MHz, CDCl_3_) δ: 7.90–7.80 (m, 4H), 7.54–7.44 (m, 3H), 6.45 (d, *J* = 5.2 Hz, 1H), 6.25 (dd, *J* = 18.6, 4.9 Hz, 1H), 5.99 (dd, *J* = 19.5, 1.7 Hz, 1H), 2.18 (s, 3H), 0.11 (s, 9H); ^13^C NMR (125 MHz, CDCl_3_) δ: 170.0, 142.9, 136.4, 133.1, 133.1, 132.3, 128.3, 128.0, 127.6, 126.4, 126.2, 126.2, 125.0, 77.7, 21.3, −1.4; IR (neat): 3064, 2998, 2954, 1741, 1372, 1250, 1233, 1020, 990, 868, 841 cm^−1^; HRMS (ESI-TOF): calcd for C_18_H_22_NaO_2_Si^+^: [M + Na]^+^ = 321.1281, found 321.1271.

**(*E*)-1-(Thiophen-2-yl)-3-(trimethylsilyl)allyl acetate (2j):**

Allyl acetate **2j** was obtained as a pale yellow oil (790.8 mg, 3.11 mmol, 61% yield over three steps) after purification by flash column chromatography (hexane:EtOAc = 30:1); ^1^H NMR (500 MHz, CDCl_3_) δ: 7.32 (d, *J* = 5.2 Hz, 1H), 7.07 (d, *J* = 3.4 Hz, 1H), 7.01 (dd, *J* = 4.6, 3.4 Hz, 1H), 6.54 (d, *J* = 4.6 Hz, 1H), 6.22 (dd, *J* = 19.5, 5.2 Hz, 1H), 6.04 (d, *J* = 18.9 Hz, 1H), 2.14 (s, 3H), 0.12 (s, 9H); ^13^C NMR (125 MHz, CDCl_3_) δ: 168.8, 142.0, 141.8, 132.6, 126.7, 126.5, 126.0, 72.5, 21.2, −1.5; IR (neat): 3001, 2955, 2898, 1747, 1370, 1232, 1017, 987, 869, 842, 705 cm^−1^; HRMS (ESI-TOF): calcd for C_12_H_18_NaO_2_SSi^+^: [M + Na]^+^ = 277.0689, found 277.0677.

**(*E*)-5-Phenyl-1-(trimethylsilyl)pent-1-en-3-yl acetate (2k):**

Allyl acetate **2k** was obtained as a colorless oil (554.5 mg, 2.01 mmol, 39% yield over three steps) after purification by flash column chromatography (hexane:EtOAc = 50:1); ^1^H NMR (500 MHz, CDCl_3_) δ: 7.35–7.32 (m, 2H), 7.27–7.20 (m, 3H), 6.00 (dd, *J* = 18.9, 5.2 Hz, 1H), 5.91 (dd, *J* = 18.9, 1.1 Hz, 1H), 5.33 (dt, *J* = 14.5, 3.2 Hz, 1H), 2.75–2.62 (m, 2H), 2.13 (s, 3H), 2.07–1.88 (m, 2H), 0.12 (s, 9H); ^13^C NMR (125 MHz, CDCl_3_) δ: 170.3, 143.2, 131.7, 128.4, 128.3, 125.9, 75.6, 35.7, 35.7, 31.5, 21.2, −1.4; IR (neat): 3027, 2990, 2954, 1743, 1372, 1248, 1025, 989, 866, 841, 699 cm^−1^; HRMS (ESI-TOF): calcd for C_16_H_24_NaO_2_Si^+^: [M + Na]^+^ = 299.1438, found 299.1438.

**(*E*)-6-Phenoxy-1-(trimethylsilyl)hex-1-en-3-yl acetate (2l):**

Allyl acetate **2l** was obtained as a colorless oil (1.39 g, 4.52 mmol, 45% yield over three steps) after purification by flash column chromatography (hexane:EtOAc = 4:1); ^1^H NMR (500 MHz, CDCl_3_) δ: 7.35–7.30 (m, 2H), 7.01–6.97 (m, 1H), 6.94 (d, *J* = 8.6 Hz, 2H), 6.00 (dd, *J* = 18.9, 4.9 Hz, 1H), 5.92 (dd, *J* = 18.9 Hz, 1H), 5.37 (d, *J* = 4.6 Hz, 1H), 4.02 (d, *J* = 2.9 Hz, 2H), 2.14 (s, 3H), 1.88–1.84 (m, 4H), 0.12 (s, 9H); ^13^C NMR (125 MHz, CDCl_3_) δ: 170.3, 158.9, 143.2, 131.8, 129.4, 120.6, 114.4, 75.6, 67.3, 30.7, 25.0, 21.2, −1.4; IR (neat): 3031, 2993, 2955, 1743, 1602, 1498, 1473, 1372, 1247, 1032, 867, 842 cm^−1^; HRMS (ESI-TOF): calcd for C_17_H_26_NaO_3_Si^+^: [M + Na]^+^ = 329.1543, found 329.1547.

**(*E*)-1-Phenyl-3-(triethylsilyl)allyl acetate (2m):**

Allyl acetate **2m** was obtained as a colorless oil (2.49 g, 8.58 mmol, 86% yield over three steps) after purification by flash column chromatography (hexane:EtOAc = 30:1); ^1^H NMR (500 MHz, CDCl_3_) δ: 7.35–7.30 (m, 5H), 6.26 (d, *J* = 5.2 Hz, 1H), 6.14 (dd, *J* = 18.9, 5.2 Hz, 1H), 5.85 (d, *J* = 18.9 Hz, 1H), 2.12 (s, 3H), 0.91 (t, *J* = 8.0 Hz, 9H), 0.57 (q, *J* = 7.8 Hz, 6H); ^13^C NMR (125 MHz, CDCl_3_) δ: 170.0, 144.1, 139.1, 128.5, 128.2, 128.0, 127.4, 77.7, 21.3, 7.3, 3.3; IR (neat): 3032, 2954, 2911, 2875, 1745, 1456, 1370, 1235, 1018, 771, 724, 699 cm^−1^; HRMS (ESI-TOF): calcd for C_17_H_26_NaO_2_Si^+^: [M + Na]^+^ = 313.1594, found 313.1588.

**(*E*)-1-Phenyl-3-(triisopropylsilyl)allyl acetate (2n):**

Allyl acetate **2n** was obtained as a colorless oil (1.04 g, 3.14 mmol, 63% yield over three steps) after purification by flash column chromatography (hexane:EtOAc = 50:1); ^1^H NMR (500 MHz, CDCl_3_) δ: 7.38–7.27 (m, 5H), 6.27 (dd, *J* = 5.2, 1.1 Hz, 1H), 6.18 (dd, *J* = 18.9, 5.7 Hz, 1H), 5.83 (dd, *J* = 18.9, 1.1 Hz, 1H), 2.12 (s, 3H), 1.15–1.01 (m, 21H); ^13^C NMR (125 MHz, CDCl_3_) δ: 169.9, 144.8, 139.1, 128.5, 128.0, 127.3, 126.3, 77.9, 21.3, 18.5, 10.8; IR (neat): 3033, 2942, 2892, 2866, 1744, 1463, 1370, 1233, 1017, 994, 883 cm^−1^; HRMS (ESI-TOF): calcd for C_20_H_32_NaO_2_Si^+^: [M + Na]^+^ = 355.2064, found 355.2053.

**(*E*)-1-Phenyl-3-(trimethylsilyl)allyl acetate (8):**

The title compound was prepared using the general scheme, substituting 3,3-dimethylbut-1-yne for silyl acetylene. Allyl acetate **8** was obtained as a colorless oil (1.26 g, 5.41 mmol, 52% yield over three steps) after purification by flash column chromatography (hexane:EtOAc = 3:1); ^1^H NMR (500 MHz, CDCl_3_) δ: 7.36–7.25 (m, 5H), 6.24 (d, *J* = 6.9 Hz, 1H), 5.76 (d, *J* = 16.0 Hz, 1H), 5.53 (dd, *J* = 15.5, 6.9 Hz, 1H), 2.10 (s, 3H), 1.01 (s, 9H); ^13^C NMR (125 MHz, CDCl_3_) δ: 170.0, 145.3, 140.0, 128.4, 127.8, 126.9, 123.2, 76.4, 33.0, 29.3, 21.4; IR (neat): 3034, 2961, 2904, 2869, 1741, 1369, 1238, 1019, 972, 702 cm^−1^; HRMS (ESI-TOF): calcd for C_15_H_20_NaO_2_^+^: [M + Na]^+^ = 255.1356, found 255.1355.

- - 1. **General Procedure for Allyl Acetates 2o and 2p**

**Procedure for the preparation of S3**: Propargyl alcohol **S3** was synthesized according to a literature procedure.^[87]^ To an oven-dried two-necked 50 mL flask equipped with a magnetic stirring bar, 2-propyn-1-ol (565.8 mg, 10.1 mmol) and THF (20 mL) were added. The solution was cooled to −78 °C, and *n*-BuLi (13 mL, 21 mmol, 1.6 M in hexane) was added dropwise. After the completion of the addition, the reaction mixture was warmed to room temperature and stirred for 1.5 h. The mixture was then cooled again to −78 °C, followed by the slow addition of benzylchlorodimethylsilane (1.83 g, 9.91 mmol). The reaction was warmed to room temperature and stirred overnight. The reaction mixture was quenched with 1 M HCl aqueous solution, and the aqueous phase was extracted with EtOAc (3 × 10 mL), washed with brine (10 mL), dried over anhydrous Na_2_SO_4_, and concentrated in vacuo to afford propargyl alcohol **S3** as a colorless oil, which was used without further purification in the next step.

**Procedure for the preparation of S4**: Allylic alcohol **S4** was synthesized according to a literature procedure.^[87]^ To an oven-dried two-necked 50 mL flask equipped with a magnetic stirring bar, the resulting colorless oil and Et_2_O (20 mL) were added. The reaction mixture was cooled to 0 °C, and Red-Al (6.4 mL, 23 mmol, 3.6 M in toluene) was added slowly. The mixture was then warmed to room temperature and stirred for 2 h. After careful addition of H_2_O (10 mL) and 3.6 M H_2_SO_4_ aqueous solution (10 mL), the aqueous layer was extracted with Et_2_O (3 × 10 mL), washed with brine (10 mL), dried over anhydrous Na_2_SO_4_, and concentrated in vacuo. The crude mixture was purified by flash column chromatography (hexane:EtOAc = 4:1) to afford allylic alcohol **S4** as a colorless oil (1.43 g, 6.92 mmol, 70% yield over two steps).

**Procedure for the preparation of S5**: Aldehyde **S5** was synthesized according to a modified literature procedure.^[88]^ Allylic alcohol **S4** (1.43 g, 6.92 mmol), manganese oxide (6.09 g, 70.1 mmol), and CH_2_Cl_2_ (40 mL) were placed in a one-necked 100 mL flask equipped with a magnetic stirring bar. The suspension was stirred vigorously at room temperature for 1 h and then filtered through a Celite pad. The filtrate was concentrated in vacuo to afford aldehyde **S5** as a colorless oil, which was used without further purification in the next step.

**Procedure for the preparation of S6**: Allyl alchol **S6** was synthesized according to a modified literature procedure.^[86]^ To an oven-dried three-necked 100 mL flask equipped with a magnetic stirring bar, magnesium turnings (230.6 mg, 9.49 mmol), iodine (one piece), and THF (5 mL) were added. Bromobenzene (0.93 mL, 8.9 mmol) in THF (5 mL) was added dropwise to the mixture and stirred for 1 h. The resulting deep gray solution was then cooled to 0 °C, and the aldehyde **S5** (1.21 g, 5.92 mmol) in THF (10 mL) was added dropwise. The reaction mixture was stirred overnight at room temperature and then quenched with a saturated NH_4_Cl aqueous solution. The aqueous layer was extracted with EtOAc (3 × 10 mL), washed with brine (10 mL), dried over anhydrous Na_2_SO_4_, and concentrated in vacuo. The crude mixture was purified by flash column chromatography (hexane:EtOAc = 4:1) to afford allylic alcohol **S6** as a colorless oil (1.41 g, 4.99 mmol, 72% yield over two steps).

**Procedure for the preparation of 2o and 2p**: Allyl acetates **2o** and **2p** were synthesized according to a modified literature procedure.^[89]^ An oven-dried two-necked 50 mL flask equipped with a magnetic stirring bar was charged with allylic alcohol **S6** (1.41 g, 4.99 mmol), DMAP (63.8 mg, 0.522 mmol), THF (20 mL), and Et_3_N (1.4 mL, 10 mmol). The solution was cooled to 0 °C, and acetic anhydride (0.71 mL, 7.5 mmol) was added dropwise. The reaction mixture was stirred at room temperature for 3 h and then concentrated in vacuo. The crude product was purified by flash column chromatography (hexane:EtOAc = 19:1) to afford allyl acetate **2p** as a colorless oil (1.27 g, 3.91 mmol, 78%).

- - 1. **Characterization of Allyl Acetates 2o and 2p**

**(*E*)-3-[Dimethyl(phenyl)silyl]-1-phenylallyl acetate (2o):**

Allylic alcohol **S4** was prepared according to the literature.^[86]^ Allylic alcohol **S6** was obtained as a colorless oil (1.31 g, 3.95 mmol, 69% over two steps) after purification by flash column chromatography (hexane:EtOAc = 19:1). Allyl acetate **2o** was obtained as a colorless oil (874.2 mg, 2.35 mmol, 59%) after purification by flash column chromatography (hexane:EtOAc = 9:1); ^1^H NMR (500 MHz, CDCl_3_) δ: 7.52–7.45 (m, 2H), 7.39–7.29 (m, 8H), 6.29 (t, *J* = 4.6 Hz, 1H), 6.25–6.18 (m, 1H), 6.04 (dd, *J* = 17.2, 1.1 Hz, 1H), 2.11 (s, 3H), 0.35–0.33 (m, 6H); ^13^C NMR (125 MHz, CDCl_3_) δ: 169.9, 144.7, 138.8, 138.1, 133.8, 129.4, 129.1, 128.5, 128.1, 127.8, 127.4, 77.4, 21.3, −2.7, −2.7; IR (neat): 3064, 3031, 3006, 2956, 1743, 1428, 1371, 1240, 1115, 1020, 989, 840 cm^−1^; HRMS (ESI-TOF): calcd for C_19_H_22_NaO_2_Si^+^: [M + Na]^+^ = 333.1281, found 333.1274.

**(*E*)-3-(benzyldimethylsilyl)-1-phenylallyl acetate (2p):**

Allylic alcohol **S4** was obtained as a colorless oil (1.43 g, 6.92 mmol, 70% yield over two steps) after purification by flash column chromatography (hexane:EtOAc = 4:1). Allylic alcohol **S6** was obtained as a colorless oil (1.41 g, 4.99 mmol, 72% yield over two steps) after purification by flash column chromatography (hexane:EtOAc = 4:1). Allyl acetate **2p** was obtained as a colorless oil (1.27 g, 3.91 mmol, 78%) after purification by flash column chromatography (hexane:EtOAc = 19:1); ^1^H NMR (500 MHz, CDCl_3_) δ: 7.37–7.28 (m, 5H), 7.16 (t, *J* = 7.7 Hz, 2H), 7.05 (t, *J* = 7.4 Hz, 1H), 6.94 (d, *J* = 7.4 Hz, 2H), 6.23 (dd, *J* = 5.2, 1.1 Hz, 1H), 6.08 (dd, *J* = 18.9, 5.2 Hz, 1H), 5.85 (dd, *J* = 18.6, 1.4 Hz, 1H), 2.12 (s, 2H), 2.11 (s, 3H), 0.05 (s, 3H), 0.05 (s, 3H); ^13^C NMR (125 MHz, CDCl_3_) δ: 169.9, 144.1, 139.5, 138.8, 129.6, 128.5, 128.2, 128.2, 128.1, 127.3, 124.0, 77.4, 25.8, 21.2, −3.6; IR (neat): 3060, 3026, 2956, 2894, 1740, 1601, 1493, 1370, 1232 cm^−1^; HRMS (Dart): calcd for C_20_H_25_O_2_Si^+^: [M + H]^+^ = 325.1618, found 325.1601.

- - 1. **General Procedure for α,β-Unsaturated Esters 1b–f**

**Procedure for the preparation of 1b**-**f**: *tert*-Butyl (triphenylphosphoranylidene)acetate was prepared according to the literature.^[90]^ α,β-Unsaturated esters **1b**–**f** were synthesized according to a modified literature procedure.^[90]^ To an oven-dried two-necked 50 mL flask equipped with a magnetic stirring bar, 3-phenylpropionaldehyde (1.34 g, 9.99 mmol) and dichloromethane (10 mL) were added. The reaction mixture was cooled to 0 °C, and *tert*-butyl (triphenylphosphoranylidene)acetate (4.52 g, 12.0 mmol) was added. The mixture was then warmed to room temperature, stirred overnight, and concentrated in vacuo. The crude product was purified by flash column chromatography (hexane:EtOAc = 3:1) to afford α,β-unsaturated ester **1d** as a colorless oil (2.13 g, 9.15 mmol, 92% yield).

- - 1. **Characterization of α,β-Unsaturated esters 1b–f**

***tert*-Butyl (*E*)-but-2-enoate (1b):**

α,β-Unsaturated esters **1b** was obtained as a colorless oil (573.6 mg, 4.03 mmol, 55% yield) after purification by flash column chromatography (hexane:EtOAc = 3:1); ^1^H NMR (500 MHz, CDCl_3_) δ: 6.90–6.83 (m, 1H), 5.78–5.74 (m, 1H), 1.84 (dd, *J* = 6.9, 1.7 Hz, 3H), 1.48 (s, 9H); ^13^C NMR (125 MHz, CDCl_3_) δ: 166.0, 143.1, 124.5, 79.9, 28.1, 17.7; IR (neat): 3006, 2978, 2935, 2920, 1718, 1658, 1368, 1321, 1292, 1166, 1102, 995 cm^−1^; HRMS (ESI-TOF): calcd for C_8_H_14_NaO_2_^+^: [M + Na]^+^ = 165.0886, found 165.0889.

***tert*-Butyl (*E*)-4-methylpent-2-enoate (1c):**

α,β-Unsaturated esters **1c** was obtained as a colorless oil (181.0 mg, 1.06 mmol, 21% yield) after purification by flash column chromatography (hexane:EtOAc = 3:1); ^1^H NMR (500 MHz, CDCl_3_) δ: 6.84 (dd, *J* = 15.8, 6.6 Hz, 1H), 5.69 (d, *J* = 15.5 Hz, 1H), 2.49–2.37 (m, 1H), 1.48 (s, 9H), 1.05 (d, *J* = 6.9 Hz, 6H); ^13^C NMR (125 MHz, CDCl_3_) δ: 166.5, 154.2, 120.3, 80.0, 30.8, 28.1, 21.3; IR (neat): 2968, 2933, 2872, 1715, 1653, 1367, 1347, 1302, 1157, 978 cm^−1^; HRMS (ESI-TOF): calcd for C_10_H_18_NaO_2_^+^: [M + Na]^+^ = 193.1199, found 193.1194.

***tert*-Butyl (*E*)-5-phenylpent-2-enoate (1d):**

α,β-Unsaturated esters **1d** was obtained as a colorless oil (2.13 g, 9.15 mmol, 92% yield) after purification by flash column chromatography (hexane:EtOAc = 3:1); ^1^H NMR (500 MHz, CDCl_3_) δ: 7.31–7.27 (m, 2H), 7.22–7.17 (m, 3H), 6.93–6.88 (m, 1H), 5.80–5.76 (m, 1H), 2.76 (t, *J* = 8.0 Hz, 2H), 2.52–2.46 (m, 2H), 1.48 (s, 9H); ^13^C NMR (125 MHz, CDCl_3_) δ: 166.0, 146.8, 128.4, 128.3, 126.1, 123.5, 99.9, 80.1, 34.4, 33.8, 28.1; IR (neat): 3030, 2978, 2931, 1716, 1653, 1455, 1367, 1322, 1255, 1148, 700 cm^−1^; HRMS (ESI-TOF): calcd for C_15_H_20_NaO_2_^+^: [M + Na]^+^ = 255.1356, found 255.1358.

***tert*-Butyl (*E*)-6-phenoxyhex-2-enoate (1e):**

α,β-Unsaturated esters **1e** was obtained as a colorless oil (845.7 mg, 3.22 mmol, 44% yield) after purification by flash column chromatography (hexane:EtOAc = 3:1); ^1^H NMR (500 MHz, CDCl_3_) δ: 7.32–7.24 (m, 2H), 6.97–6.83 (m, 4H), 5.79 (dt, *J* = 15.6, 1.6 Hz, 1H), 3.98 (t, *J* = 6.2 Hz, 2H), 2.43–2.32 (m, 2H), 2.01–1.86 (m, 2H), 1.48 (s, 9H); ^13^C NMR (125 MHz, CDCl_3_) δ: 166.0, 158.8, 146.8, 129.4, 123.6, 120.7, 114.4, 80.1, 66.7, 28.6, 28.1, 27.8; IR (neat): 3005, 2978, 2934, 2873, 1714, 1654, 1601, 1498, 1472, 1291, 1250, 1157 cm^−1^; HRMS (ESI-TOF): calcd for C_16_H_22_NaO_3_^+^: [M + Na]^+^ = 285.1461, found 285.1465.

***tert*-Butyl (*E*)-8-chlorooct-2-enoate (1f):**

α,β-Unsaturated esters **1f** was obtained as a colorless oil (517.6 mg, 1.78 mmol, 36% yield) after purification by flash column chromatography (hexane:EtOAc = 3:1); ^1^H NMR (500 MHz, CDCl_3_) δ: 6.84 (dt, *J* = 15.5, 6.9 Hz, 1H), 5.74 (dt, *J* = 15.7, 1.6 Hz, 1H), 3.53 (t, *J* = 6.9 Hz, 2H), 2.21–2.17 (m, 2H), 1.83–1.75 (m, 2H), 1.51–1.46 (m, 13H); ^13^C NMR (125 MHz, CDCl_3_) δ: 166.0, 147.3, 123.3, 80.1, 44.8, 32.3, 31.8, 28.1, 27.4, 26.4; IR (neat): 2978, 2935, 2863, 1714, 1653, 1367, 1290, 1256, 1163, 983 cm^−1^; HRMS (ESI-TOF): calcd for C_12_H_21_ClNaO_2_^+^: [M + Na]^+^ = 255.1122, found 255.1114.

- - 1. **Synthetic procedure for Allyl Acetate 7**

**Procedure for the preparation of S7:** Allyl acetate **S7** was synthesized according to a modified literature procedure. ^[52]^ To an oven-dried two-necked 50 mL flask equipped with a magnetic stirring bar, lithium (249.8 mg, 36.0 mmol) and THF (17 mL) were added. The solution was stirred at room temperature for 10 min, and then chloro(dimethyl)phenylsilane (1.23 g, 7.21 mmol) was added dropwise. The reaction mixture was cooled to –78 °C and stirred for 20 min. Crotonaldehyde (421.0 mg, 6.01 mmol) in THF (5 mL) was then added, and the solution was stirred at the same temperature for 1 h before being quenched with a saturated NH_4_Cl aqueous solution. The aqueous layer was extracted with EtOAc (3 × 10 mL), washed with brine (10 mL), dried over anhydrous Na_2_SO_4_, and concentrated in vacuo to afford allylic alcohol **S7** as a colorless oil, which was used without further purification in the next step.

**Procedure for the preparation of 7**: Allyl acetate **7** was synthesized according to a modified literature procedure.^[86]^ An oven-dried two-necked 50 mL flask equipped with a magnetic stirring bar was charged with allylic alcohol **S7**, DMAP (73.3 mg, 0.610 mmol), CH_2_Cl_2_ (24 mL), and Et_3_N (1.67 mL, 12.0 mmol). The solution was cooled to 0 °C, and acetic anhydride (0.85 mL, 9.0 mmol) was added. The reaction mixture was stirred at room temperature for 1 h and then quenched with a saturated NH_4_Cl aqueous solution. The aqueous layer was extracted with EtOAc (3 × 10 mL), washed with brine (10 mL), dried over anhydrous Na_2_SO_4_, and concentrated in vacuo. The crude product was purified by flash column chromatography (hexane:EtOAc = 97:3) to afford allyl acetate **7** as a colorless oil (460.0 mg, 1.85 mmol, 31% yield over two steps).

- - 1. **Characterization of Allyl Acetate 7**

**(*E*)-1-[Dimethyl(phenyl)silyl]but-2-en-1-yl acetate (7):**

Allyl acetate **7** was obtained as a colorless oil (460.0 mg, 1.85 mmol, 31% yield over two steps) after purification by flash column chromatography (hexane:EtOAc = 97:3); ^1^H NMR (500 MHz, CDCl_3_) δ: 7.55–7.48 (m, 2H), 7.42–7.31 (m, 3H), 5.52–5.39 (m, 2H), 5.31 (dd, *J* = 4.8, 2.1 Hz, 1H), 2.02 (s, 3H), 1.65 (dd, *J* = 4.8, 1.0 Hz, 3H), 0.32 (s, 3H), 0.32 (s, 3H); ^13^C NMR (125 MHz, CDCl_3_) δ: 170.7, 135.7, 134.1, 129.4, 127.7, 127.2, 125.2, 69.7, 21.1, 17.8, −5.4, −5.5; IR (neat): 3071, 3025, 2961, 2919, 1740, 1428, 1368, 1235, 1116, 966, 845 cm^−1^; HRMS (ESI-TOF): calcd for C_14_H_20_NaO_2_Si^+^: [M + Na]^+^ = 271.1125, found 271.1118.

- - 1. **Determination of Structure of Allyl Acetate 7**

A similar procedure for preparing **7**, reported in the literature,^[52]^ suggests an alternative structure. Therefore, HMQC and HMBC analyses were conducted, and we determined that the correct structure corresponds to **7**.

- - - 1. **HMQC spectra**


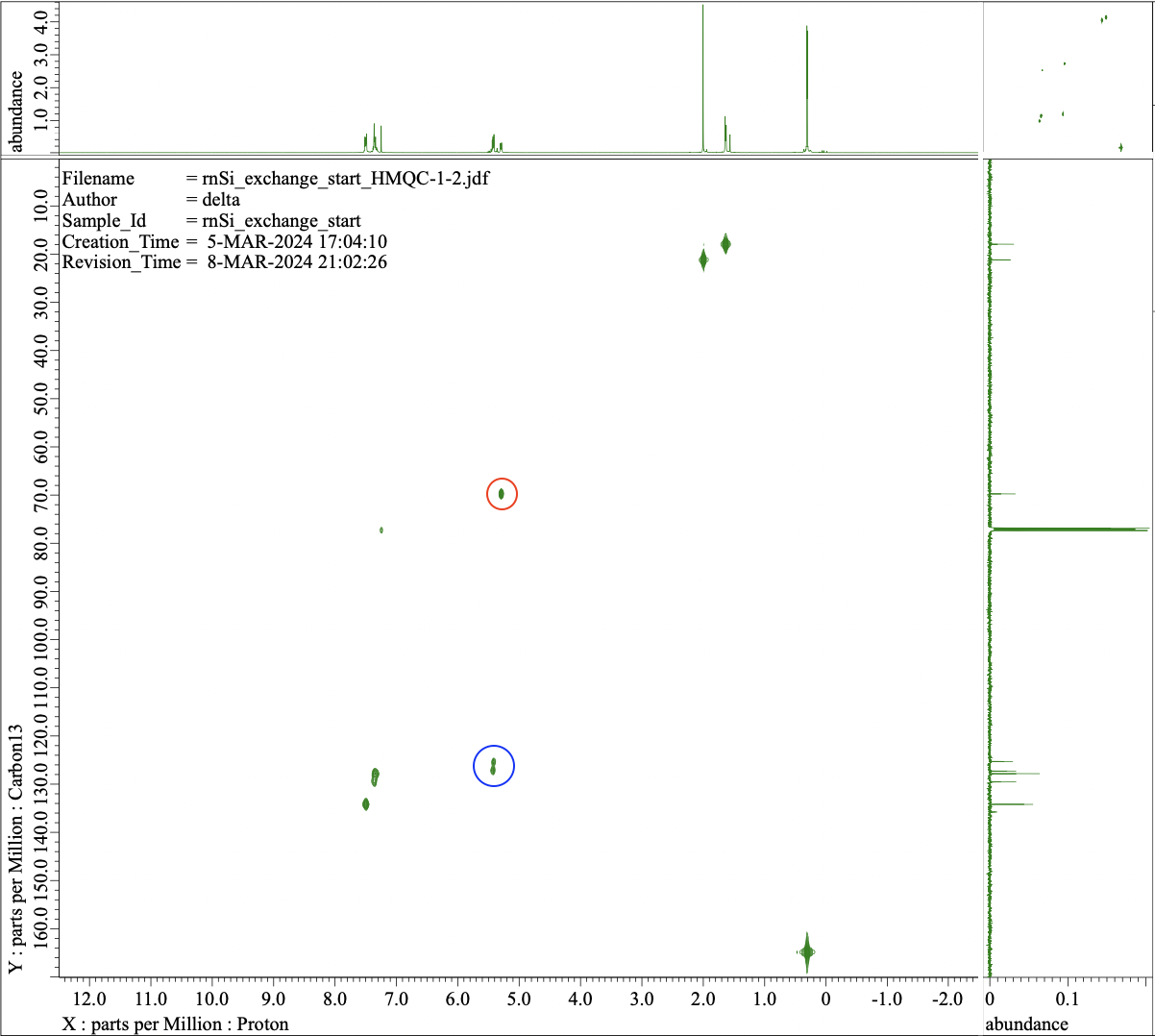


- - - 1. **HMBC spectra**


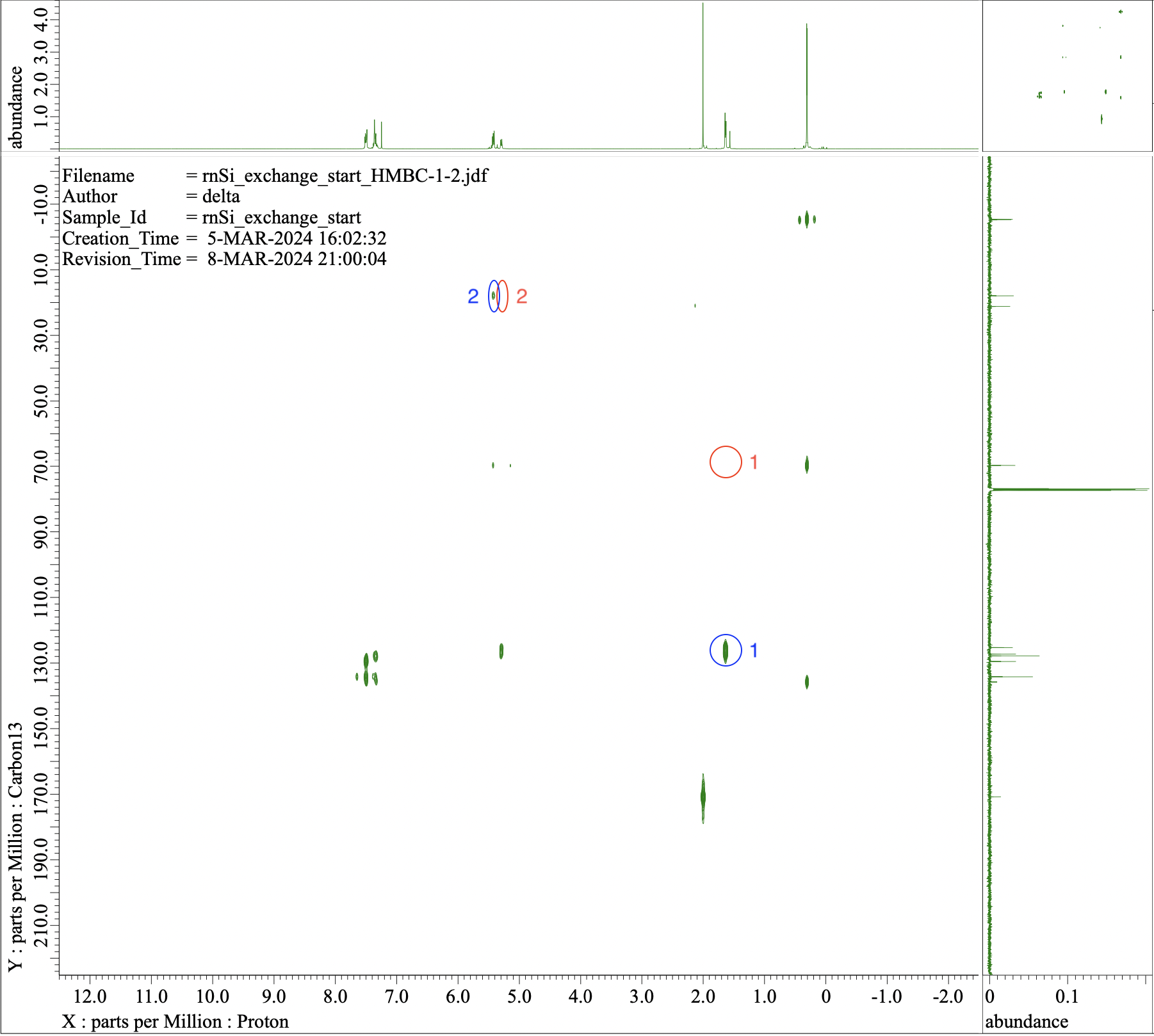


1. **Preparation of *E*/*Z* mixture of Allyl Acetate 2a′**

**Procedure for the preparation of S8**: To an oven-dried round-bottom flask containing a magnetic stirring bar were added 1-phenyl-3-(trimethylsilyl)prop-2-yn-1-ol (921.6 mg, 4.510 mmol) and dissolved in THF (60 mL). The solution was stirred for 10 min at 0 °C. Subsequently, LiAlH_4_ (512.3 mg, 13.50 mmol) was added to the solution. The reaction mixture was allowed to warm to room temperature and stirred for 12 h. The reaction was quenched with a saturated NH_4_Cl aqueous solution (10 mL). The aqueous layer was extracted with EtOAc (30 mL × 3), and the combined organic layers were washed with brine (30 mL), dried over anhydrous Na_2_SO_4_, filtered and concentrated using a rotary evaporator. The residue was purified by column chromatography (hexane:EtOAc = 19:1) to afford allylic alcohol **S8** as a mixture of *E*/*Z* isomers (717.9 mg, 3.48 mmol, 77%, *E*:*Z* = 61:39).

**Procedure for the preparation of 2a′**: Allyl acetate **2a′** was synthesized according to a modified literature procedure.^[86]^ An oven-dried two-necked 50 mL flask equipped with a magnetic stirring bar was charged with allylic alcohol **S8** (717.9 mg, 3.48 mmol), DMAP (46.4 mg, 0.380 mmol), CH_2_Cl_2_ (15 mL), and Et_3_N (685.9 mg, 6.78 mmol). The solution was cooled to 0 °C, and acetic anhydride (541.3 mg, 5.30 mmol) was added. The reaction mixture was stirred at room temperature for 1 h and then quenched with a saturated NH_4_Cl aqueous solution. The aqueous layer was extracted with EtOAc (3 × 10 mL), washed with brine (10 mL), dried over anhydrous Na_2_SO_4_, and concentrated in vacuo. The crude product was purified by flash column chromatography (hexane:EtOAc = 20:1) to afford allyl acetate **2a** as a colorless oil (143.2 mg, 0.576 mmol, 17%, *E*:*Z* = 78:22).

1. **Characterization of Allyl Acetate 2a′**

**1-Phenyl-3-(trimethylsilyl)allyl acetate (2a′):**

Allyl acetate **2a′** was obtained as a colorless oil (143.2 mg, 0.576 mmol, 17%, *E*:*Z* = 78:22) after purification by flash column chromatography (hexane:EtOAc = 20:1); (*Z*)-**2a′** (minor): ^1^H NMR (500 MHz, CDCl_3_) δ: 7.44–7.32 (m, 5H), 6.55–6.44 (m, 2H), 5.87 (dd, *J* = 13.2, 6.6 Hz, 1H), 2.15 (s, 3H), 0.25 (s, 9H); ^13^C NMR (125 MHz, CDCl_3_) δ: 169.8, 143.8, 139.6, 133.8, 128.5, 127.9, 126.6, 75.4, 21.3, 0.0. (*E*)-**2a′** (major): ^1^H NMR (500 MHz, CDCl_3_) δ: 7.44–7.32 (m, 5H), 6.30 (dd, *J* = 5.2, 1.1 Hz, 1H), 6.18 (dd, *J* = 18.9, 5.2 Hz, 1H), 5.96 (dd, *J* = 18.6, 1.4 Hz, 1H), 2.17 (s, 3H), 0.12 (s, 9H); ^13^C NMR (125 MHz, CDCl_3_) δ: 169.9, 142.7, 138.9, 131.8, 128.4, 128.0, 127.2, 77.5, 21.2, −1.5; IR (neat): 3066, 3034, 3000, 2955, 2900, 1743, 1370, 1020, 989, 842, 699 cm^−1^; HRMS (ESI-TOF): calcd for C_14_H_20_NaO_2_Si^+^: [M + Na]^+^ = 271.1125, found 271.1128.


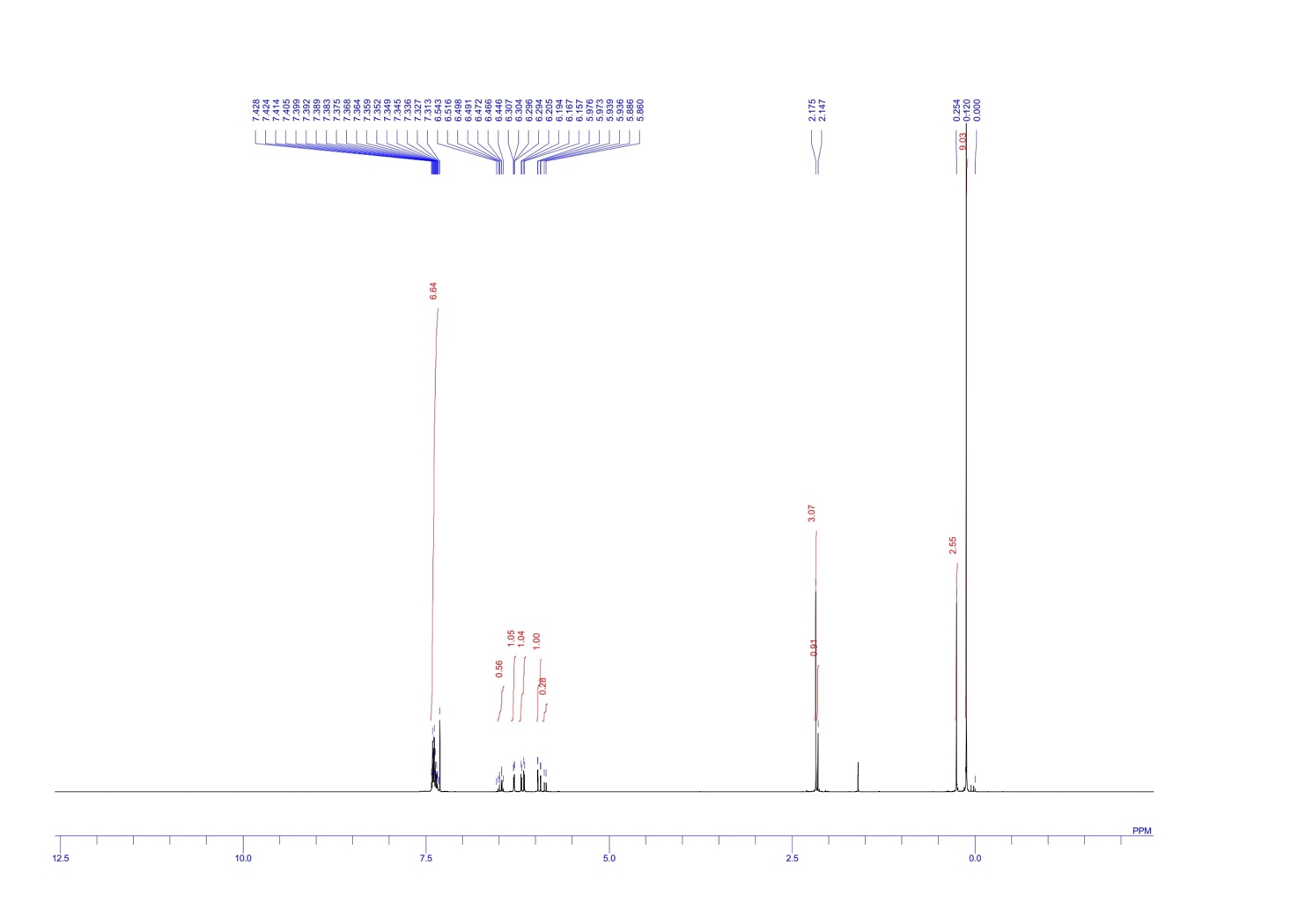


1. **Determination of Relative and Absolute Configurations of 4-Pentanoate 4**

The relative configuration of **4** was determined by comparing the ^1^H NMR spectra of the isolated product **4** with the reported data.^[91]^ The absolute configuration of the reported product **4** was (2*R*,3*S*), with an optical rotation of [α]_D_ = −52.7 (*c* = 0.2, CHCl_3_, for 95% ee). The optical rotation of **4**, synthesized using our protocol, was [α]^22^_D_ = −50.2 (*c* = 0.1, CHCl_3_, for 99% ee), indicating that the absolute configuration of our product is (2*R*,3*S*). The absolute configurations of **3**, **5**, **6**, and **9** were tentatively assigned by analogy to **4**.

- 1. **Reported Data of 4-Pentanoate 4**

**(2*R,*3*S*)-Methyl-2-methyl-3-phenylpent-4-enoate (4):**^[91]^

^1^H NMR (500 MHz, CDCl_3_) δ: 7.33–7.16 (m, 5H), 6.00 (m, 1H), 5.02 (m, 2H), 3.68 (s, 3H), 3.44 (m, 1H), 2.83 (m, 1H), 0.97 (d, *J* = 6.9 Hz, 3H); [α]_D_ = −52.7 (*c* = 0.2, CHCl_3_, for 95% ee).

- 1. **Data of 4-Pentanoate 4 Synthesized by Our Protocol**

**(2*R*,3*S*)-Methyl-2-methyl-3-phenylpent-4-enoate (4):**

^1^H NMR (500 MHz, CDCl_3_) δ: 7.33–7.17 (m, 5H), 6.05–5.85 (m, 1H), 5.17–4.96 (m, 2H), 3.68 (s, 3H), 3.54–3.38 (m, 1H), 2.88–2.78 (m, 1H), 0.97 (d, *J* = 6.9 Hz, 3H); [α]^21^_D_ = −50.2 (*c* = 0.1, CHCl_3_, for 99% ee).

1. **Catalytic Regio- and Enantioselective Hydroallylation of Acrylates 1 using Allyl Acetates 2**
   1. **General Procedure**

To an oven-dried test tube containing Cu(OAc)_2_ (1.8 mg, 0.010 mmol) and (*R*)-tol-BINAP (7.5 mg, 0.011 mmol) in dry DME (0.6 mL) was added HBpin (38.4 mg, 0.300 mmol) at room temperature, and the mixture was stirred for 15 min at the same temperature (Solution A). In a separate oven-dried test tube, Pd(dba)_2_ (6.9 mg, 0.012 mmol), RuPhos (6.2 mg, 0.013 mmol), and dry DME (0.4 mL) were combined and stirred at 50 °C for 15 min (Solution B). Solution B (0.2 mL), acrylate **1** (0.200 mmol), and allyl acetate **2** (0.240 mmol) were then added to Solution A via syringe at 40 °C. After 18 h, the solution was filtered through a plug of Florisil^®^, washed with EtOAc, and the filtrate was concentrated under reduced pressure. The residue was purified by preparative thin-layer chromatography, yielding the desired hydroallylation product **3**.

- 1. **Experimental Procedure for 1 mmol Scale Reaction**

To an oven-dried test tube containing Cu(OAc)_2_ (9.1 mg, 0.050 mmol) and (*R*)-tol-BINAP (37.4 mg, 0.055 mmol) in dry DME (3.0 mL) was added HBpin (192.9 mg, 1.507 mmol) at room temperature, and the mixture was stirred for 15 min at the same temperature (Solution A). In a separate oven-dried test tube, Pd(dba)_2_ (34.5 mg, 0.060 mmol), RuPhos (30.9 mg, 0.066 mmol), and dry DME (2.0 mL) were combined and stirred at 50 °C for 15 min (Solution B). Solution B (1.0 mL), acrylate **1a** (126.8 mg, 0.989 mmol), and allyl acetate **2a** (291.9 mg, 1.173 mmol) were then added to Solution A via syringe at 40 °C. After 18 h, the solution was filtered through a plug of Florisil^®^, washed with EtOAc, and the filtrate was concentrated under reduced pressure. The residue was purified by flash column chromatography (hexane:EtOAc = 50:1), yielding the desired hydroallylation product **3a** (217.3 mg, 0.682 mmol, 69%, dr 90:10, 99% ee).

- 1. **Experimental Procedure for 5 mmol Scale Reaction**

To an oven-dried test tube containing Cu(OAc)_2_ (45.3 mg, 0.249 mmol) and (*R*)-tol-BINAP (186.7 mg, 0.275 mmol) in dry DME (15 mL) was added HBpin (970.9 mg, 7.586 mmol) at room temperature, and the mixture was stirred for 15 min at the same temperature (Solution A). In a separate oven-dried test tube, Pd(dba)_2_ (103.7 mg, 0.180 mmol), RuPhos (92.3 mg, 0.198 mmol), and dry DME (6.0 mL) were combined and stirred at 50 °C for 15 min (Solution B). Solution B (5.0 mL), acrylate **1a** (642.4 mg, 5.012 mmol), and allyl acetate **2a** (1496 mg, 6.013 mmol) were then added to Solution A via syringe at 40 °C. After 18 h, the solution was filtered through a plug of Florisil, washed with EtOAc, and the filtrate was concentrated under reduced pressure. The residue was purified by flash column chromatography (hexane/EtOAc = 100:1), yielding the desired hydroallylation product **3a** (1.279 g, 4.016 mmol, 80%, dr 90:10, 98% ee)

- 1. **Characterization of 4-Pentenoate 3**

***tert*-Butyl (*E*)-2-methyl-3-phenyl-5-(trimethylsilyl)pent-4-enoate (3a):**

The title compound was obtained as a colorless oil in a mixture of diastereomers (59.4 mg, 93%, dr 90:10, 99% ee) after purification by preparative TLC (hexane:EtOAc = 50:1). The enantioselectivity was determined by HPLC analysis after the reduction of **3a** with LiAlH_4_ in THF.

*anti*-**3a** diastereomer (minor): ^1^H NMR (500 MHz, CDCl_3_) δ: 7.43–7.37 (m, 2H), 7.34–7.29 (m, 1H), 7.28–7.24 (m, 2H), 6.12 (dd, *J* = 18.3, 7.4 Hz, 1H), 5.87–5.78 (m, 1H), 3.58–3.46 (m, 1H), 2.85–2.75 (m, 1H), 1.28 (d, *J* = 7.4 Hz, 3H), 1.25 (s, 9H), 0.14 (s, 9H); ^13^C NMR (125 MHz, CDCl_3_) δ: 174.6, 146.4, 142.4, 132.2, 128.2, 128.1, 126.3, 79.8, 57.0, 45.4, 27.6, 15.8, 1.0; (2*R,*3*R*)-*syn*-**3a** diastereomer (major): ^1^H NMR (500 MHz, CDCl_3_) δ: 7.43–7.37 (m, 2H), 7.34–7.29 (m, 1H), 7.28–7.24 (m, 2H), 6.27 (dd, *J* = 18.3, 7.4 Hz, 1H), 5.87–5.78 (m, 1H), 3.58–3.46 (m, 1H), 2.85–2.75 (m, 1H), 1.55 (s, 9H), 1.00 (d, *J* = 7.4 Hz, 3H), 0.12 (s, 9H); ^13^C NMR (125 MHz, CDCl_3_) δ: 175.0, 147.2, 141.6, 130.5, 128.5, 128.3, 126.4, 80.1, 55.9, 45.7, 28.1, 16.2, −1.2; IR (neat): 3002, 2976, 2952, 2896, 1726, 1368, 1248, 1150, 865, 843, 755 cm^−1^; HRMS (ESI-TOF): calcd for C_19_H_30_NaO_2_Si^+^: [M + Na]^+^ = 341.1907, found 341.1913; HPLC (chiral column: CHIRALPAK OJ-H; solvent: hexane:*i*-PrOH = 99:1; flow rate: 1.0 mL/min; detection: at 210 nm; rt): *t*_R_ = 6.0 min (minor) and 6.7 min (major).

***tert*-Butyl (*E*)-2-methyl-3-(*p*-tolyl)-5-(trimethylsilyl)pent-4-enoate (3b):**

The title compound was obtained as a colorless oil in a mixture of diastereomers (56.5 mg, 85%, dr 89:11, >99% ee) after purification by preparative TLC (hexane:EtOAc = 50:1). The enantioselectivity was determined by HPLC analysis after the reduction of **3b** with LiAlH_4_ in THF.

*anti*-**3b** diastereomer (minor): ^1^H NMR (500 MHz, CDCl_3_) δ: 7.22 (d, *J* = 7.4 Hz, 2H), 7.15 (d, *J* = 8.0 Hz, 2H), 6.12 (dd, *J* = 18.3, 8.6 Hz, 1H), 5.85–5.77 (m, 1H), 3.53–3.44 (m, 1H), 2.83–2.72 (m, 1H), 2.41 (s, 3H), 1.29–1.25 (m, 12H), 0.14 (s, 9H); ^13^C NMR (125 MHz, CDCl_3_) δ: 174.6, 146.5, 139.4, 135.8, 131.9, 128.9, 127.9, 79.8, 56.4, 45.4, 27.7, 21.0, 15.7, 1.0; (2*R,*3*R*)-*syn*-**3b** diastereomer (major): ^1^H NMR (500 MHz, CDCl_3_) δ: 7.22 (d, *J* = 7.4 Hz, 2H), 7.15 (d, *J* = 8.0 Hz, 2H), 6.26 (dd, *J* = 18.3, 7.4 Hz, 1H), 5.85–5.77 (m, 1H), 3.53–3.44 (m, 1H), 2.83–2.72 (m, 1H), 2.43 (s, 3H), 1.56 (s, 9H), 1.01 (d, *J* = 6.9 Hz, 3H), 0.12 (s, 9H); ^13^C NMR (125 MHz, CDCl_3_) δ: 175.1, 147.5, 138.6, 135.9, 130.1, 129.2, 128.2, 80.0, 55.5, 45.7, 28.1, 21.0, 16.2, −1.2; IR (neat): 2978, 2957, 2934, 1732, 1514, 1456, 1367, 1248, 1149, 841 cm^−1^; HRMS (ESI-TOF): calcd for C_20_H_32_NaO_2_Si^+^: [M + Na]^+^ = 355.2064, found 355.2061; HPLC (chiral column: CHIRALPAK OD-H; solvent: hexane:*i*-PrOH = 99:1; flow rate: 0.5 mL/min; detection: at 210 nm; rt): *t*_R_ = 12.8 min (minor) and 14.5 min (major).

***tert*-Butyl (*E*)-2-methyl-3-(*m*-tolyl)-5-(trimethylsilyl)pent-4-enoate (3c):**

The title compound was obtained as a colorless oil in a mixture of diastereomers (56.1 mg, 85%, dr 89:11, 88% ee) after purification by preparative TLC (hexane:EtOAc = 50:1). The enantioselectivity was determined by HPLC analysis after the reduction of **3c** with LiAlH_4_ in THF.

*anti*-**3c** diastereomer (minor): ^1^H NMR (500 MHz, CDCl_3_) δ: 7.20–7.13 (m, 1H), 7.03–6.93 (m, 3H), 6.00 (dd, *J* = 18.3, 8.6 Hz, 1H), 5.75–5.66 (m, 1H), 3.42–3.30 (m, 1H), 2.71–2.63 (m, 1H), 2.31 (s, 3H), 1.17–1.13 (m, 12H), 0.03 (s, 9H); ^13^C NMR (125 MHz, CDCl_3_) δ: 174.6, 146.5, 142.3, 137.6, 132.1, 129.0, 128.1, 127.0, 125.0, 79.8, 57.0, 45.4, 27.6, 21.4, 15.8, 1.0; (2*R,*3*R*)-*syn*-**3c** diastereomer (major): ^1^H NMR (500 MHz, CDCl_3_) δ: 7.20–7.13 (m, 1H), 7.03–6.93 (m, 3H), 6.15 (dd, *J* = 18.9, 7.4 Hz, 1H), 5.75–5.66 (m, 1H), 3.42–3.30 (m, 1H), 2.71–2.63 (m, 1H), 2.32 (s, 3H), 1.44 (s, 9H), 0.89 (d, *J* = 6.9 Hz, 3H), 0.01 (s, 9H); ^13^C NMR (125 MHz, CDCl_3_) δ: 175.1, 147.4, 141.6, 138.0, 130.3, 129.1, 128.3, 127.2, 125.2, 80.1, 56.0, 45.7, 28.1, 21.4, 16.2, −1.2; IR (neat): 2977, 2955, 2934, 1732, 1605, 1456, 1367, 1249, 1149, 865, 840 cm^−1^; HRMS (ESI-TOF): calcd for C_20_H_32_NaO_2_Si^+^: [M + Na]^+^ = 355.2064, found 355.2071; HPLC (chiral column: CHIRALPAK OD-H; solvent: hexane:*i*-PrOH = 499:1; flow rate: 0.7 mL/min; detection: at 210 nm; rt): *t*_R_ = 23.5 min (major) and 27.3 min (minor).

***tert*-Butyl (2*R*,3*R,E*)-2-methyl-3-(*o*-tolyl)-5-(trimethylsilyl)pent-4-enoate (3d):**

The title compound was obtained as a colorless oil a colorless oil (45.5 mg, 69%, dr >95:5, 97% ee) after purification by preparative TLC (hexane:EtOAc = 50:1). The enantioselectivity was determined by HPLC analysis after the reduction of **3d** with LiAlH_4_ in THF.

^1^H NMR (500 MHz, CDCl_3_) δ: 7.21–7.07 (m, 4H), 6.07 (dd, *J* = 18.6, 7.2 Hz, 1H), 5.74–5.63 (m, 1H), 3.78 (dd, *J* = 10.9, 7.4 Hz, 1H), 2.81–2.71 (m, 1H), 2.35 (s, 3H), 1.47 (s, 9H), 0.91 (d, *J* = 6.9 Hz, 3H), 0.03–0.00 (m, 9H); ^13^C NMR (125 MHz, CDCl_3_) δ: 175.4, 147.0, 139.7, 136.6, 130.4, 130.3, 127.0, 126.2, 126.0, 80.2, 50.8, 45.5, 28.1, 20.0, 16.0, −1.2; IR (neat): 2977, 2955, 2935, 1732, 1458, 1367, 1249, 1149, 987, 841, 752 cm^−1^; [α]^23^_D_ = −22.4 (*c* = 0.2, CHCl_3_, for 97% ee); HRMS (ESI-TOF): calcd for C_20_H_32_NaO_2_Si^+^: [M + Na]^+^ = 355.2064, found 355.2063; HPLC (chiral column: CHIRALPAK OD-H; solvent: hexane:*i*-PrOH = 99:1; flow rate: 1.0 mL/min; detection: at 210 nm; rt): *t*_R_ = 8.1 min (minor) and 9.0 min (major).

***tert*-Butyl (*E*)-3-(4-fluorophenyl)-2-methyl-5-(trimethylsilyl)pent-4-enoate (3e):**

The title compound was obtained as a colorless oil in a mixture of diastereomers (51.8 mg, 76%, dr 85:15, 91% ee) after purification by preparative TLC (hexane:EtOAc = 19:1). The enantioselectivity was determined by HPLC analysis after the reduction of **3e** with DIBAL in THF.

*anti*-**3e** diastereomer (minor): ^1^H NMR (500 MHz, CDCl_3_) δ: 7.28–7.19 (m, 2H), 7.12–7.06 (m, 2H), 6.07 (dd, *J* = 18.3, 8.0 Hz, 1H), 5.75–5.65 (m, 1H), 3.56–3.44 (m, 1H), 2.80–2.71 (m, 1H), 1.29–1.26 (m, 12H), 0.14 (s, 9H); ^13^C NMR (125 MHz, CDCl_3_) δ: 174.5, 161.5 (d, ^1^*J*_C–F_ = 244.7 Hz), 146.2, 138.2 (d, ^4^*J*_C–F_ = 3.6 Hz), 132.4, 129.5 (d, ^3^*J*_C–F_ = 7.2 Hz), 115.0 (d, ^2^*J*_C–F_ = 20.4 Hz), 80.0, 56.0, 45.5, 27.6, 15.9, 1.0; ^19^F NMR (470 MHz, CDCl_3_) δ: −117.6; (2*R,*3*R*)-*syn*-**3e** diastereomer (major): ^1^H NMR (500 MHz, CDCl_3_) δ: 7.28–7.19 (m, 2H), 7.12–7.06 (m, 2H), 6.23 (dd, *J* = 18.6, 7.2 Hz, 1H), 5.75–5.65 (m, 1H), 3.56–3.44 (m, 1H), 2.80–2.71 (m, 1H), 1.54 (s, 9H), 1.00 (d, *J* = 6.9 Hz, 3H), 0.12 (s, 9H); ^13^C NMR (125 MHz, CDCl_3_) δ: 174.8, 161.5 (d, *J* = 244.7 Hz), 147.0, 137.3 (d, *J* = 3.6 Hz), 130.7, 129.6 (d, *J* = 7.2 Hz), 115.3 (d, *J* = 20.4 Hz), 80.2, 54.9, 45.7, 28.1, 16.1, −1.3; ^19^F NMR (471 MHz, CDCl_3_) δ: −117.3; IR (neat): 2978, 2957, 2936, 1731, 1614, 1510, 1457, 1368, 1249, 1149, 840 cm^−1^; HRMS (ESI-TOF): calcd for C_19_H_29_FNaO_2_Si^+^: [M + Na]^+^ = 359.1813, found 359.1813; HPLC (chiral column: CHIRALPAK AD-H; solvent: hexane:*i*-PrOH = 99:1; flow rate: 0.5 mL/min; detection: at 210 nm; rt): *t*_R_ = 27.2 min (minor) and 30.5 min (major).

***tert*-Butyl (*E*)-3-(4-chlorophenyl)-2-methyl-5-(trimethylsilyl)pent-4-enoate (3f):**

The title compound was obtained as a colorless oil in a mixture of diastereomers (43.4 mg, 62%, dr 88:12, 99% ee) after purification by preparative TLC (hexane:EtOAc = 19:1). The enantioselectivity was determined by HPLC analysis after the reduction of **3f** with DIBAL in THF.

*anti*-**3f** diastereomer (minor): ^1^H NMR (500 MHz, CDCl_3_) δ: 7.41–7.33 (m, 2H), 7.24 (dd, *J* = 6.6, 2.0 Hz, 2H), 6.06 (dd, *J* = 18.3, 8.0 Hz, 1H), 5.86–5.76 (m, 1H), 3.56–3.44 (m, 1H), 2.80–2.72 (m, 1H), 1.28–1.27 (m, 12H), 0.14 (s, 9H); ^13^C NMR (125 MHz, CDCl_3_) δ: 174.4, 145.8, 141.0, 132.2, 132.0, 129.4, 128.3, 80.1, 56.1, 45.3, 27.7, 15.9, −1.1; (2*R,*3*R*)-*syn*-**3f** diastereomer (major): ^1^H NMR (500 MHz, CDCl_3_) δ: 7.41–7.33 (m, 2H), 7.19 (dd, *J* = 6.3, 1.7 Hz, 2H), 6.22 (dd, *J* = 18.6, 7.2 Hz, 1H), 5.89–5.76 (m, 1H), 3.56–3.44 (m, 1H), 2.80–2.72 (m, 1H), 1.55 (s, 9H), 1.00 (d, *J* = 6.9 Hz, 3H), 0.12 (s, 9H); ^13^C NMR (125 MHz, CDCl_3_) δ: 174.7, 146.6, 140.1, 132.8, 131.1, 129.6, 128.7, 80.3, 55.2, 45.5, 28.1, 16.1, −1.3; IR (neat): 2978, 2954, 2934, 1732, 1492, 1457, 1368, 1249, 1150, 1092, 1015, 841 cm^−1^; HRMS (ESI-TOF): calcd for C_19_H_29_ClNaO_2_Si^+^: [M + Na]^+^ = 375.1518, found 375.1513; HPLC (chiral column: CHIRALPAK AD-H; solvent: hexane:*i*-PrOH = 199:1; flow rate: 1.0 mL/min; detection: at 210 nm; rt): *t*_R_ = 15.8 min (minor) and 18.4 min (major).

***tert*-Butyl (*E*)-3-(4-methoxyphenyl)-2-methyl-5-(trimethylsilyl)pent-4-enoate (3g):**

The title compound was obtained as a colorless solid in a mixture of diastereomers (58.8 mg, 85%, dr 89:11, 99% ee) after purification by preparative TLC (hexane:EtOAc = 50:1). The enantioselectivity was determined by HPLC analysis after the reduction of **3g** with LiAlH_4_ in THF.

mp: 49.2–50.0 °C; *anti*-**3g** diastereomer (minor): ^1^H NMR (500 MHz, CDCl_3_) δ: 7.13–7.10 (m, 2H), 6.86–6.80 (m, 2H), 5.99 (dd, *J* = 18.3, 8.0 Hz, 1H), 5.72–5.63 (m, 1H), 3.80–3.76 (m, 3H), 3.41–3.31 (m, 1H), 2.70–2.59 (m, 1H), 1.19–1.13 (m, 12H), 0.04–−0.01 (m, 9H); (2*R,*3*R*)-*syn*-**3g** diastereomer (major): ^1^H NMR (500 MHz, CDCl_3_) δ: 7.09–7.04 (m, 2H), 6.89–6.80 (m, 2H), 6.14 (dd, *J* = 18.3, 7.4 Hz, 1H), 5.72–5.63 (m, 1H), 3.80–3.76 (m, 3H), 3.41–3.31 (m, 1H), 2.70–2.59 (m, 1H), 1.44 (s, 9H), 0.89 (d, *J* = 6.9 Hz, 3H), 0.04–−0.01 (m, 9H); ^13^C NMR (125 MHz, CDCl_3_) δ: 175.1, 158.1, 147.6, 133.6, 130.0, 129.2, 113.8, 80.1, 55.2, 55.0, 45.8, 28.1, 16.2, −1.2; IR (neat): 2978, 2956, 2835, 1731, 1614, 1513, 1457, 1367, 1254, 1147, 840 cm^−1^; HRMS (ESI-TOF): calcd for C_20_H_32_NaO_3_Si^+^: [M + Na]^+^ = 371.2013, found 371.2013; HPLC (chiral column: CHIRALPAK OD-H; solvent: hexane:*i*-PrOH = 99:1; flow rate: 1.0 mL/min; detection: at 210 nm; rt): *t*_R_ = 11.6 min (major) and 13.9 min (minor).

***tert*-Butyl (2*R*,3*R*,*E*)-2-methyl-3-(naphthalen-1-yl)-5-(trimethylsilyl)pent-4-enoate (3h):**

The title compound was obtained as a colorless oil (48.1 mg, 65%, dr >95:5, 99% ee) after purification by preparative TLC (hexane:EtOAc = 50:1). The enantioselectivity was determined by HPLC analysis after the reduction of **3h** with LiAlH_4_ in THF.

^1^H NMR (500 MHz, CDCl_3_) δ: 8.44 (d, *J* = 8.0 Hz, 1H), 8.09 (d, *J* = 7.4 Hz, 1H), 7.97 (d, *J* = 8.0 Hz, 1H), 7.77–7.67 (m, 3H), 7.58 (d, *J* = 7.4 Hz, 1H), 6.50 (dd, *J* = 18.6, 7.2 Hz, 1H), 6.08–5.99 (m, 1H), 4.61 (dd, *J* = 10.9, 7.4 Hz, 1H), 3.24–3.16 (m, 1H), 1.72 (s, 9H), 1.12 (d, *J* = 6.9 Hz, 3H), 0.23 (s, 9H); ^13^C NMR (125 MHz, CDCl_3_) δ: 175.4, 147.1, 137.9, 134.0, 132.3, 130.7, 128.9, 126.9, 125.8, 125.5, 125.4, 125.1, 123.6, 80.3, 49.8, 45.9, 28.2, 16.3, −1.2; IR (neat): 3046, 2977, 2956, 1728, 1456, 1367, 1248, 1150, 864, 841, 779 cm^−1^; [α]^23^_D_ = +80.2 (*c* = 0.3, CHCl_3_, for 99% ee); HRMS (ESI-TOF): calcd for C_23_H_32_NaO_2_Si^+^: [M + Na]^+^ = 391.2064, found 391.2074; HPLC (chiral column: CHIRALPAK OD-H; solvent: hexane:*i*-PrOH = 49:1; flow rate: 0.5 mL/min; detection: at 210 nm; rt): *t*_R_ = 17.7 min (major) and 20.7 min (minor).

***tert*-Butyl (*E*)-2-methyl-3-(naphthalen-2-yl)-5-(trimethylsilyl)pent-4-enoate (3i):**

The title compound was obtained as a colorless solid in a mixture of diastereomers (54.4 mg, 73%, dr 86:14, 99% ee) after purification by preparative TLC (hexane:EtOAc = 50:1). The enantioselectivity was determined by HPLC analysis after the reduction of **3i** with LiAlH_4_ in THF.

mp: 80.2–81.1 °C; *anti*-**3i** diastereomer (minor): ^1^H NMR (500 MHz, CDCl_3_) δ: 7.90–7.76 (m, 3H), 7.70–7.62 (m, 1H), 7.55–7.38 (m, 2H), 7.34 (dd, *J* = 8.4, 1.5 Hz, 1H), 6.14 (dd, *J* = 18.4, 8.4 Hz, 1H), 5.88–5.73 (m, 1H), 3.75–3.53 (m, 1H), 2.95–2.73 (m, 1H), 1.26 (d, *J* = 6.9 Hz, 3H), 1.10 (s, 9H), 0.07 (s, 9H); ^13^C NMR (125 MHz, CDCl_3_) δ: 174.6, 146.2, 139.9, 133.4, 132.6, 131.1, 130.6, 127.8, 127.5, 126.6, 126.4, 125.8, 125.3, 79.9, 56.9, 45.2, 27.6, 15.8, 1.0; (2*R,*3*R*)-*syn*-**3i** diastereomer (major): ^1^H NMR (500 MHz, CDCl_3_) δ: 7.90–7.76 (m, 3H), 7.70–7.62 (m, 1H), 7.55–7.38 (m, 2H), 7.34 (dd, *J* = 8.4, 1.5 Hz, 1H), 6.28 (dd, *J* = 18.6, 7.2 Hz, 1H), 5.88–5.73 (m, 1H), 3.75–3.53 (m, 1H), 2.95–2.73 (m, 1H), 1.50 (s, 9H), 0.96 (d, *J* = 6.9 Hz, 3H), 0.05 (s, 9H); ^13^C NMR (125 MHz, CDCl_3_) δ: 175.0, 147.1, 139.1, 133.5, 132.3, 130.8, 128.2, 127.6, 127.6, 127.1, 126.2, 126.0, 125.4, 80.2, 56.1, 45.5, 28.1, 16.3, −1.2; IR (neat): 3060, 2977, 2953, 2936, 1721, 1368, 1247, 1150, 866, 835, 757 cm^−1^; HRMS (ESI-TOF): calcd for C_23_H_32_NaO_2_Si^+^: [M + Na]^+^ = 391.2064, found 391.2051; HPLC (chiral column: CHIRALPAK OD-H; solvent: hexane:*i*-PrOH = 49:1; flow rate: 0.5 mL/min; detection: at 210 nm; rt): *t*_R_ = 16.0 min (major) and 28.8 min (minor).

***tert*-Butyl (*E*)-2-methyl-3-(thiophen-2-yl)-5-(trimethylsilyl)pent-4-enoate (3j):**

The title compound was obtained as a pale yellow oil in a mixture of diastereomers (51.6 mg, 80%, dr 79:21, 90% ee) after purification by preparative TLC (hexane:EtOAc = 19:1). The enantioselectivity was determined by HPLC analysis after the reduction of **3j** with LiAlH_4_ in THF.

*anti*-**3j** diastereomer (minor): ^1^H NMR (500 MHz, CDCl_3_) δ: 7.21–7.12 (m, 1H), 6.97–6.89 (m, 1H), 6.84–6.79 (m, 1H), 6.00 (dd, *J* = 18.3, 8.6 Hz, 1H), 5.81–5.73 (m, 1H), 3.83–3.74 (m, 1H), 2.73–2.62 (m, 1H), 1.29 (s, 9H), 1.15 (d, *J* = 6.9 Hz, 3H), 0.05 (s, 9H); (2*R,*3*R*)-*syn*-**3j** diastereomer (major): ^1^H NMR (500 MHz, CDCl_3_) δ: 7.21–7.12 (m, 1H), 6.97–6.89 (m, 1H), 6.84–6.79 (m, 1H), 6.14 (dd, *J* = 18.3, 7.4 Hz, 1H), 5.81–5.73 (m, 1H), 3.83–3.74 (m, 1H), 2.73–2.62 (m, 1H), 1.43 (s, 9H), 1.02 (d, *J* = 6.9 Hz, 3H), 0.03 (s, 9H); ^13^C NMR (125 MHz, CDCl_3_) δ: 174.4, 146.5, 144.9, 131.1, 126.6, 124.6, 123.8, 80.3, 50.7, 46.6, 28.0, 15.9, −1.3; IR (neat): 2978, 2957, 2933, 1728, 1368, 1249, 1150, 870, 842, 695 cm^−1^; HRMS (ESI-TOF): calcd for C_17_H_28_NaO_2_SSi^+^: [M + Na]^+^ = 347.1471, found 347.1458; HPLC (chiral column: CHIRALPAK OJ-H; solvent: hexane:*i*-PrOH = 199:1; flow rate: 1.0 mL/min; detection: at 210 nm; rt): *t*_R_ = 8.8 min (minor) and 10.5 min (major).

***tert*-Butyl (*E*)-2-methyl-3-phenethyl-5-(trimethylsilyl)pent-4-enoate (3k):**

The title compound was obtained as a colorless oil in a mixture of diastereomers (40.5 mg, 58%, dr 90:10, 99% ee) after purification by preparative TLC (hexane:EtOAc = 19:1). The enantioselectivity was determined by HPLC analysis after the reduction of **3k** with LiAlH_4_ in THF.

*anti*-**3k** diastereomer (minor): ^1^H NMR (500 MHz, CDCl_3_) δ: 7.33–7.29 (m, 2H), 7.22–7.17 (m, 3H), 5.92 (dd, *J* = 18.3, 8.6 Hz, 1H), 5.78–5.70 (m, 1H), 2.70–2.65 (m, 1H), 2.56–2.48 (m, 1H), 2.44–2.37 (m, 1H), 2.35–2.26 (m, 1H), 1.87–1.74 (m, 1H), 1.67–1.56 (m, 1H), 1.46 (s, 9H), 1.07 (d, *J* = 6.3 Hz, 3H), 0.12 (s, 9H); (2*R,*3*R*)-*syn*-**3k** diastereomer (major): ^1^H NMR (500 MHz, CDCl_3_) δ: 7.31 (t, *J* = 7.4 Hz, 2H), 7.20 (t, *J* = 7.2 Hz, 3H), 5.92 (dd, *J* = 18.3, 8.6 Hz, 1H), 5.78–5.70 (m, 1H), 2.70–2.65 (m, 1H), 2.56–2.48 (m, 1H), 2.44–2.37 (m, 1H), 2.35–2.26 (m, 1H), 1.87–1.74 (m, 1H), 1.67–1.56 (m, 1H), 1.46 (s, 9H), 1.11 (d, *J* = 6.9 Hz, 3H), 0.12 (s, 9H); ^13^C NMR (125 MHz, CDCl_3_) δ: 174.7, 147.5, 142.4, 132.4, 128.4, 128.3, 125.7, 79.9, 49.1, 44.4, 33.4, 32.8, 28.1, 14.3, −1.1; IR (neat): 3027, 2977, 2954, 1728, 1456, 1367, 1249, 1152, 867, 840 cm^−1^; HRMS (ESI-TOF): calcd for C_21_H_34_NaO_2_Si^+^: [M + Na]^+^ = 369.2220, found 369.2230; HPLC (chiral column: CHIRALPAK AD-H; solvent: hexane:*i*-PrOH = 199:1; flow rate: 1.0 mL/min; detection: at 210 nm; rt): *t*_R_ = 9.8 min (major) and 10.9 min (minor).

***tert*-Butyl-2-methyl-6-phenoxy-3-[(*E*)-2-(trimethylsilyl)vinyl]hexanoate (3l):**

The title compound was obtained as a colorless oil in a mixture of diastereomers (40.5 mg, 54%, dr 79:21, 98% ee) after purification by preparative TLC (hexane:EtOAc = 19:1). The enantioselectivity was determined by HPLC analysis after the reduction of **3l** with LiAlH_4_ in THF.

*anti*-**3l** diastereomer (minor): ^1^H NMR (500 MHz, CDCl_3_) δ: 7.36–7.31 (m, 2H), 7.01–6.97 (m, 1H), 6.95 (d, *J* = 8.6 Hz, 2H), 5.90 (dd, *J* = 18.6, 8.3 Hz, 1H), 5.78–5.71 (m, 1H), 4.04–3.97 (m, 2H), 2.46–2.40 (m, 1H), 2.37–2.30 (m, 1H), 1.89–1.82 (m, 1H), 1.79–1.69 (m, 3H), 1.51 (s, 9H), 1.10 (d, *J* = 6.3 Hz, 3H), 0.12 (s, 9H); (2*R,*3*R*)-*syn*-**3l** diastereomer (major): ^1^H NMR (500 MHz, CDCl_3_) δ: 7.36–7.31 (m, 2H), 6.99 (t, *J* = 7.4 Hz, 1H), 6.95 (d, *J* = 8.6 Hz, 2H), 5.90 (dd, *J* = 18.6, 8.3 Hz, 1H), 5.78–5.71 (m, 1H), 4.04–3.97 (m, 2H), 2.46–2.40 (m, 1H), 2.37–2.30 (m, 1H), 1.89–1.82 (m, 1H), 1.79–1.69 (m, 3H), 1.49 (s, 9H), 1.15 (d, *J* = 7.4 Hz, 3H), 0.12 (s, 9H); ^13^C NMR (125 MHz, CDCl_3_) δ: 174.7, 159.0, 147.5, 132.2, 129.4, 120.5, 114.4, 79.9, 67.7, 49.4, 44.6, 28.1, 27.4, 27.0, 14.4, −1.1; IR (neat): 2975, 2953, 2875, 1730, 1602, 1498, 1367, 1246, 1153, 867, 840 cm^−1^; HRMS (ESI-TOF): calcd for C_22_H_36_NaO_3_Si^+^: [M + Na]^+^ = 399.2326, found 399.2314; HPLC (chiral column: CHIRALPAK OJ-H; solvent: hexane:*i*-PrOH = 199:1; flow rate: 1.0 mL/min; detection: at 210 nm; rt): *t*_R_ = 22.7 min (minor) and 25.0 min (major).

***tert*-Butyl (2*R*,3*R*,*E*)-2-methyl-3-phenyl-5-(triethylsilyl)pent-4-enoate (3m):**

The title compound was obtained as a colorless oil (52.8 mg, 73%, dr >95:5, 98% ee) after purification by preparative TLC (hexane:EtOAc = 19:1). The enantioselectivity was determined by HPLC analysis after the reduction of **3m** with LiAlH_4_ in THF.

^1^H NMR (500 MHz, CDCl_3_) δ: 7.31–7.27 (m, 2H), 7.22–7.17 (m, 1H), 7.15 (d, *J* = 8.0 Hz, 2H), 6.21 (dd, *J* = 18.9, 6.9 Hz, 1H), 5.69–5.61 (m, 1H), 3.48 (dd, *J* = 10.3, 6.9 Hz, 1H), 2.75–2.66 (m, 1H), 1.44 (s, 9H), 0.94–0.84 (m, 12H), 0.52 (q, *J* = 7.8 Hz, 6H); ^13^C NMR (125 MHz, CDCl_3_) δ: 175.0, 148.7, 141.7, 128.4, 128.4, 126.5, 126.4, 80.1, 55.6, 45.5, 28.0, 16.3, 7.3, 3.4; IR (neat): 3027, 2954, 2910, 2875, 1731, 1457, 1367, 1149, 1017, 722 cm^−1^; [α]^23^_D_ = −22.0 (*c* = 0.3, CHCl_3_, for 97% ee); HRMS (ESI-TOF): calcd for C_22_H_36_NaO_2_Si^+^: [M + Na]^+^ = 383.2377, found 383.2376; HPLC (chiral column: CHIRALPAK OJ-H; solvent: hexane:*i*-PrOH = 199:1; flow rate: 1.0 mL/min; detection: at 210 nm; rt): *t*_R_ = 6.2 min (minor) and 7.8 min (major).

***tert*-Butyl (2*R*,3*R*,*E*)-2-methyl-3-phenyl-5-(triisopropylsilyl)pent-4-enoate (3n):**

The title compound was obtained as a colorless oil (42.5 mg, 53%, dr >95:5, >99% ee) after purification by preparative TLC (hexane:EtOAc = 19:1). The enantioselectivity was determined by HPLC analysis after the reduction of **3n** with LiAlH_4_ in THF.

^1^H NMR (500 MHz, CDCl_3_) δ: 7.31–7.26 (m, 2H), 7.21–7.17 (m, 1H), 7.15 (d, *J* = 7.4 Hz, 2H), 6.26 (dd, *J* = 19.2, 6.6 Hz, 1H), 5.59 (d, *J* = 19.5 Hz, 1H), 3.54 (dd, *J* = 9.7, 6.9 Hz, 1H), 2.76 (dt, *J* = 17.0, 7.0 Hz, 1H), 1.43 (s, 9H), 1.10–0.96 (m, 21H), 0.93 (d, *J* = 6.9 Hz, 3H); ^13^C NMR (125 MHz, CDCl_3_) δ: 175.0, 149.5, 141.8, 128.3, 126.3, 124.4, 80.1, 55.4, 45.2, 28.0, 18.6, 18.5, 16.4, 10.8; IR (neat): 2961, 2941, 2865, 1730, 1462, 1367, 1148, 883, 759, 700 cm^−1^; [α]^23^_D_ = −11.1 (*c* = 0.2, CHCl_3_, for 98% ee); HRMS (ESI-TOF): calcd for C_25_H_42_NaO_2_Si^+^: [M + Na]^+^ = 425.2846, found 425.2830; HPLC (chiral column: CHIRALPAK OJ-H; solvent: hexane:*i*-PrOH = 199:1; flow rate: 1.0 mL/min; detection: at 210 nm; rt): *t*_R_ = 5.4 min (minor) and 6.4 min (major).

***tert*-Butyl (2*R*,3*R*,*E*)-5-(dimethyl(phenyl)silyl)-2-methyl-3-phenylpent-4-enoate (3o):**

The title compound was obtained as a colorless oil (65.6 mg, 86%, dr >95:5, 99% ee) after purification by preparative TLC (hexane:EtOAc = 19:1). The enantioselectivity was determined by HPLC analysis after the reduction of **3o** with LiAlH_4_ in THF.

^1^H NMR (500 MHz, CDCl_3_) δ: 7.48–7.43 (m, 2H), 7.35–7.26 (m, 5H), 7.24–7.18 (m, 1H), 7.15 (d, *J* = 6.9 Hz, 2H), 6.28 (dd, *J* = 18.6, 7.2 Hz, 1H), 5.90–5.80 (m, 1H), 3.53–3.42 (m, 1H), 2.76–2.67 (m, 1H), 1.39 (s, 9H), 0.90 (d, *J* = 6.9 Hz, 3H), 0.29 (s, 6H); ^13^C NMR (125 MHz, CDCl_3_) δ: 175.0, 149.2, 141.4, 138.7, 133.8, 128.8, 128.5, 128.4, 128.2, 127.6, 126.5, 80.2, 55.7, 45.5, 28.0, 16.3, −2.5; IR (neat): 3026, 2977, 2933, 1727, 1456, 1367, 1249, 1148, 1115, 839, 701 cm^−1^; [α]^24^_D_ = −24.4 (*c* = 0.2, CHCl_3_, for 99% ee); HRMS (ESI-TOF): calcd for C_24_H_32_NaO_2_Si^+^: [M + Na]^+^ = 403.2064, found 403.2049; HPLC (chiral column: CHIRALPAK AD-H; solvent: hexane:*i*-PrOH = 199:1; flow rate: 1.0 mL/min; detection: at 210 nm; rt): *t*_R_ = 24.2 min (major) and 26.4 min (minor).

***tert*-Butyl (2*R*,3*R*,*E*)-5-(benzyldimethylsilyl)-2-methyl-3-phenylpent-4-enoate (3p):**

The title compound was obtained as a colorless oil in a mixture of diastereomers (61.6 mg, 78%, dr 91:9, 99% ee) after purification by preparative TLC (hexane:EtOAc = 50:1). The enantioselectivity was determined by HPLC analysis after the reduction of **3p** with LiAlH_4_ in THF.

*anti*-**3p** diastereomer (minor): *anti*-**3p** diastereomer (minor): ^1^H NMR (500 MHz, CDCl_3_) δ: 7.32–7.01 (m, 8H), 6.93–6.89 (m, 2H), 5.96 (dd, *J* = 18.6, 8.4 Hz, 1H), 5.68 (d, *J* = 19.0 Hz, 1H), 3.37 (dd, *J* = 9.5, 9.5 Hz, 1H), 2.72–2.66 (m, 1H), 2.08 (s, 2H), 1.14–1.13 (m, 12H), 0.02 (s, 3H), 0.01 (s, 3H); ^13^C NMR (125 MHz, CDCl_3_) δ: 174.5, 147.9, 142.2, 139.8, 129.9, 128.4, 128.2, 128.1, 128.0, 126.3, 123.8, 79.8, 57.0, 45.2, 27.6, 26.0, 16.0, −3.4, −3.5; (2*R,*3*R*)-*syn*-**3p** diastereomer (major): ^1^H NMR (500 MHz, CDCl_3_) δ: 7.32–7.01 (m, 8H), 6.93–6.89 (m, 2H), 6.15 (dd, *J* = 18.3, 7.4 Hz, 1H), 5.66 (d, *J* = 18.5 Hz, 1H), 3.44 (dd, *J* = 10.5, 7.5 Hz, 1H), 2.72–2.66 (m, 1H), 2.06 (s, 2H), 1.43 (s, 9H), 0.90 (d, *J* = 7.4 Hz, 3H), −0.02 (s, 3H), −0.01 (s, 3H); ^13^C NMR (125 MHz, CDCl_3_) δ: 75.0, 148.6, 141.3, 139.8, 128.5, 128.4, 128.3, 128.2, 128.0, 126.5, 123.8, 80.1, 55.8, 45.4, 28.1, 25.9, 16.2, −3.4, −3.5; IR (neat): 3060, 3026, 2933, 2896, 1728, 1601, 1493, 1453, 1367, 1248, 1148 cm^−1^; HRMS (Dart): calcd for C_25_H_35_O_2_Si^+^: [M + H]^+^ = 395.2401, found 395.2389; HPLC (chiral column: CHIRALPAK OJ-H; solvent: hexane:*i*-PrOH = 99:1; flow rate: 0.7 mL/min; detection: at 254 nm; 60 °C): *t*_R_ = 15.3min (major) and 18.5 min (minor).

**Ethyl (2*R*,3*R*,*E*)-2-methyl-3-phenyl-5-(triethylsilyl)pent-4-enoate (3q):**

The title compound was obtained as a colorless oil (43.8 mg, 68%, dr >95:5, 98% ee) after purification by preparative TLC (hexane:EtOAc = 19:1). The enantioselectivity was determined by HPLC analysis after the reduction of **3q** with LiAlH_4_ in THF.

^1^H NMR (500 MHz, CDCl_3_) δ: 7.32–7.28 (m, 2H), 7.23–7.19 (m, 1H), 7.17 (d, *J* = 7.4 Hz, 2H), 6.18 (dd, *J* = 18.9, 7.4 Hz, 1H), 5.72–5.60 (m, 1H), 4.19–4.03 (m, 2H), 3.49 (dd, *J* = 10.3, 8.0 Hz, 1H), 2.87–2.77 (m, 1H), 1.25 (t, *J* = 7.2 Hz, 3H), 0.95 (d, *J* = 6.9 Hz, 3H), 0.88 (t, *J* = 8.0 Hz, 9H), 0.51 (q, *J* = 7.8 Hz, 6H); ^13^C NMR (125 MHz, CDCl_3_) δ: 175.7, 148.3, 141.4, 128.5, 128.2, 127.0, 126.5, 60.2, 56.3, 45.1, 16.0, 14.2, 7.3, 3.9; IR (neat): 2953, 2936, 2911, 2874, 1737, 1455, 1240, 1160, 1017, 722 cm^−1^; [α]^23^_D_ = −49.5 (*c* = 0.1, CHCl_3_, for 98% ee); HRMS (ESI-TOF): calcd for C_20_H_32_NaO_2_Si^+^: [M + Na]^+^ = 355.2064, found 355.2071; HPLC (chiral column: CHIRALPAK OJ-H; solvent: hexane:*i*-PrOH = 199:1; flow rate: 1.0 mL/min; detection: at 210 nm; rt): *t*_R_ = 6.2 min (minor) and 7.6 min (major).

***tert*-Butyl (2*R*,3*R*,*E*)-2-ethyl-3-phenyl-5-(triethylsilyl)pent-4-enoate (3r):**

The title compound was obtained as a colorless oil (39.2 mg, 53%, dr >95:5, 97% ee) after purification by preparative TLC (hexane:EtOAc = 19:1). The enantioselectivity was determined by HPLC analysis after the reduction of **3r** with LiAlH_4_ in THF.

^1^H NMR (500 MHz, CDCl_3_) δ: 7.31–7.27 (m, 2H), 7.21–7.17 (m, 1H), 7.15 (d, *J* = 6.9 Hz, 2H), 6.19 (dd, *J* = 18.6, 7.2 Hz, 1H), 5.67–5.59 (m, 1H), 3.48 (dd, *J* = 10.3, 7.4 Hz, 1H), 2.55 (td, *J* = 10.6, 4.0 Hz, 1H), 1.45 (s, 9H), 1.40–1.32 (m, 1H), 1.23–1.14 (m, 1H), 0.95–0.85 (m, 9H), 0.80 (t, *J* = 7.4 Hz, 3H), 0.51 (q, *J* = 8.0 Hz, 6H); ^13^C NMR (125 MHz, CDCl_3_) δ: 174.3, 148.6, 142.0, 128.4, 128.3, 126.6, 126.3, 80.2, 55.3, 53.1, 28.2, 24.3, 11.6, 7.3, 3.4; IR (neat): 2956, 2875, 1730, 1456, 1366, 1271, 1150, 1016, 759, 722 cm^−1^; [α]^24^_D_ = −24.6 (*c* = 0.2, CHCl_3_, for 97% ee); HRMS (ESI-TOF): calcd for C_23_H_38_NaO_2_Si^+^: [M + Na]^+^ = 397.2533, found 397.2539; HPLC (chiral column: CHIRALPAK OD-H; solvent: hexane:*i*-PrOH = 199:1; flow rate: 1.0 mL/min; detection: at 210 nm; rt): *t*_R_ = 8.8 min (major) and 9.8 min (minor).

***tert*-Butyl (2*R*,3*R*,*E*)-2-isobutyl-3-phenyl-5-(triethylsilyl)pent-4-enoate (3s):**

The title compound was obtained as a colorless oil (31.3 mg, 45%, dr >95:5, 99% ee) after purification by preparative TLC (hexane:EtOAc = 19:1). The enantioselectivity was determined by HPLC analysis after the reduction of **3s** with LiAlH_4_ in THF.

^1^H NMR (500 MHz, CDCl_3_) δ: 7.31–7.26 (m, 2H), 7.22–7.17 (m, 1H), 7.14 (d, *J* = 7.4 Hz, 2H), 6.19 (dd, *J* = 18.9, 7.4 Hz, 1H), 5.67–5.58 (m, 1H), 3.44 (dd, *J* = 10.3, 7.4 Hz, 1H), 2.73 (td, *J* = 10.7, 3.8 Hz, 1H), 1.52–1.39 (m, 11H), 0.92–0.82 (m, 10H), 0.80 (d, *J* = 6.3 Hz, 3H), 0.78 (d, *J* = 6.3 Hz, 3H), 0.51 (q, *J* = 8.0 Hz, 6H); ^13^C NMR (125 MHz, CDCl_3_) δ: 174.4, 148.6, 142.0, 128.4, 128.2, 126.8, 126.3, 80.2, 56.0, 49.4, 40.4, 28.1, 26.0, 23.8, 21.1, 7.3, 3.4; IR (neat): 2955, 2935, 2913, 2874, 1730, 1456, 1367, 1250, 1148, 1016, 722 cm^−1^; [α]^24^_D_ = −5.0 (*c* = 0.2, CHCl_3_, for 99% ee); HRMS (ESI-TOF): calcd for C_25_H_42_NaO_2_Si^+^: [M + Na]^+^ = 425.2846, found 425.2852; HPLC (chiral column: CHIRALPAK AD-H; solvent: hexane:*i*-PrOH = 199:1; flow rate: 1.0 mL/min; detection: at 210 nm; rt): *t*_R_ = 10.1 min (major) and 11.8 min (minor).

***tert*-Butyl (2*R*,3*R*,*E*)-3-phenyl-2-(3-phenylpropyl)-5-(triethylsilyl)pent-4-enoate (3t):**

The title compound was obtained as a colorless oil (55.1 mg, 59%, dr >95:5, 99% ee) after purification by preparative TLC (hexane:EtOAc = 19:1). The enantioselectivity was determined by HPLC analysis after the reduction of **3t** with LiAlH_4_ in THF.

^1^H NMR (500 MHz, CDCl_3_) δ: 7.30–7.26 (m, 2H), 7.24–7.17 (m, 3H), 7.13 (d, *J* = 7.4 Hz, 3H), 7.05 (d, *J* = 7.4 Hz, 2H), 6.19 (dd, *J* = 18.9, 7.4 Hz, 1H), 5.61 (d, *J* = 18.3 Hz, 1H), 3.46 (dd, *J* = 10.3, 8.0 Hz, 1H), 2.67 (td, *J* = 10.5, 3.8 Hz, 1H), 2.56–2.50 (m, 1H), 2.43–2.37 (m, 1H), 1.62–1.38 (m, 12H), 1.24–1.14 (m, 1H), 0.88 (t, *J* = 8.0 Hz, 9H), 0.51 (q, *J* = 8.0 Hz, 6H); ^13^C NMR (125 MHz, CDCl_3_) δ: 174.2, 148.4, 142.2, 141.9, 128.5, 128.2, 128.2, 128.2, 126.9, 126.4, 125.6, 80.3, 55.5, 51.2, 35.4, 30.8, 28.9, 28.1, 7.3, 3.4; IR (neat): 3027, 2951, 2873, 1728, 1495, 1455, 1366, 1256, 1146, 1016, 700 cm^−1^; [α]^24^_D_ = −4.1 (*c* = 0.1, CHCl_3_, for 99% ee); HRMS (ESI-TOF): calcd for C_30_H_44_NaO_2_Si^+^: [M + Na]^+^ = 487.3003, found 487.2999; HPLC (chiral column: CHIRALPAK OD-H; solvent: hexane:*i*-PrOH = 199:1; flow rate: 1.0 mL/min; detection: at 210 nm; rt): *t*_R_ = 45.8 min (major) and 85.8 min (minor).

***tert*-Butyl (*R*)-6-phenoxy-2-[(*R*,*E*)-1-phenyl-3-(triethylsilyl)allyl]hexanoate (3u):**

The title compound was obtained as a colorless oil (59.6 mg, 60%, dr >95:5, 99% ee) after purification by preparative TLC (hexane:EtOAc = 19:1). The enantioselectivity was determined by HPLC analysis after the reduction of **3u** with LiAlH_4_ in THF.

^1^H NMR (500 MHz, CDCl_3_) δ: 7.31–7.22 (m, 4H), 7.21–7.17 (m, 1H), 7.15 (d, *J* = 8.0 Hz, 2H), 6.93–6.88 (m, 1H), 6.81 (dd, *J* = 8.6, 1.1 Hz, 2H), 6.20 (dd, *J* = 18.9, 7.4 Hz, 1H), 5.69–5.59 (m, 1H), 3.82 (t, *J* = 6.6 Hz, 2H), 3.48 (dd, *J* = 10.6, 7.7 Hz, 1H), 2.72–2.62 (m, 1H), 1.75–1.65 (m, 1H), 1.64–1.55 (m, 1H), 1.51–1.39 (m, 11H), 1.38–1.28 (m, 1H), 1.23–1.14 (m, 1H), 0.88 (t, *J* = 7.7 Hz, 9H), 0.51 (q, *J* = 8.0 Hz, 6H); ^13^C NMR (125 MHz, CDCl_3_) δ: 174.2, 159.0, 148.4, 141.9, 129.3, 128.5, 128.2, 126.8, 126.4, 120.4, 114.4, 80.4, 67.4, 55.4, 51.2, 30.8, 28.9, 28.1, 23.6, 7.3, 3.4; IR (neat): 3030, 2952, 2909, 2873, 1727, 1601, 1496, 1367, 1247, 1148, 757 cm^−1^; [α]^23^_D_ = −7.9 (*c* = 0.3, CHCl_3_, for 99% ee); HRMS (ESI-TOF): calcd for C_31_H_46_NaO_3_Si^+^: [M + Na]^+^ = 517.3108, found 517.3094; HPLC (chiral column: CHIRALPAK AD-H; solvent: hexane:*i*-PrOH = 199:1; flow rate: 1.0 mL/min; detection: at 210 nm; rt): *t*_R_ = 25.4 min (minor) and 29.3 min (major).

***tert*-Butyl (*R*)-8-chloro-2-[(*R*,*E*)-1-phenyl-3-(triethylsilyl)allyl]octanoate (3v):**

The title compound was obtained as a colorless oil (41.2 mg, 44%, dr >95:5, 97% ee) after purification by preparative TLC (hexane:EtOAc = 19:1). The enantioselectivity was determined by HPLC analysis after the reduction of **3v** with LiAlH_4_ in THF.

^1^H NMR (500 MHz, CDCl_3_) δ: 7.31–7.27 (m, 2H), 7.21–7.17 (m, 1H), 7.14 (d, *J* = 6.9 Hz, 2H), 6.19 (dd, *J* = 18.9, 7.4 Hz, 1H), 5.68–5.58 (m, 1H), 3.49–3.44 (m, 3H), 2.62 (td, *J* = 10.7, 3.6 Hz, 1H), 1.72–1.64 (m, 2H), 1.44 (s, 9H), 1.34–1.06 (m, 8H), 0.88 (t, *J* = 7.7 Hz, 9H), 0.51 (q, *J* = 8.0 Hz, 6H); ^13^C NMR (125 MHz, CDCl_3_) δ: 174.3, 148.5, 142.0, 128.4, 128.2, 126.8, 126.4, 80.3, 55.5, 51.2, 45.0, 32.4, 30.9, 28.4, 28.2, 26.9, 26.6, 7.3, 3.4; IR (neat): 3027, 2952, 2935, 2873, 1727, 1455, 1367, 1148, 1016, 759, 724 cm^−1^; [α]^24^_D_ = −9.8 (*c* = 0.2, CHCl_3_, for 97% ee); HRMS (ESI-TOF): calcd for C_27_H_45_ClNaO_2_Si^+^: [M + Na]^+^ = 487.2770, found 487.2790; HPLC (chiral column: CHIRALPAK IB; solvent: hexane:*i*-PrOH = 199:1; flow rate: 1.0 mL/min; detection: at 210 nm; rt): *t*_R_ = 10.5 min (major) and 11.5 min (minor).

1. **Derivatization of 4-Pentenoate 3**
   1. **Desilylation of 4-Pentenoate 3a**
      1. **Experimental Procedure**

To an oven-dried test tube containing the product oil **3a** (48.3 mg, 0.152 mmol) in CH_3_CN (3.0 mL), *p*-toluenesulfonic acid monohydrate (28.8 mg, 0.152 mmol) was added at room temperature, and the mixture was stirred at 90 °C. After 3 h, the solution was concentrated in vacuo. The residue was transferred to a 10 mL flask containing K_2_CO_3_ (62.9 mg, 0.455 mmol), MeI (64.6 mg, 0.455 mmol), and DMF (2.0 mL). The mixture was stirred at room temperature for 2 h. After this time, the reaction was quenched with H₂O (5 mL), extracted with EtOAc (3 × 10 mL), concentrated, and purified by preparative thin-layer chromatography (hexane:EtOAc = 30:1) to afford the desilylated products **4** (26.9 mg, 0.132 mmol, 83%).

- - 1. **Characterization of Desilylated Product 4**

**Methyl-2-methyl-3-phenylpent-4-enoate (4):**

The title compound was obtained as a colorless oil in a mixture of diastereomers (26.9 mg, 83%, dr 90:10, 99% ee) after purification by preparative TLC (hexane:EtOAc = 30:1). The enantioselectivity was determined by HPLC analysis after the reduction of **4** with LiAlH_4_ in THF.

*syn*-**4** diastereomer (minor): ^1^H NMR (500 MHz, CDCl_3_) δ: 7.33–7.17 (m, 5H), 6.05–5.85 (m, 1H), 5.17–4.96 (m, 2H), 3.68 (s, 3H), 3.54–3.38 (m, 1H), 2.88–2.78 (m, 1H), 1.22 (d, *J* = 6.9 Hz, 3H); (2*R,*3*S*)-*anti*-**4** diastereomer (major): ^1^H NMR (500 MHz, CDCl_3_) δ: 7.33–7.17 (m, 5H), 6.05–5.85 (m, 1H), 5.17–4.96 (m, 2H), 3.68 (s, 3H), 3.54–3.38 (m, 1H), 2.88–2.78 (m, 1H), 0.97 (d, *J* = 6.9 Hz, 3H); ^13^C NMR (125 MHz, CDCl_3_) δ: 176.1, 141.2, 139.7, 128.6, 128.0, 126.7, 115.4, 53.7, 51.5, 45.2, 15.8; IR (neat): 3083, 3062, 3029, 2980, 2950, 2937, 1738, 1456, 1435, 1255, 1194, 1168, 766, 704 cm^−1^; HRMS (ESI-TOF): calcd for C_13_H_16_NaO_2_^+^: [M + Na]^+^ = 227.1043, found 227.1049; HPLC (chiral column: CHIRALPAK AD-H; solvent: hexane:*i*-PrOH = 199:1; flow rate: 0.7 mL/min; detection: at 210 nm; rt): *t*_R_ = 51.3 min (minor) and 61.6 min (major).

- 1. **Acetylation of 4-Pentenoate 3a**
     1. **Experimental Procedure**

To an oven-dried test tube containing the product oil **3a** (54.2 mg, 0.170 mmol) in 1,4-dioxane (0.4 mL), [RhCl(CO)_2_]_2_ (3.3 mg, 0.008 mmol) and acetic anhydride (52.2 mg, 0.510 mmol) were added at room temperature, and the mixture was stirred at 90 °C. After 48 h, the solution was filtered through a plug of Florisil^®^, washed with EtOAc, and the filtrate was concentrated in vacuo. The residue was purified by preparative thin-layer chromatography (toluene:EtOAc = 9:1) to afford the desired acetylated product **5** (29.6 mg, 0.103 mmol, 60%).

- - 1. **Characterization of Acetylated Product 5**

***tert*-Butyl (2*S*,3*S*,*E*)-2-methyl-6-oxo-3-phenylhept-4-enoate (5):**

The title compound was obtained as a colorless oil in a mixture of diastereomers (29.6 mg, 60%, dr 86:14, 98% ee) after purification by preparative TLC (toluene:EtOAc = 9:1). The enantioselectivity was determined by HPLC analysis after the reduction of **5** with LiAlH_4_ in THF.

^1^H NMR (500 MHz, CDCl_3_) δ: 7.28–7.24 (m, 2H), 7.21–7.16 (m, 1H), 7.11 (d, *J* = 7.4 Hz, 2H), 6.85 (dd, *J* = 16.0, 8.6 Hz, 1H), 6.00 (d, *J* = 16.0 Hz, 1H), 3.47 (dd, *J* = 10.3, 8.6 Hz, 1H), 2.70 (dt, *J* = 17.8, 6.9 Hz, 1H), 2.14 (s, 3H), 1.36 (s, 9H), 0.90 (d, *J* = 6.9 Hz, 3H); ^13^C NMR (125 MHz, CDCl_3_) δ: 198.3, 174.3, 148.0, 139.4, 131.0, 128.9, 128.2, 127.3, 80.9, 52.2, 45.4, 28.0, 27.0, 16.0; IR (neat): 3029, 2978, 2934, 1726, 1677, 1626, 1455, 1368, 1254, 1153, 983, 703 cm^−1^; [α]^24^_D_ = −33.4 (*c* = 0.2, CHCl_3_, for 98% ee); HRMS (ESI-TOF): calcd for C_18_H_24_NaO_3_^+^: [M + Na]^+^ = 311.1618, found 311.1625; HPLC (chiral column: CHIRALPAK AD-H; solvent: hexane:*i*-PrOH = 199:1; flow rate: 0.7 mL/min; detection: at 210 nm; rt): *t*_R_ = 30.0 min (minor) and 35.4 min (major).

- 1. **Arylation of 4-Pentenoate 3p**
     1. **Experimental Procedure**

To an oven-dried test tube containing 4-pentenoate **3p** (61.6 mg, 0.156 mmol) and 4-iodoanisole (54.7 mg, 0.234 mmol) in dry THF (0.16 mL), TBAF (1 M solution in THF, 0.32 mL, 0.32 mmol) was added, and the mixture was stirred at room temperature. After 30 minutes, Pd_2_(dba)_3_ (3.5 mg, 0.004 mmol) was added. The reaction mixture was then stirred for 1 hour at room temperature and filtered through a plug of Florisil^®^. The filtrate was concentrated under reduced pressure, and the residue was purified by preparative thin-layer chromatography (hexane:EtOAc = 10:1), yielding the arylated product **6** (40.2 mg, 0.114 mmol, 73%).

- - 1. **Characterization of Arylated Product 6**

***tert*-Butyl (2*R*,3*S*,*E*)-5-(4-methoxyphenyl)-2-methyl-3-phenylpent-4-enoate (6):**

The title compound was obtained as a colorless oil in a mixture of diastereomers (40.2 mg, 73%, dr 92:8, 99% ee) after purification by preparative TLC (toluene:EtOAc = 10:1).

*anti*-**6** diastereomer (minor): ^1^H NMR (500 MHz, CDCl_3_) δ: 7.33–7.20 (m, 7H), 6.82–6.79 (m, 2H), 6.40 (d, *J* = 15.5 Hz, 1H), 6.09 (dd, *J* = 15.7, 9.5 Hz, 1H), 3.78 (s, 3H), 3.50 (dd, *J* = 9.5, 9.5 Hz, 1H), 2.81–2.74 (m, 1H), 1.23 (d, *J* = 6.9 Hz, 3H), 1.16 (s, 9H); ^13^C NMR (125 MHz, CDCl_3_) δ: 174.6, 158.9, 142.7, 130.8, 129.9, 128.7, 128.3, 128.0, 127.3, 126.6, 113.8, 79.9, 55.2, 53.5, 45.9, 27.6, 16.1; (2*R,*3*S*)-*syn*-**6** diastereomer (major): ^1^H NMR (500 MHz, CDCl_3_) δ: 7.33–7.20 (m, 7H), 6.82–6.79 (m, 2H), 6.36 (d, *J* = 16.0 Hz, 1H), 6.23 (q, *J* = 8.0 Hz, 1H), 3.77 (s, 3H), 3.50 (dd, *J* = 9.5, 9.5 Hz, 1H), 2.81–2.74 (m, 1H), 1.38 (s, 9H), 0.96 (d, *J* = 6.9 Hz, 3H); ^13^C NMR (125 MHz, CDCl_3_) δ: 75.0, 158.8, 142.0, 130.1, 129.9, 129.4, 128.6, 128.0, 127.3, 126.6, 113.8, 80.3, 55.2, 53.3, 46.4, 28.0, 16.1; IR (neat): 3029, 2976, 2933, 2836, 1725, 1607, 1511, 1454, 1367, 1250, 1149, 1035 cm^−1^; HRMS (Dart): calcd for C_23_H_29_O_3_^+^: [M + H]^+^ = 353.2111, found 353.2112; HPLC (chiral column: CHIRALPAK OD-H; solvent: hexane:*i*-PrOH = 199:1; flow rate: 0.8 mL/min; detection: at 254 nm; 60 °C): *t*_R_ = 8.8 min (minor) and 10.9 min (major).

1. **Mechanistic Investigations**
   1. **Hydroallylation with α-Silyl Allyl Acetate 7**
      1. **Experimental Procedure**

To an oven-dried test tube containing Cu(OAc)_2_ (1.8 mg, 0.010 mmol) and (*R*)-tol-BINAP (7.5 mg, 0.011 mmol) in dry DME (0.6 mL) was added HBpin (38.4 mg, 0.300 mmol) at room temperature, and the mixture was stirred for 15 min at the same temperature (Solution A). In a separate oven-dried test tube, Pd(dba)_2_ (6.9 mg, 0.012 mmol), RuPhos (6.2 mg, 0.013 mmol), and dry DME (0.4 mL) were combined and stirred at 50 °C for 15 min (Solution B). Solution B (0.2 mL), acrylate **1a** (25.5 mg, 0.199 mmol), and allyl acetate **7** (59.7 mg, 0.240 mmol) were then added to Solution A via syringe at 40 °C. After 18 h, the solution was filtered through a plug of Florisil^®^, washed with EtOAc, and the filtrate was concentrated under reduced pressure. The residue was purified by preparative thin-layer chromatography (hexane:EtOAc = 19:1), yielding the desired hydroallylation product **3w** (49.8 mg, 0.156 mmol, 78%).

- - 1. **Characterization of 4-Pentenoate 3w**

***tert*-Butyl (*E*)-5-[dimethyl(phenyl)silyl]-2,3-dimethylpent-4-enoate (3w):**

The title compound was obtained as a colorless oil in a mixture of diastereomers (49.8 mg, 78%, dr 90:10, 92% ee) after purification by preparative TLC (toluene:EtOAc = 19:1). The enantioselectivity was determined by HPLC analysis after the reduction of **3w** with LiAlH_4_ in THF.

*anti*-**3w** diastereomer (minor): ^1^H NMR (500 MHz, CDCl_3_) δ: 7.53–7.48 (m, 2H), 7.37–7.31 (m, 3H), 5.93 (dd, *J* = 18.93, 7.4 Hz, 1H), 5.81–5.74 (m, 1H), 2.56–2.42 (m, 1H), 2.24–2.19 (m, 1H), 1.43 (s, 9H), 1.04 (d, *J* = 6.9 Hz, 3H), 1.00 (d, *J* = 6.9 Hz, 3H), 0.31 (s, 6H). (2*R,*3*R*)-*syn*-**3w** diastereomer (major): ^1^H NMR (500 MHz, CDCl_3_) δ: 7.53–7.48 (m, 2H), 7.37–7.31 (m, 3H), 6.06 (dd, *J* = 18.9, 6.9 Hz, 1H), 5.81–5.74 (m, 1H), 2.56–2.42 (m, 1H), 2.36–2.27 (m, 1H), 1.40 (s, 9H), 1.04 (d, *J* = 6.9 Hz, 3H), 1.00 (d, *J* = 6.9 Hz, 3H), 0.31 (s, 6H); ^13^C NMR (125 MHz, CDCl_3_) δ: 174.9, 151.7, 139.0, 133.8, 128.8, 127.7, 126.7, 79.8, 45.2, 42.5, 28.1, 15.8, 13.5, −2.4, −2.5; IR (neat): 3070, 2976, 2933, 1730, 1367, 1250, 1151, 1114, 993, 848, 732, 700 cm^−1^; HRMS (ESI-TOF): calcd for C_19_H_30_NaO_2_Si^+^: [M + Na]^+^ = 341.1907, found 341.1909; HPLC (chiral column: CHIRALPAK AD-H; solvent: hexane:*i*-PrOH = 199:1; flow rate: 1.0 mL/min; detection: at 210 nm; rt): *t*_R_ = 16.3 min (minor) and 18.0 min (major).

- 1. **Hydroallylation with *E*/*Z* Mixture of Allyl Acetate 2a′**
     1. **Experimental Procedure**

To an oven-dried test tube containing Cu(OAc)_2_ (1.8 mg, 0.010 mmol) and (*R*)-tol-BINAP (7.5 mg, 0.011 mmol) in dry DME (0.6 mL) was added HBpin (38.4 mg, 0.300 mmol) at room temperature, and the mixture was stirred for 15 min at the same temperature (Solution A). In a separate oven-dried test tube, Pd(dba)_2_ (6.9 mg, 0.012 mmol), RuPhos (6.2 mg, 0.013 mmol), and dry DME (0.4 mL) were combined and stirred at 50 °C for 15 min (Solution B). Solution B (0.2 mL), acrylate **1a** (25.4 mg, 0.198 mmol), and allyl acetate **2a′** (59.6 mg, 0.240 mmol) were then added to Solution A via syringe at 40 °C. After 18 h, the solution was filtered through a plug of Florisil^®^, washed with EtOAc, and the filtrate was concentrated under reduced pressure. The residue was purified by preparative thin-layer chromatography (hexane:EtOAc = 19:1), yielding the desired hydroallylation product **3a** (54.0 mg, 0.170 mmol, 86%, dr 90:10, 98% ee).

- 1. **Hydroallylation with γ-*tert*-Butyl Allyl Acatate 8**
     1. **Experimental Procedure**

To an oven-dried test tube containing Cu(OAc)_2_ (1.8 mg, 0.010 mmol) and (*R*)-tol-BINAP (7.5 mg, 0.011 mmol) in dry DME (0.6 mL) was added HBpin (38.4 mg, 0.300 mmol) at room temperature, and the mixture was stirred for 15 min at the same temperature (Solution A). In a separate oven-dried test tube, Pd(dba)_2_ (6.9 mg, 0.012 mmol), RuPhos (6.2 mg, 0.013 mmol), and dry DME (0.4 mL) were combined and stirred at 50 °C for 15 min (Solution B). Solution B (0.2 mL), acrylate **1a** (26.0 mg, 0.203 mmol), and allyl acetate **8** (55.8 mg, 0.240 mmol) were then added to Solution A via syringe at 40 °C. After 18 h, the solution was filtered through a plug of Florisil^®^, washed with EtOAc, and the filtrate was concentrated under reduced pressure. The residue was purified by preparative thin-layer chromatography (hexane:EtOAc = 19:1), yielding the desired hydroallylation product **9** (39.7 mg, 0.131 mmol, 64%).

- - 1. **Characterization of 4-Heptanoate 9**

***tert*-Butyl (2*R*,3*S*,*E*)-2,6,6-trimethyl-3-phenylhept-4-enoate (9):**

The title compound was obtained as a colorless oil in a mixture of diastereomers (39.7 mg, 64%, dr 92:8, 98% ee) after purification by preparative TLC (toluene:EtOAc = 19:1). The enantioselectivity was determined by HPLC analysis after the reduction of **9** with LiAlH_4_ in THF.

^1^H NMR (500 MHz, CDCl_3_) δ: 7.31–7.27 (m, 2H), 7.21–7.17 (m, 1H), 7.16 (d, *J* = 6.9 Hz, 2H), 5.52 (d, *J* = 3.4 Hz, 2H), 3.39–3.28 (m, 1H), 2.70–2.57 (m, 1H), 1.44 (s, 9H), 0.96 (s, 9H), 0.89 (d, *J* = 6.9 Hz, 3H); ^13^C NMR (125 MHz, CDCl_3_) δ: 175.1, 142.2, 128.4, 128.1, 128.1, 126.2, 126.2, 80.0, 52.2, 46.4, 32.9, 29.6, 28.2, 16.4; IR (neat): 3029, 2963, 2903, 2869, 1729, 1456, 1367, 1151, 969, 701 cm^−1^; HRMS (ESI-TOF): calcd for C_20_H_30_NaO_2_^+^: [M + Na]^+^ = 325.2138, found 325.2145; HPLC (chiral column: CHIRALPAK OJ-H; solvent: hexane:*i*-PrOH = 199:1; flow rate: 1.0 mL/min; detection: at 210 nm; rt): *t*_R_ = 8.3 min (minor) and 10.1 min (major).

- 1. **^31^P NMR Analysis of Mixture of Copper and Palladium Catalysts**
     1. **Experimental Procedure for Mixture of Cu/Ruphos**

To an oven-dried test tube containing Cu(OAc)_2_ (1.8 mg, 0.010 mmol) and RuPhos (4.7 mg, 0.010 mmol) in THF-*d*_8_ (0.6 mL) was added HBpin (9.5 mg, 0.074 mmol) at room temperature. The mixture was stirred for 15 min at the same temperature. The solution was transferred to a dried NMR tube and submitted to NMR analysis.

- - 1. **Experimental Procedure for Mixture of Pd/(*R*)-tol-BINAP**

In an oven-dried test tube, Pd(dba)_2_ (5.7 mg, 0.010 mmol), (*R*)-tol-BINAP (6.8 mg, 0.010 mmol), and THF-*d*_8_ (0.6 mL) were stirred at 50 °C for 15 min. The solution was transferred to a dried NMR tube and submitted to NMR analysis.

- - 1. **Experimental Procedure for Mixture of Cu/(*R*)-tol-BINAP**

To an oven-dried test tube containing Cu(OAc)_2_ (1.8 mg, 0.010 mmol) and (*R*)-tol-BINAP (6.8 mg, 0.010 mmol) in THF-*d*_8_ (0.6 mL) was added HBpin (10.3 mg, 0.080 mmol) at room temperature. The mixture was stirred for 15 min at the same temperature. The solution was transferred to a dried NMR tube and submitted to NMR analysis.

- - 1. **Experimental Procedure for Mixture of Pd/Ruphos**

In a separate oven-dried test tube, Pd(dba)_2_ (5.8 mg, 0.010 mmol), RuPhos (4.7 mg, 0.010 mmol), and THF-*d*_8_ (0.6 mL) were stirred at 50 °C for 15 min. The solution was transferred to a dried NMR tube and submitted to NMR analysis.

- - 1. **Experimental Procedure for Mixture of Cu/(*R*)-tol-BINAP and Pd/Ruphos**

To an oven-dried test tube containing Cu(OAc)_2_ (1.8 mg, 0.010 mmol) and (*R*)-tol-BINAP (6.8 mg, 0.010 mmol) in THF-*d*_8_ (0.6 mL) was added HBpin (9.1 mg, 0.071 mmol) at room temperature. The mixture was stirred for 15 min at the same temperature (Solution A). In a separate oven-dried test tube, Pd(dba)_2_ (5.8 mg, 0.010 mmol), RuPhos (4.7 mg, 0.010 mmol), and THF-*d*_8_ (0.6 mL) were stirred at 50 °C for 15 min (Solution B). Solution B (0.6 mL) were then added to Solution A via syringe at 40 °C. After 18 h, the solution was transferred to a dried NMR tube and submitted to NMR analysis.

- - 1. **^31^P NMR Spectra**

Cu/(*R*)-tol-BINAP + Pd/RuPhos

Cu/(*R*)-tol-BINAP

Pd/RuPhos

Pd/(*R*)-tol-BINAP

Cu/RuPhos

RuPhos

(*R*)-tol-BINAP

1. **References**

[86] N. Homura, K. Inoue, S. Yoshikawa, Y. Horino, *Adv. Synth. Catal.* **2012**, *354*, 828–834.

[87] M. A. Kacprzynski, T. L. May, S. A. Kazane, A. H. Hoveyda, *Angew. Chem. Int. Ed.* **2007**, *46*, 4554–4558.

[88] J. I. Bowen, L. Wang, M. P. Crump, C. L. Willis, *Org. Biomol. Chem.* **2021**, *19*, 6210–6215.

[89] T. Muraoka, I. Matsuda, K. Ueno, *Organometallics* **2007**, *26*, 387–396.

[90] A. Ohzono, K. Shirota, R. Shintani, *Chem. Commun.* **2020**, *56*, 11851–11854.

[52] N. Kranidiotis-Hisatomi, H. Yi, M. Oestreich, *Angew. Chem. Int. Ed.* **2021**, *60*, 13652–13655.

[91] D. H. Lee, E. J. Corey, *J. Am. Chem. Soc.* **1991**, *113*, 4026–4028.

1. **Copies of ^1^H, ^13^C and ^19^F NMR Spectra for the Products**


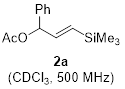
**
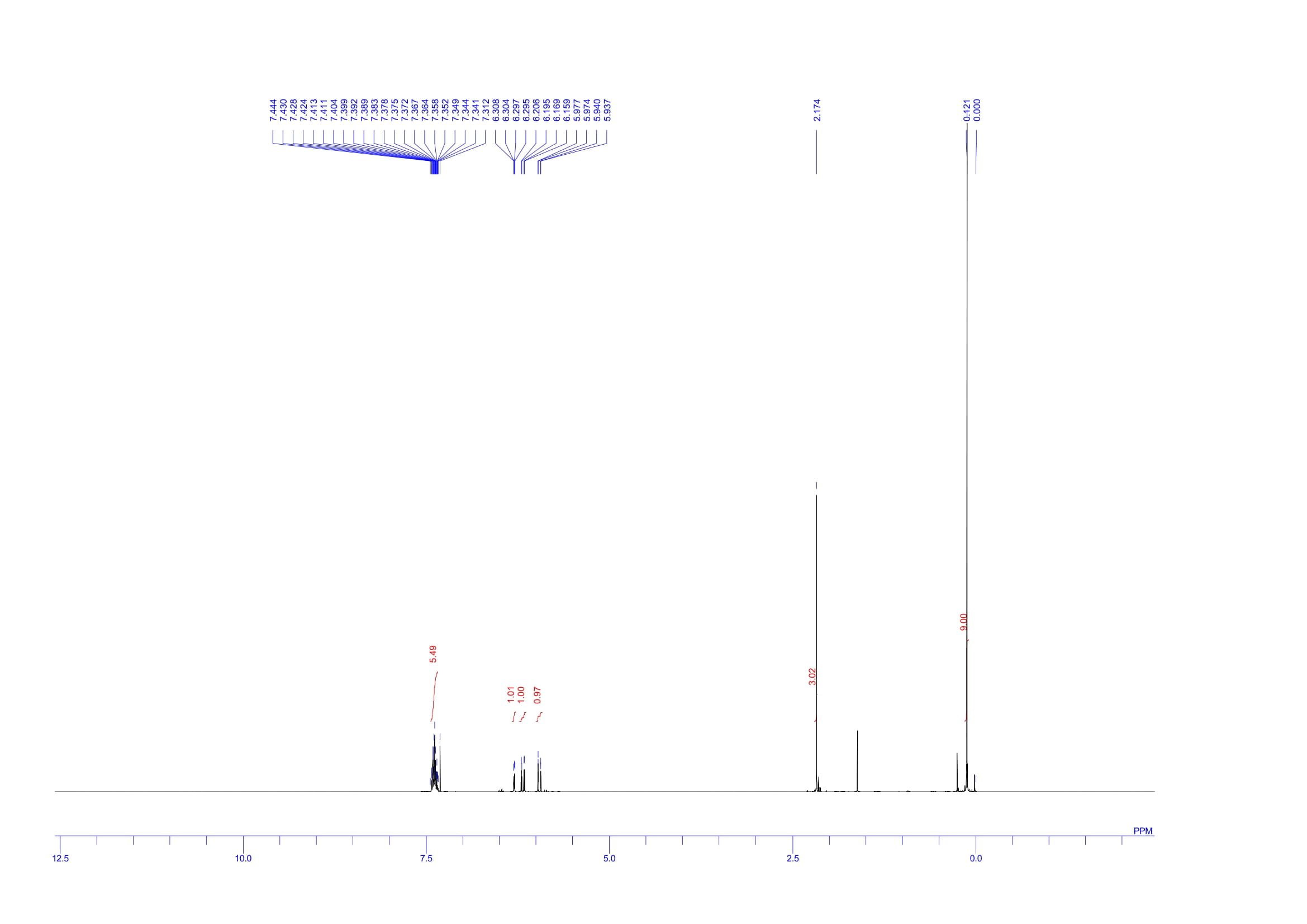
**


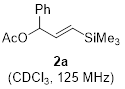
**
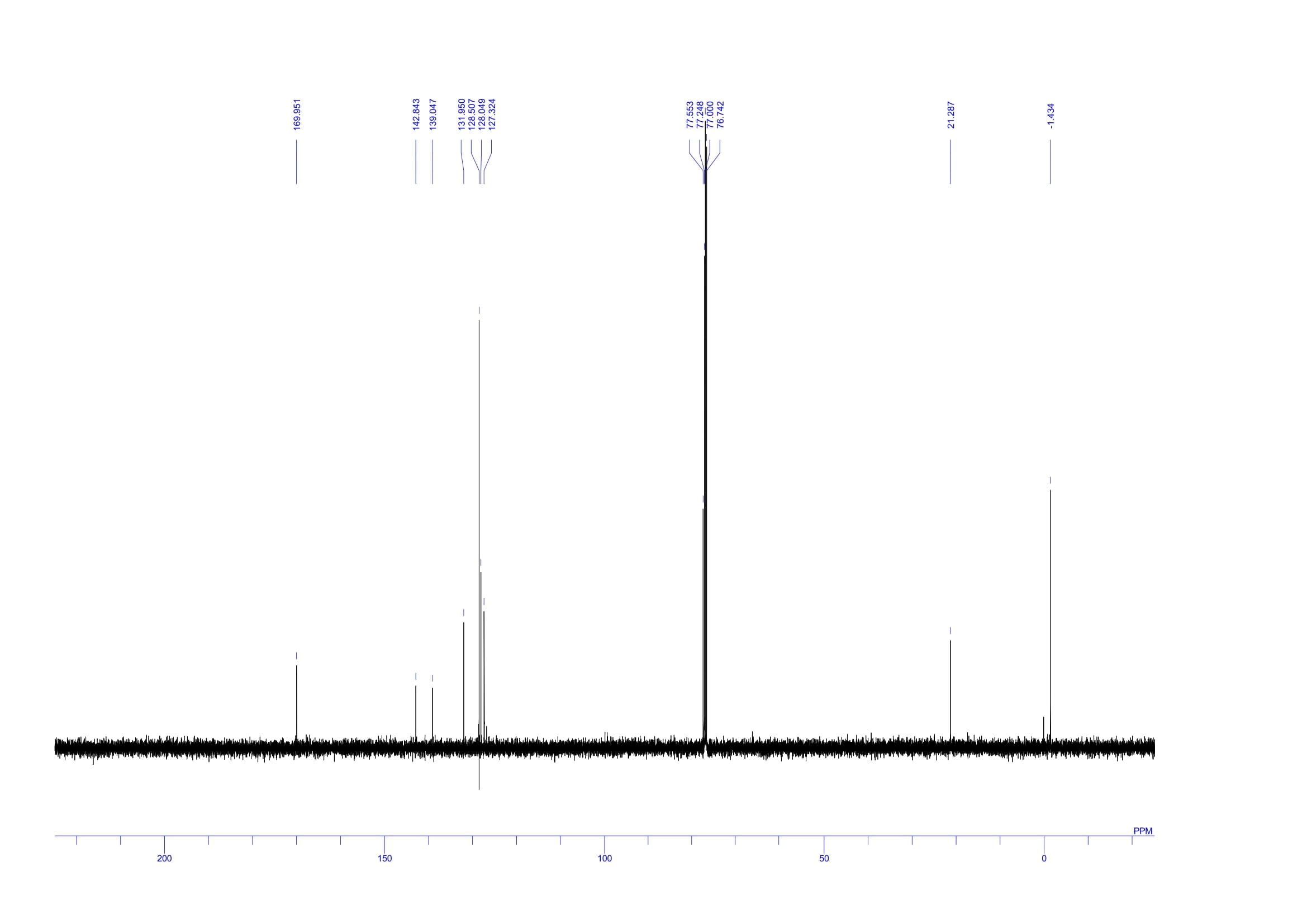
**


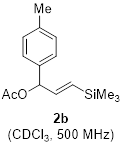
**
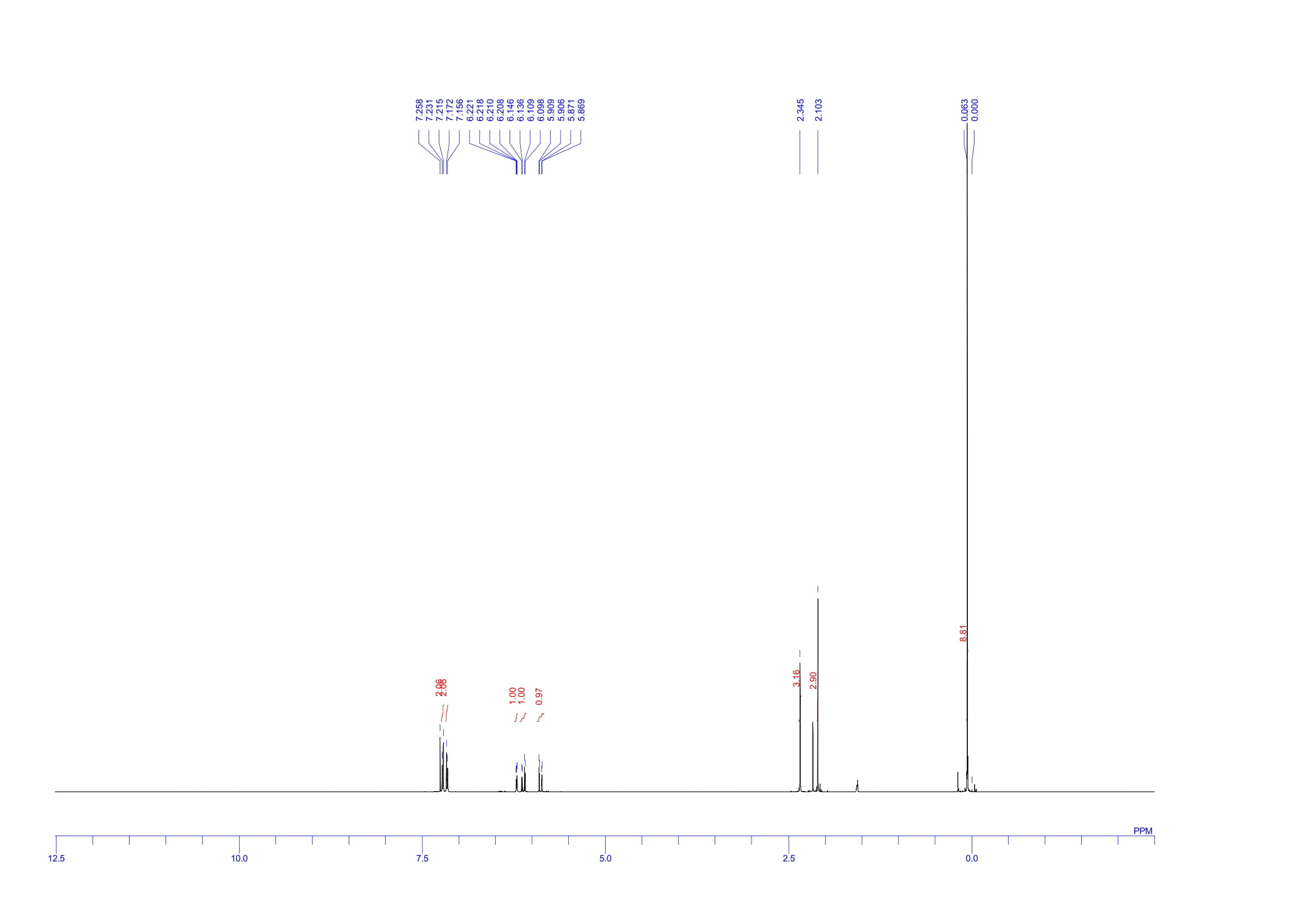
**


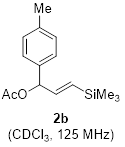
**
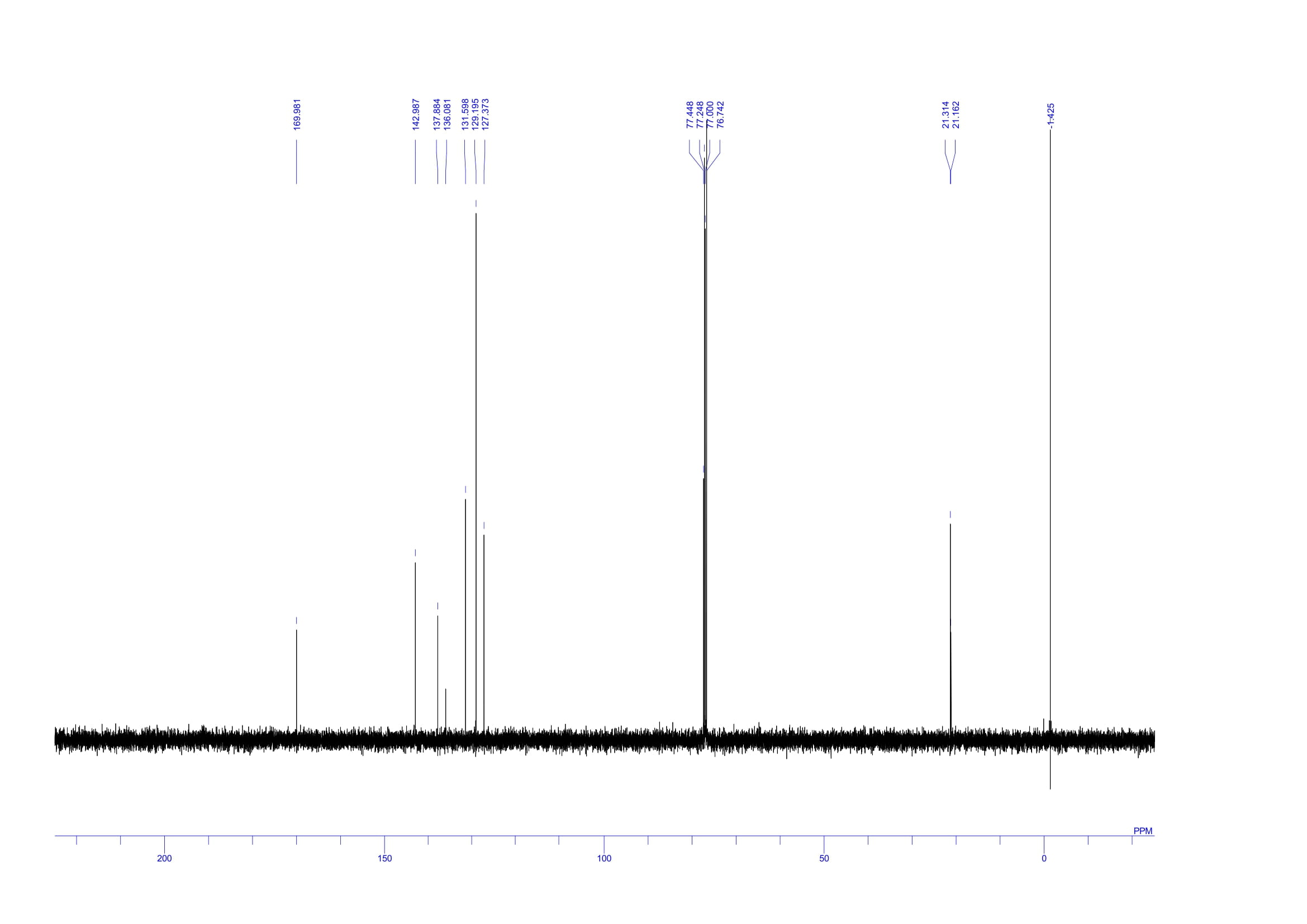
**


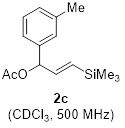
**
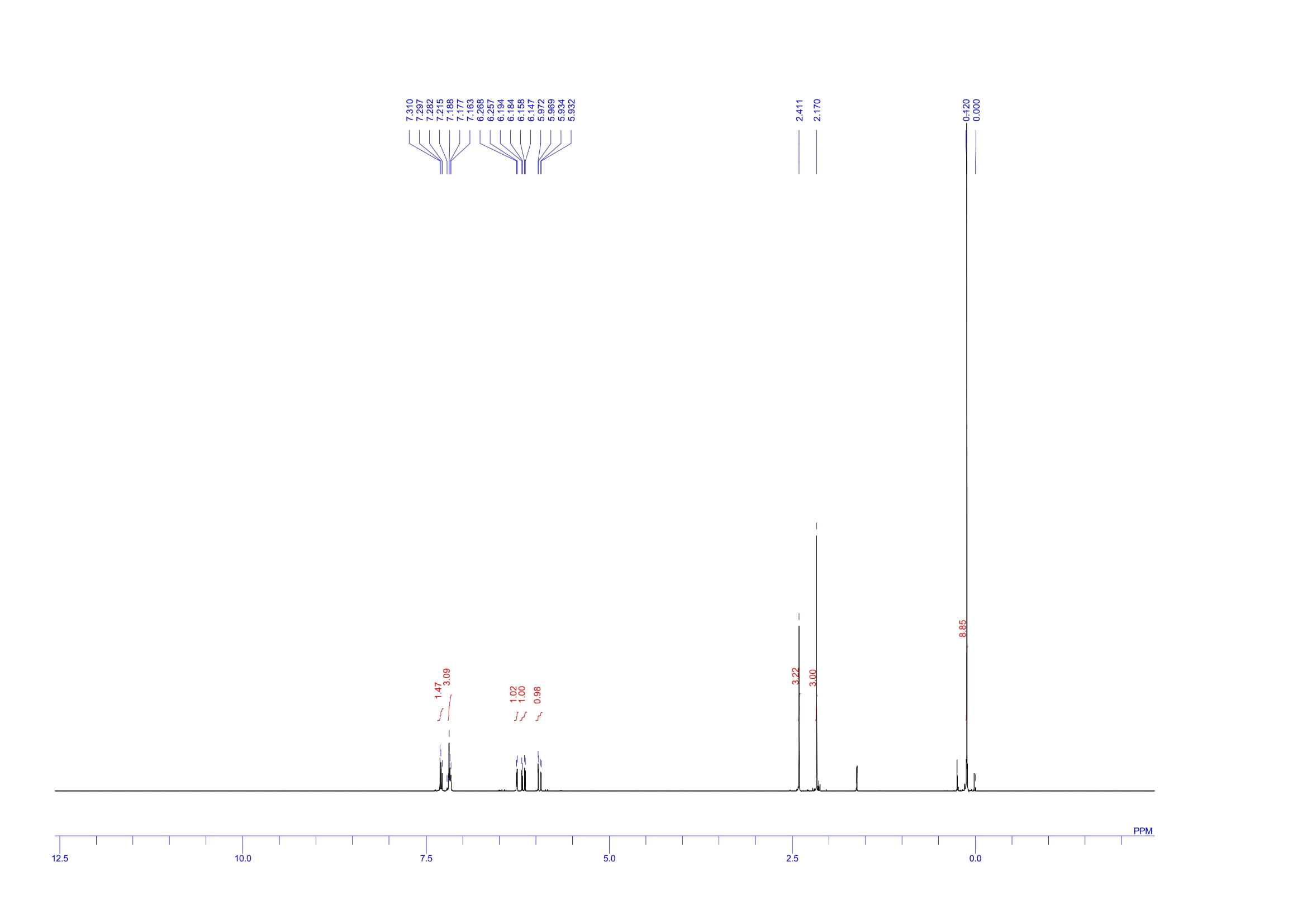
**


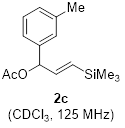
**
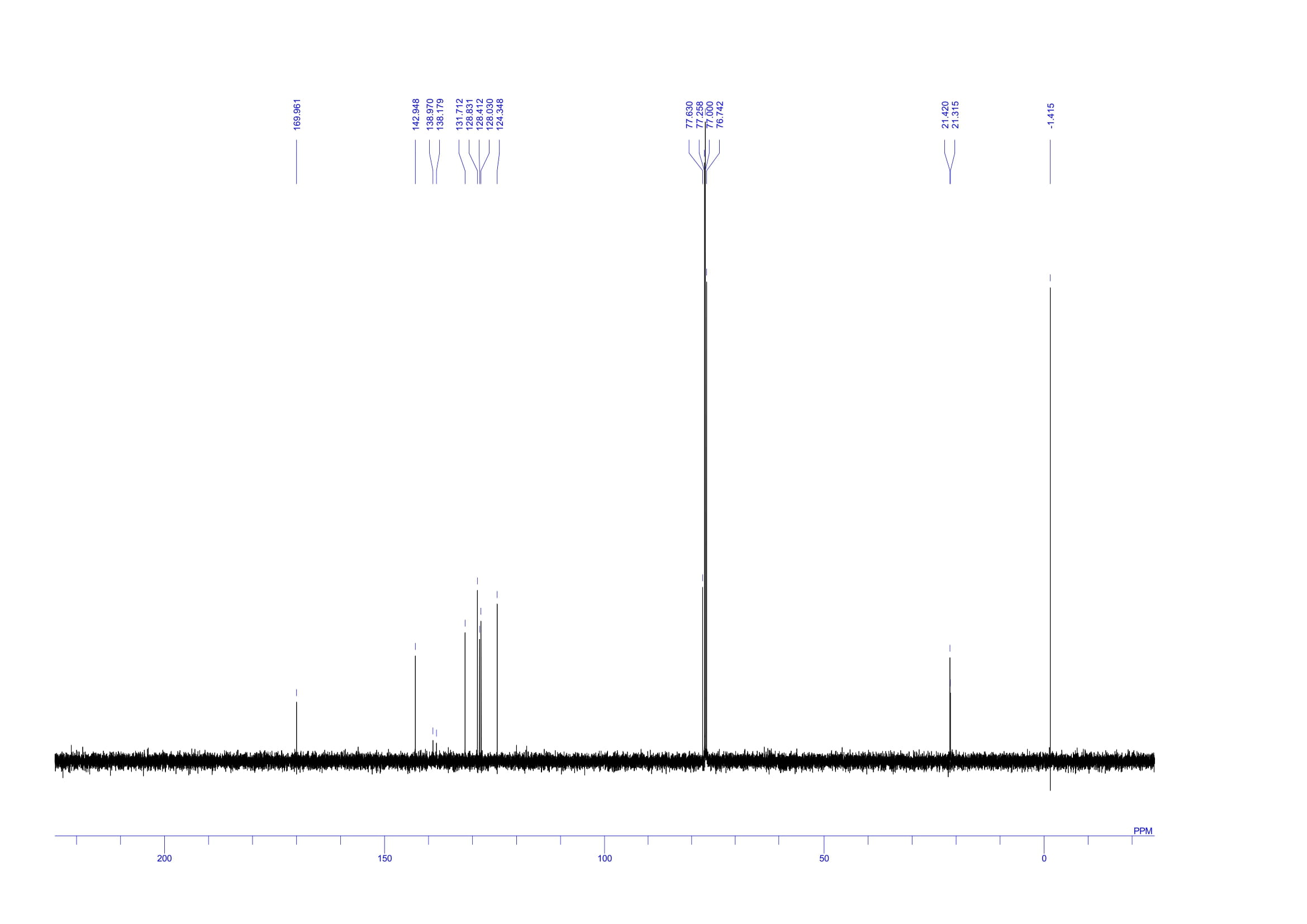
**


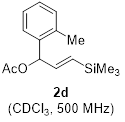
**
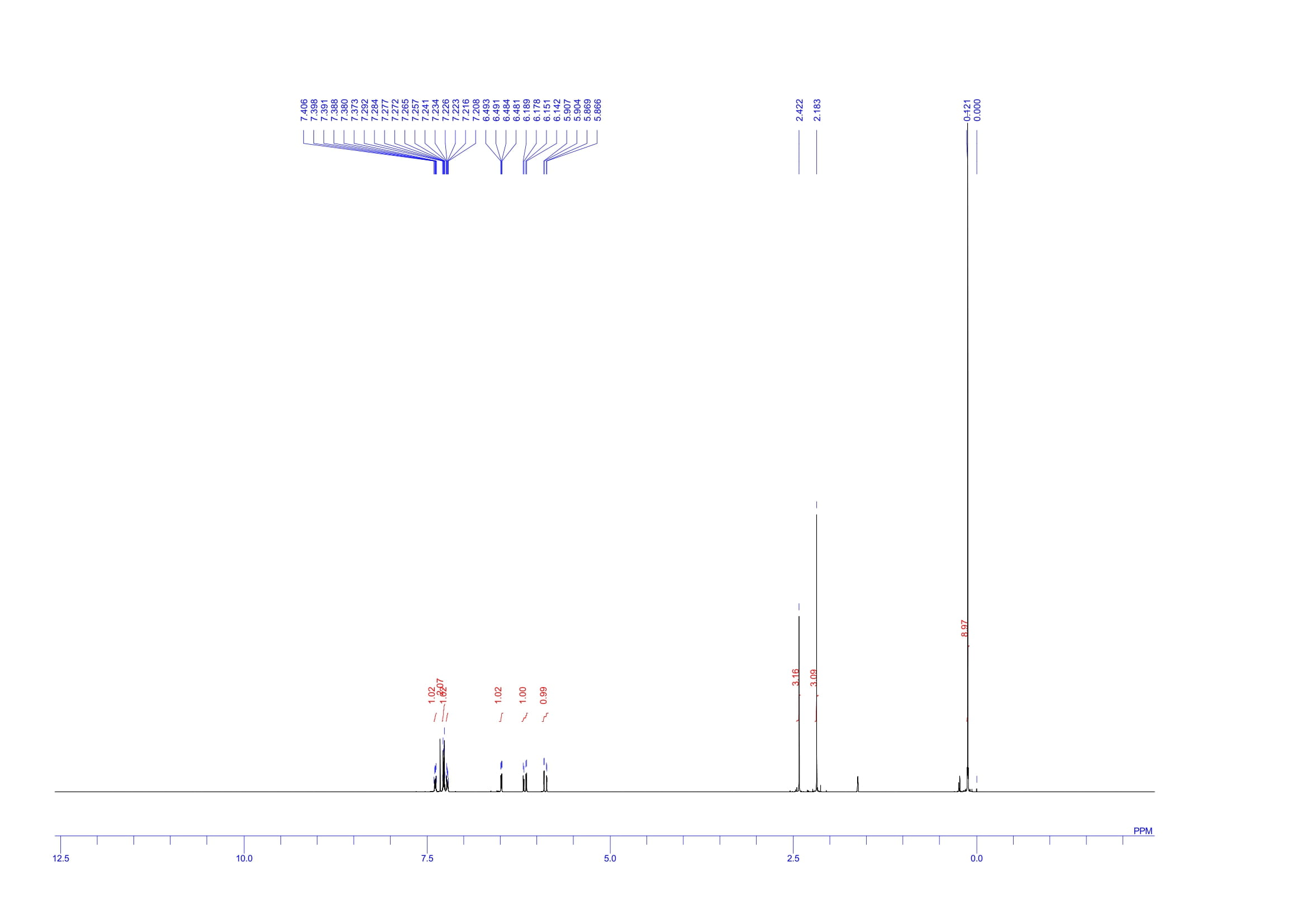
**


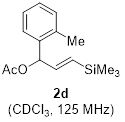
**
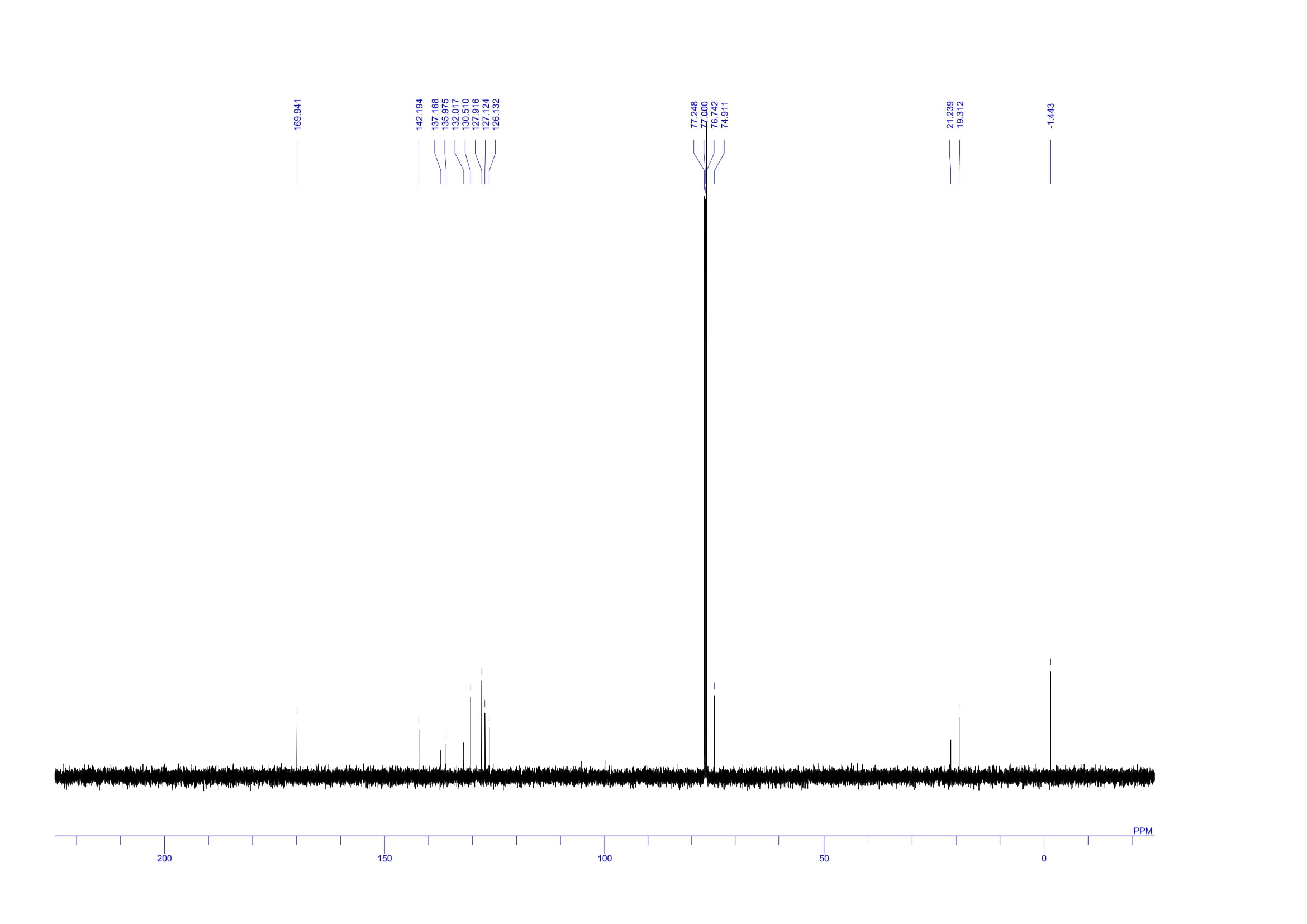
**


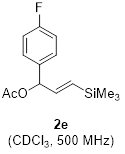
**
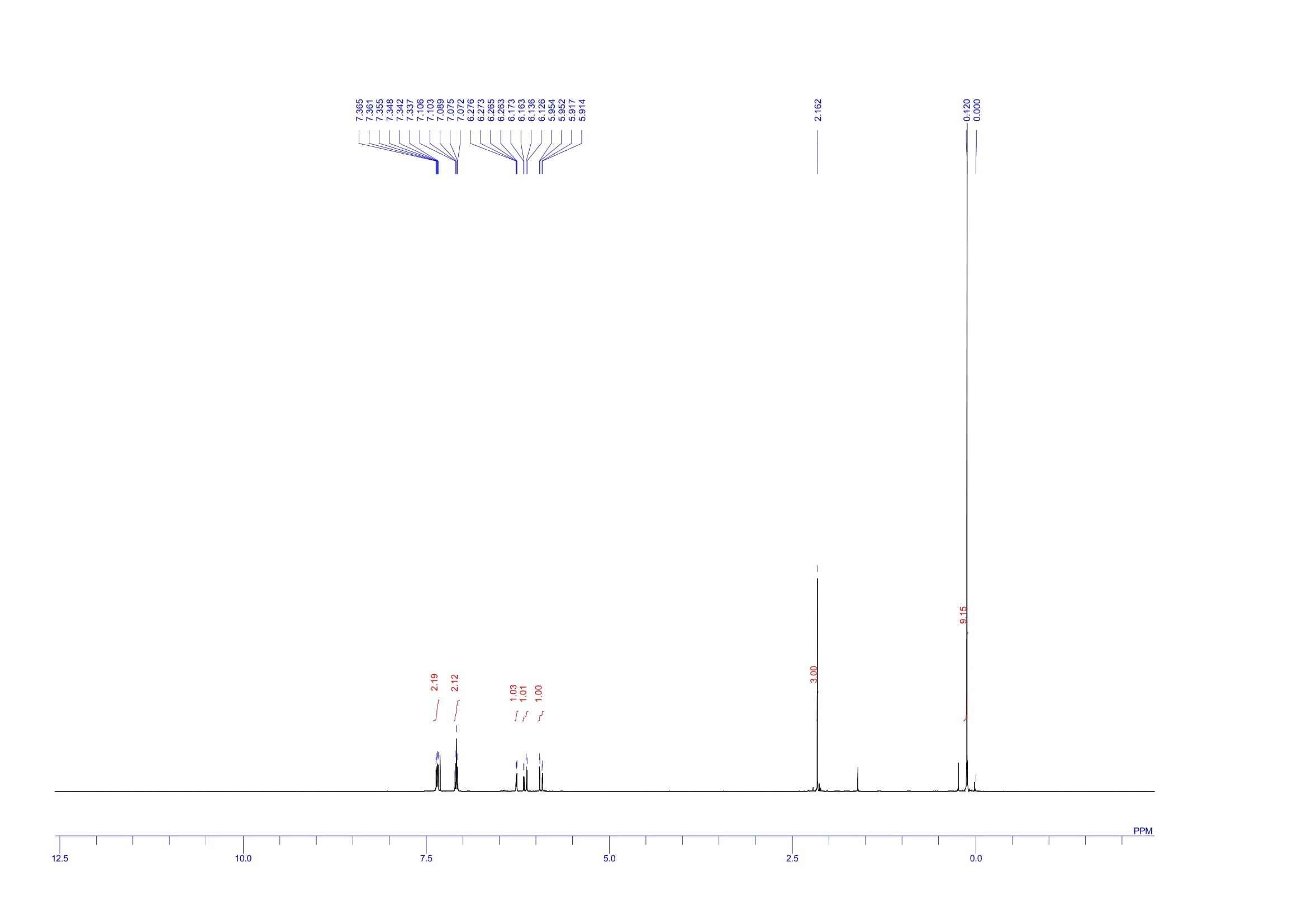
**


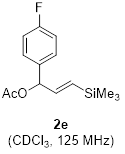
**
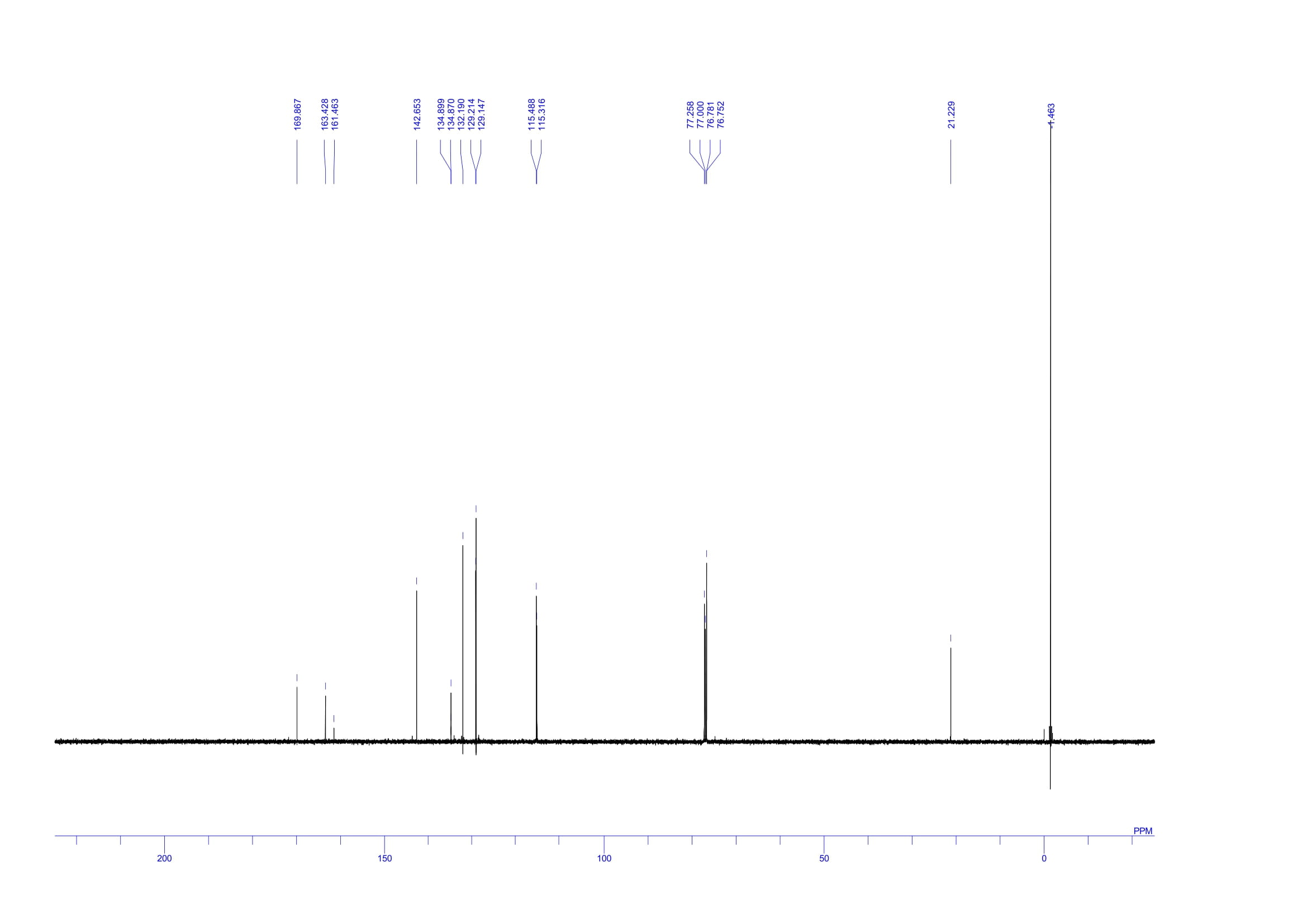
**


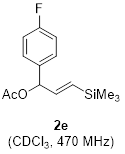
**
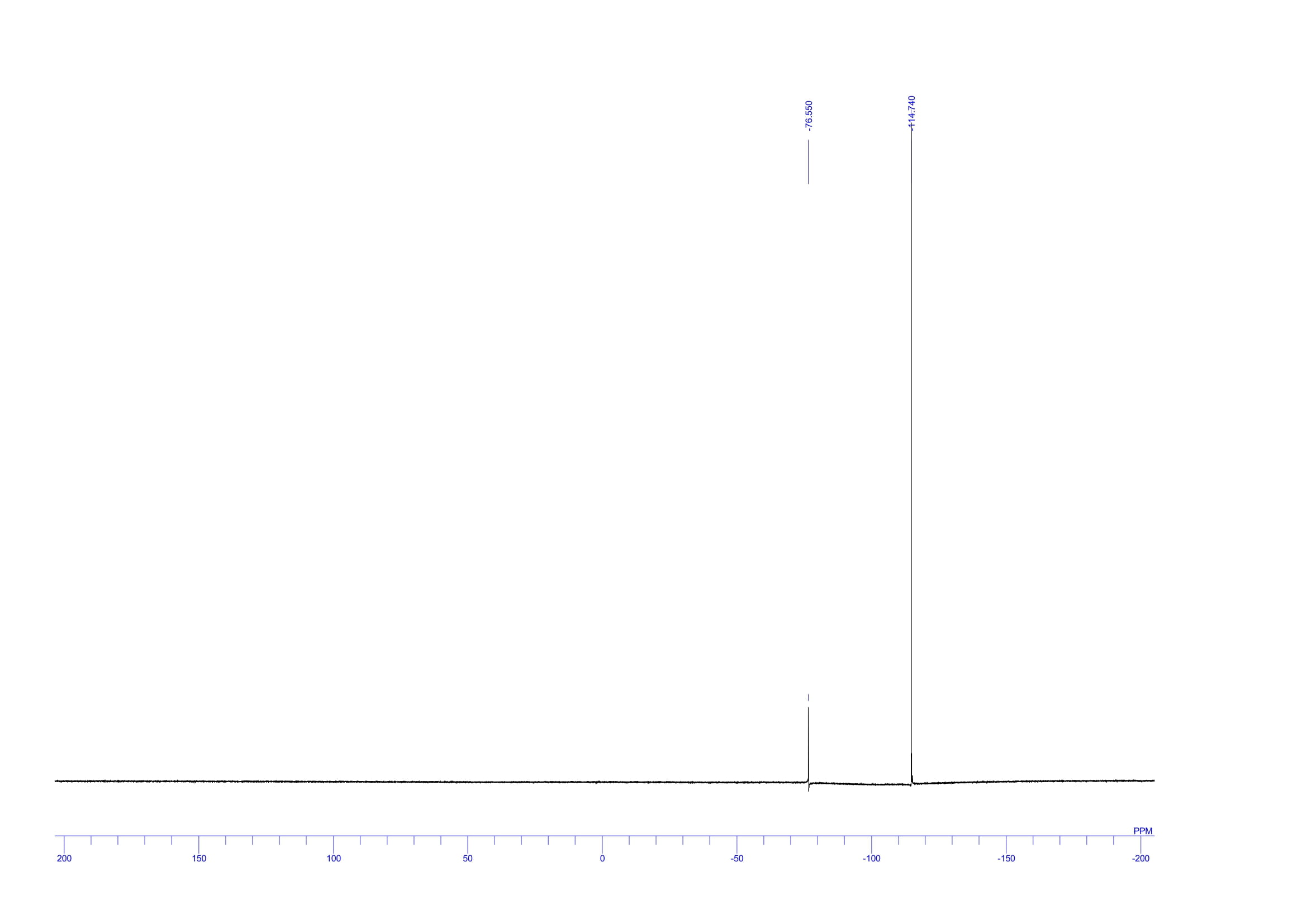
**
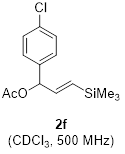
**
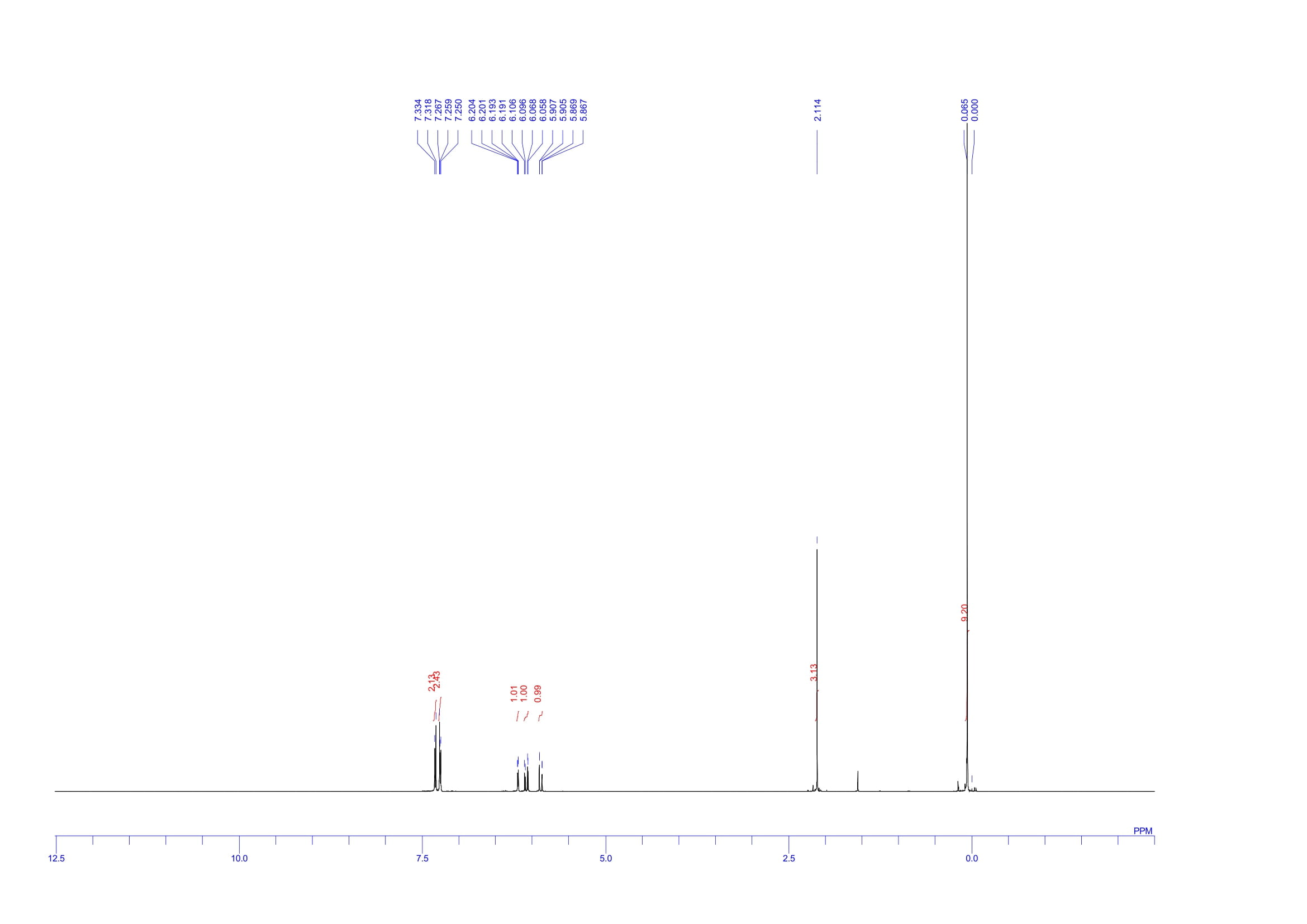
**


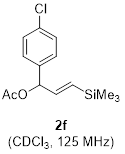
**
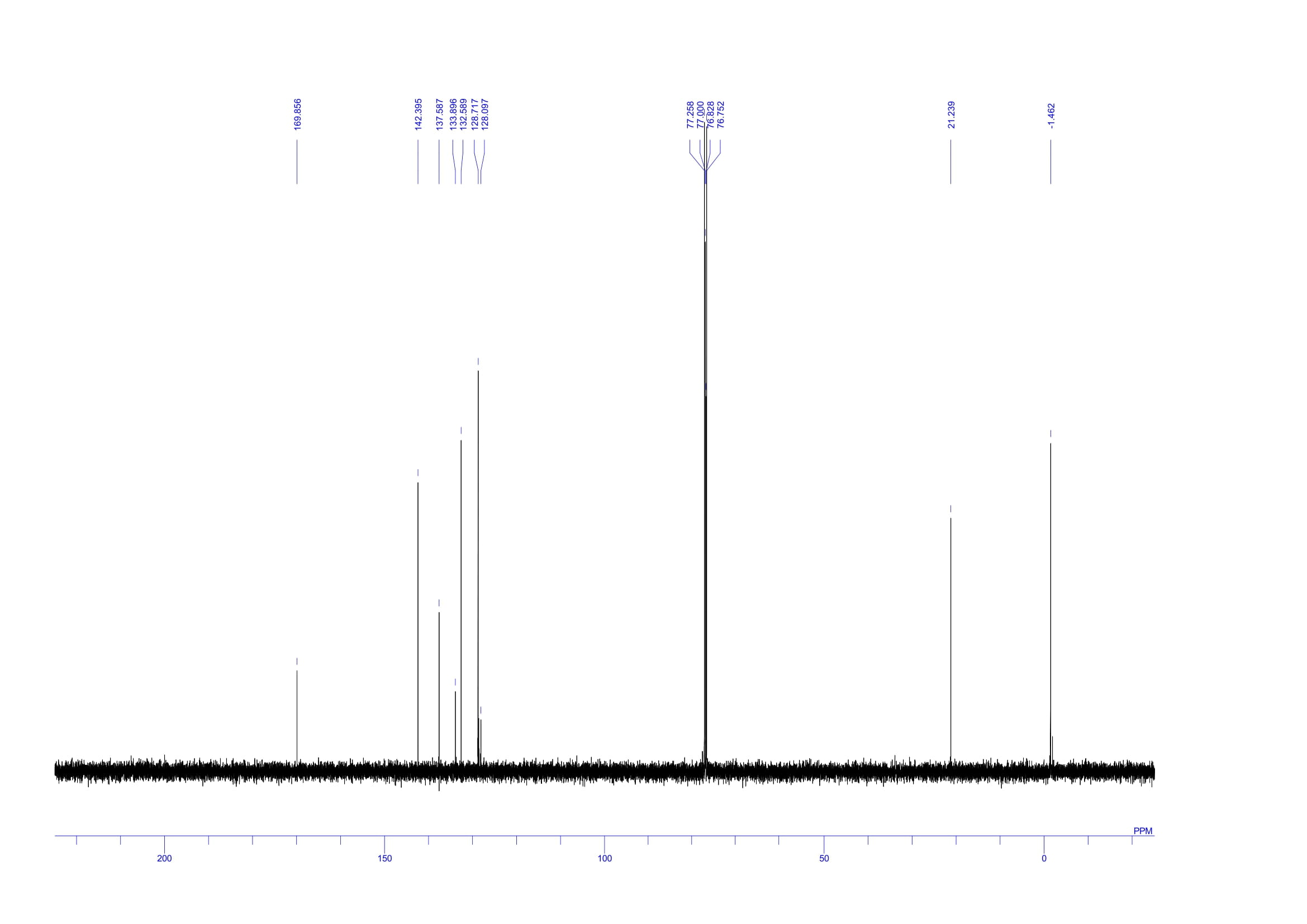
**


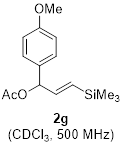
**
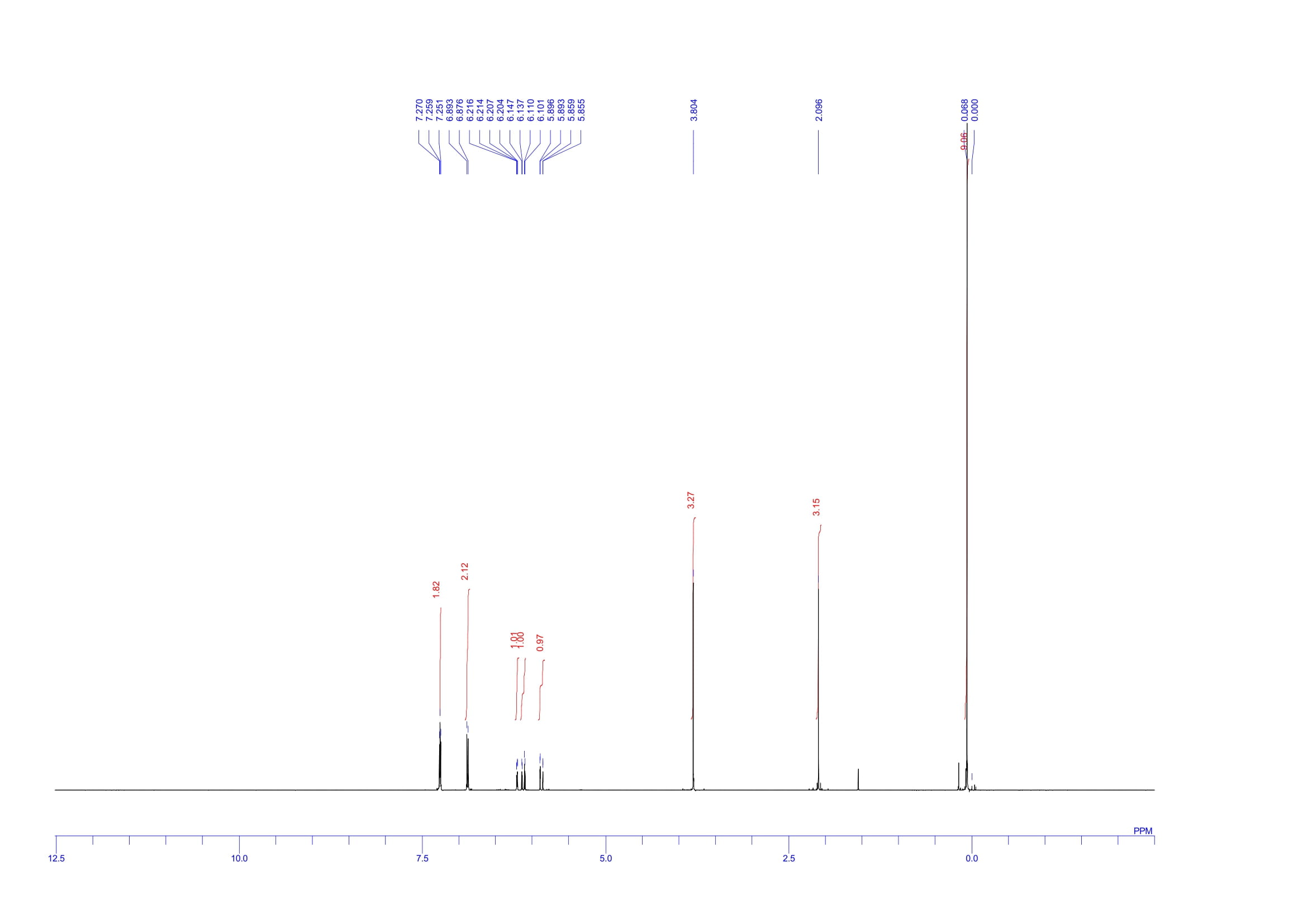
**


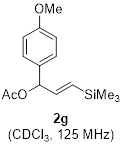
**
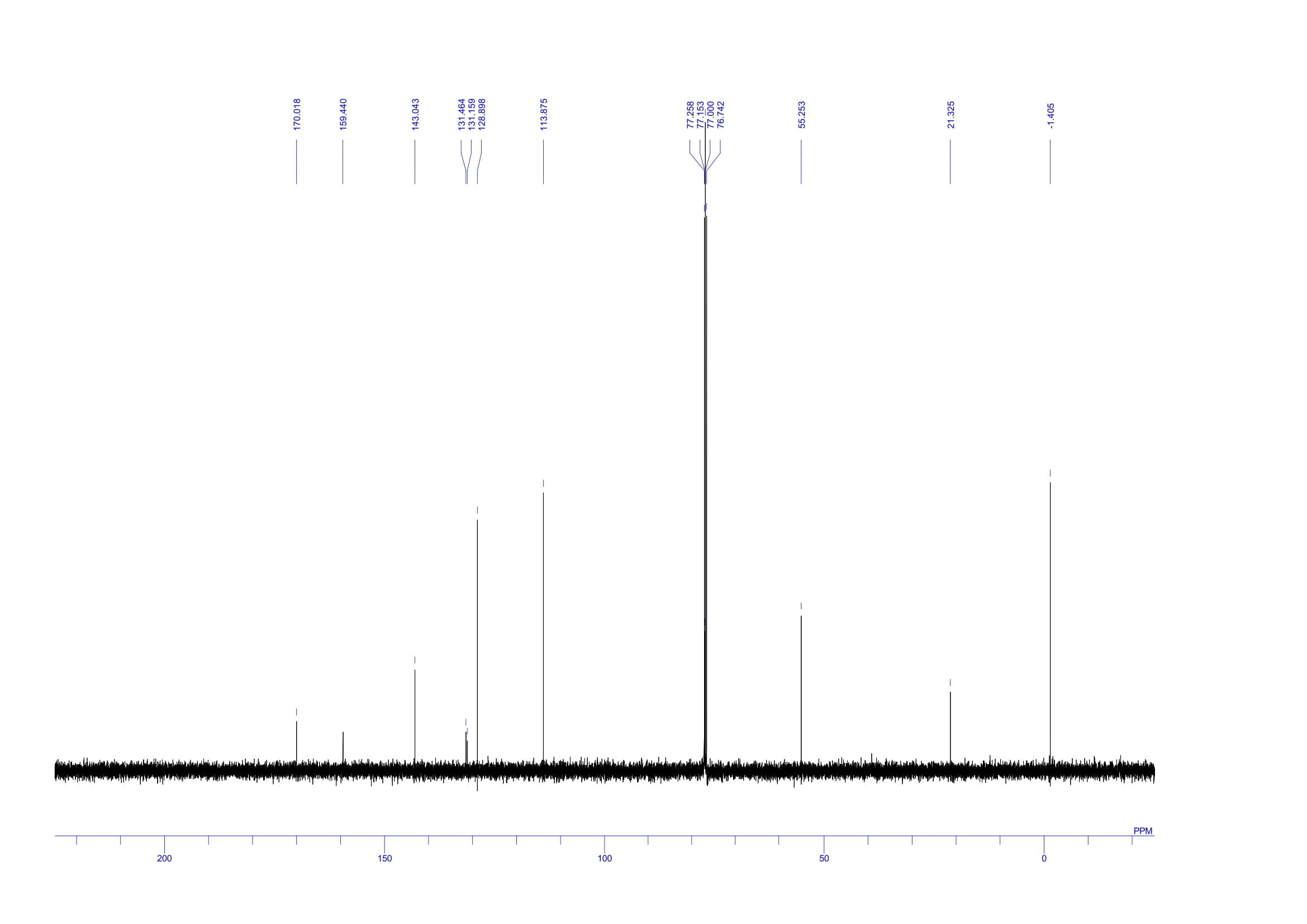
**


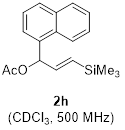
**
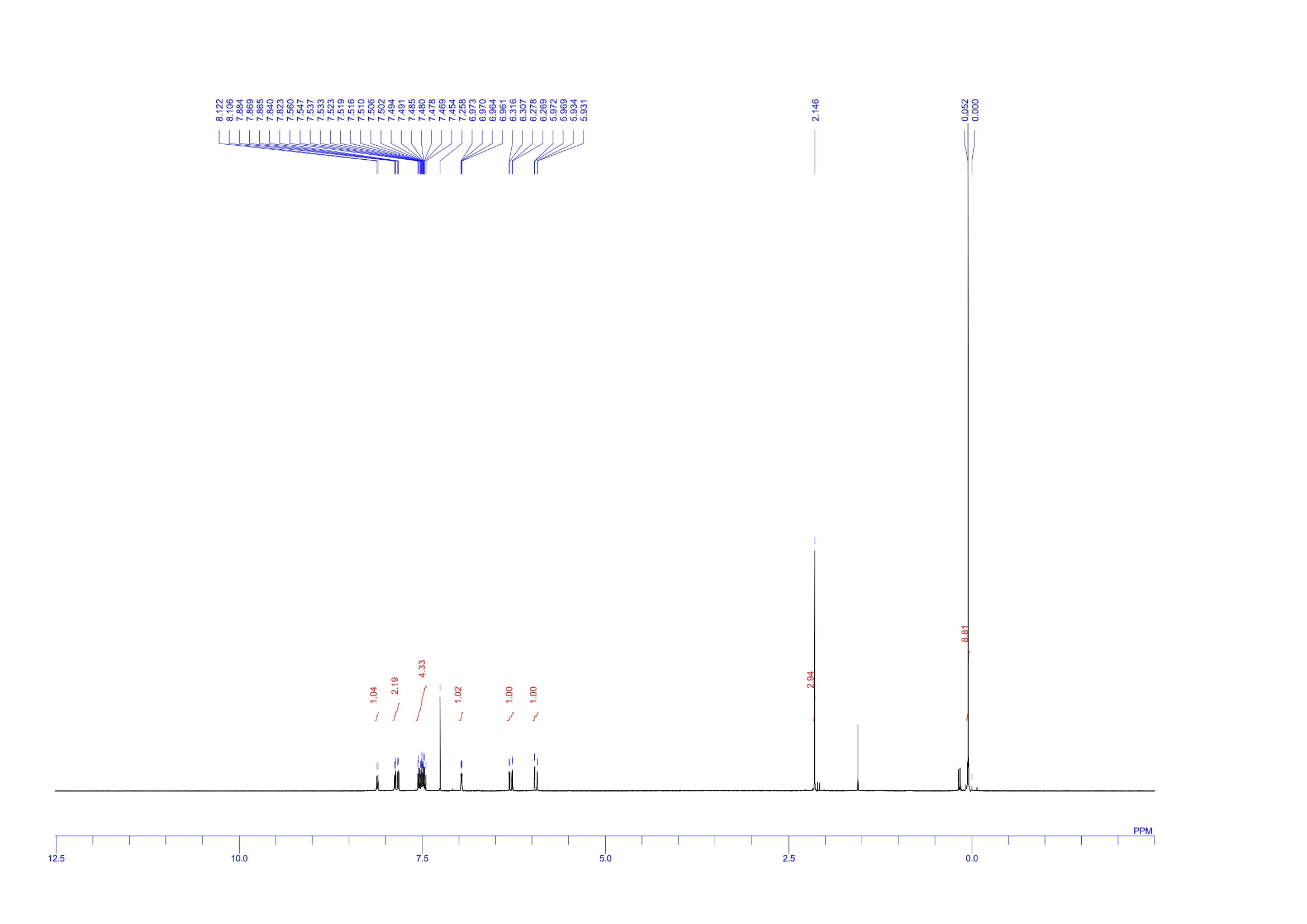
**


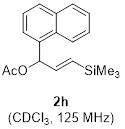
**
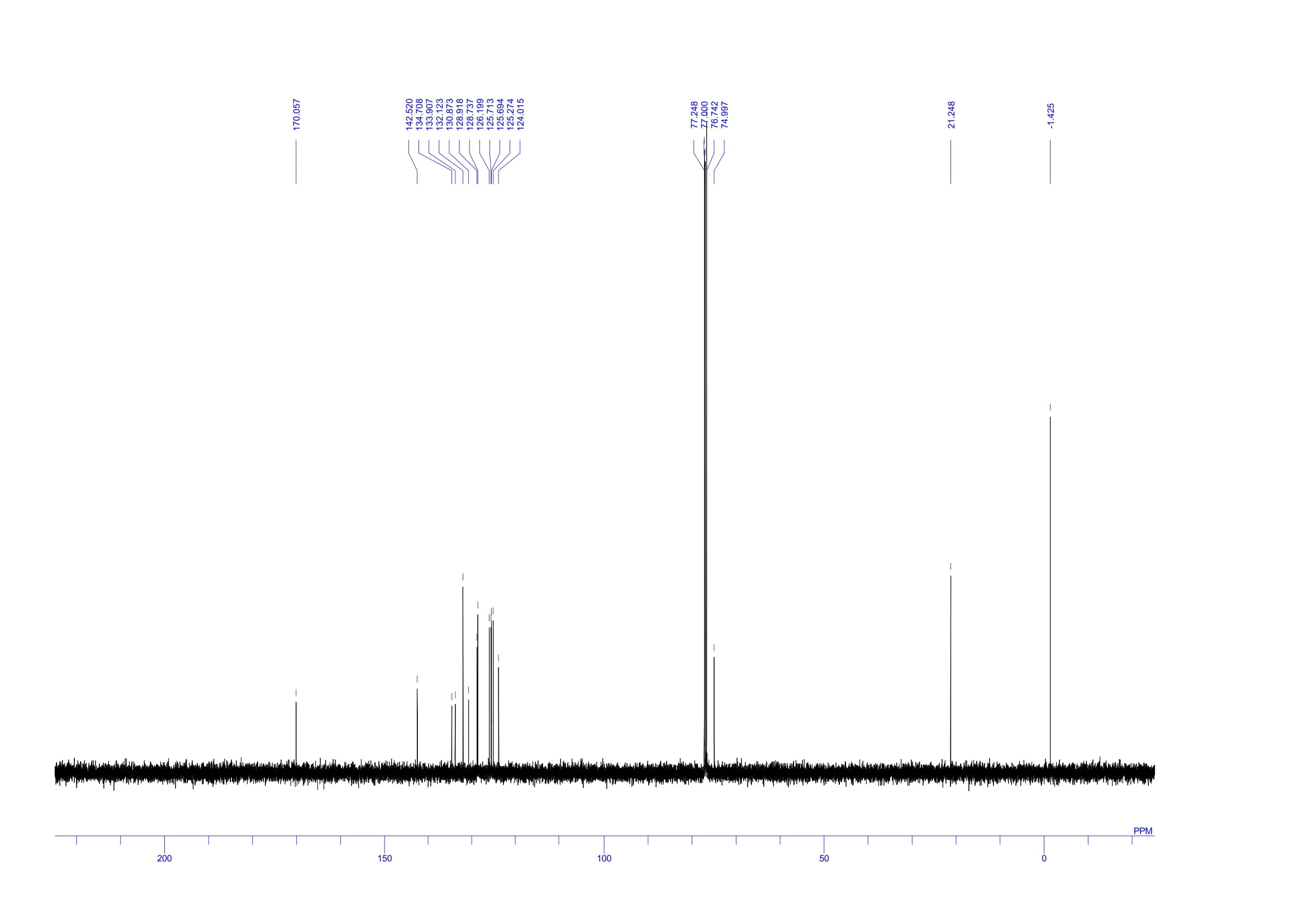
**


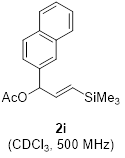
**
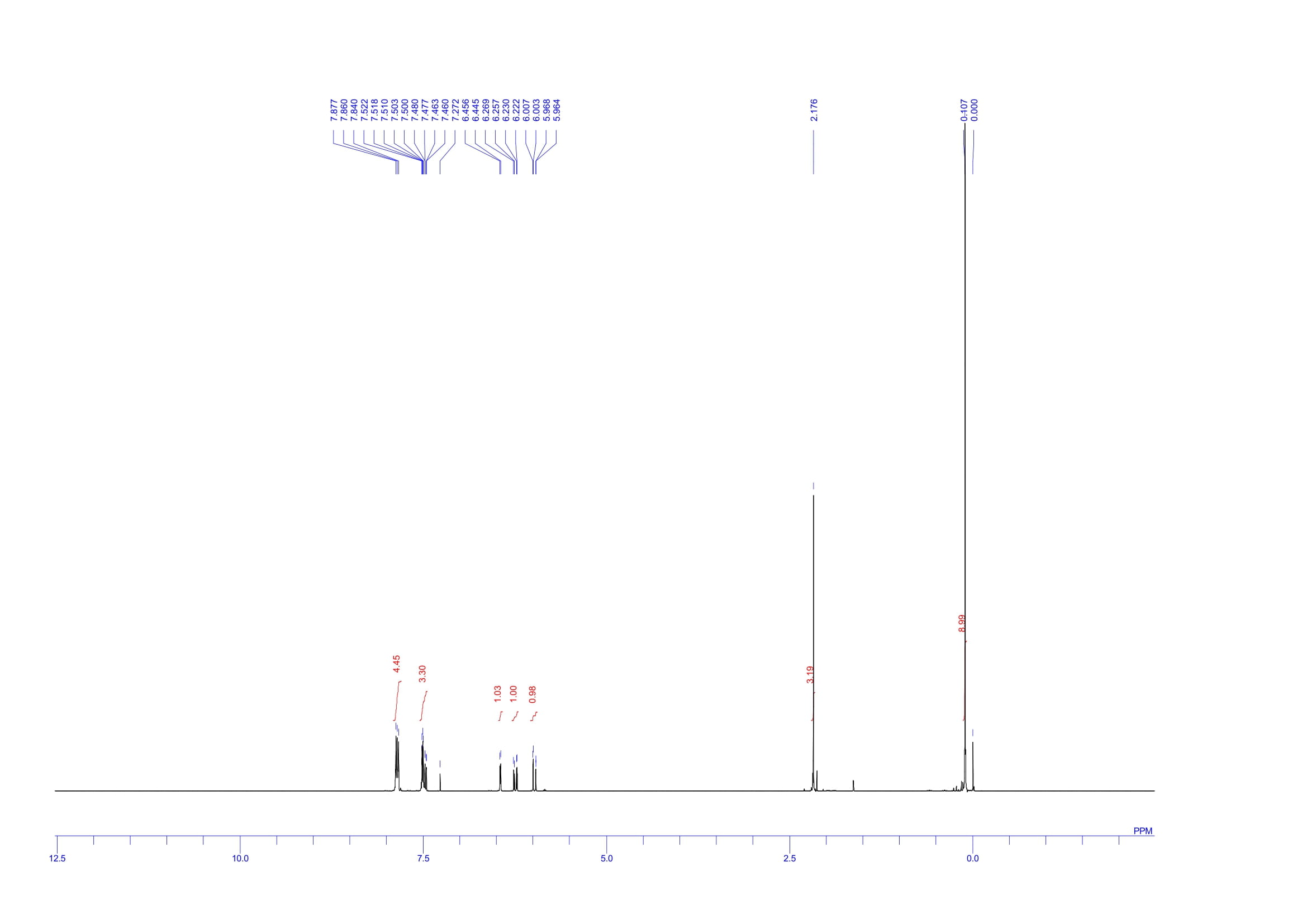
**


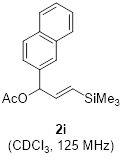
**
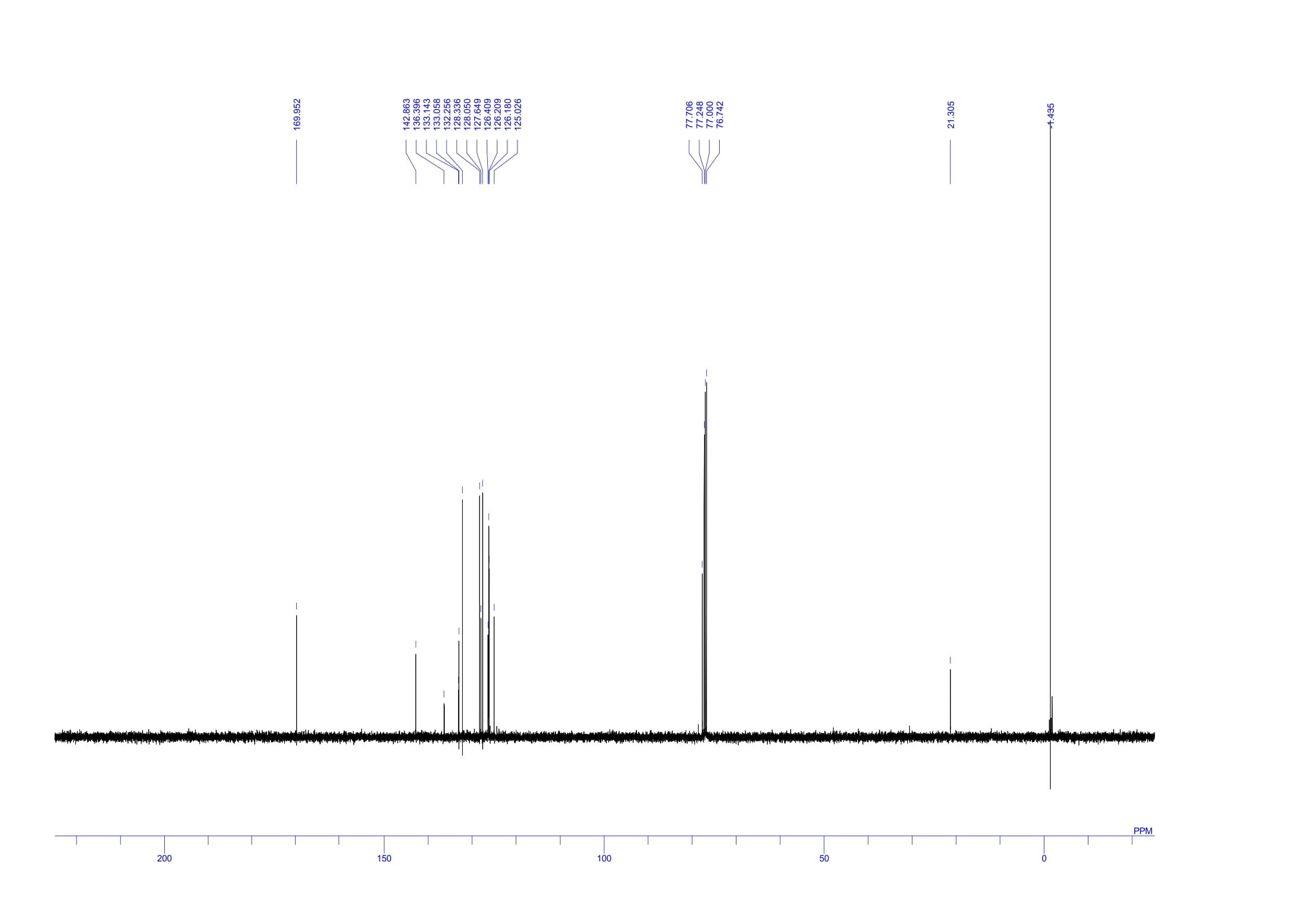
**


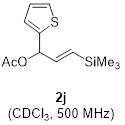
**
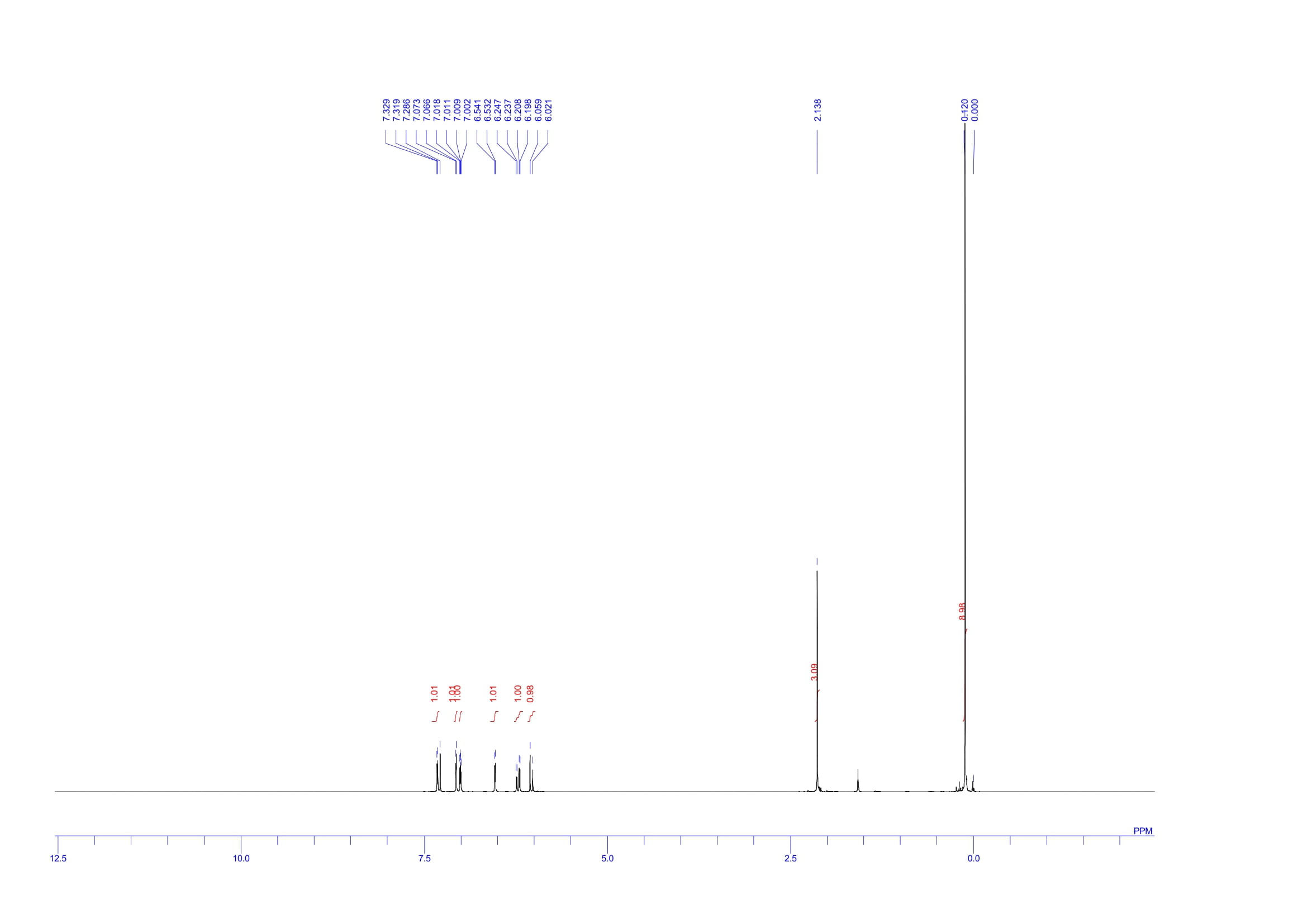
**


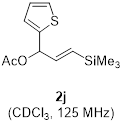
**
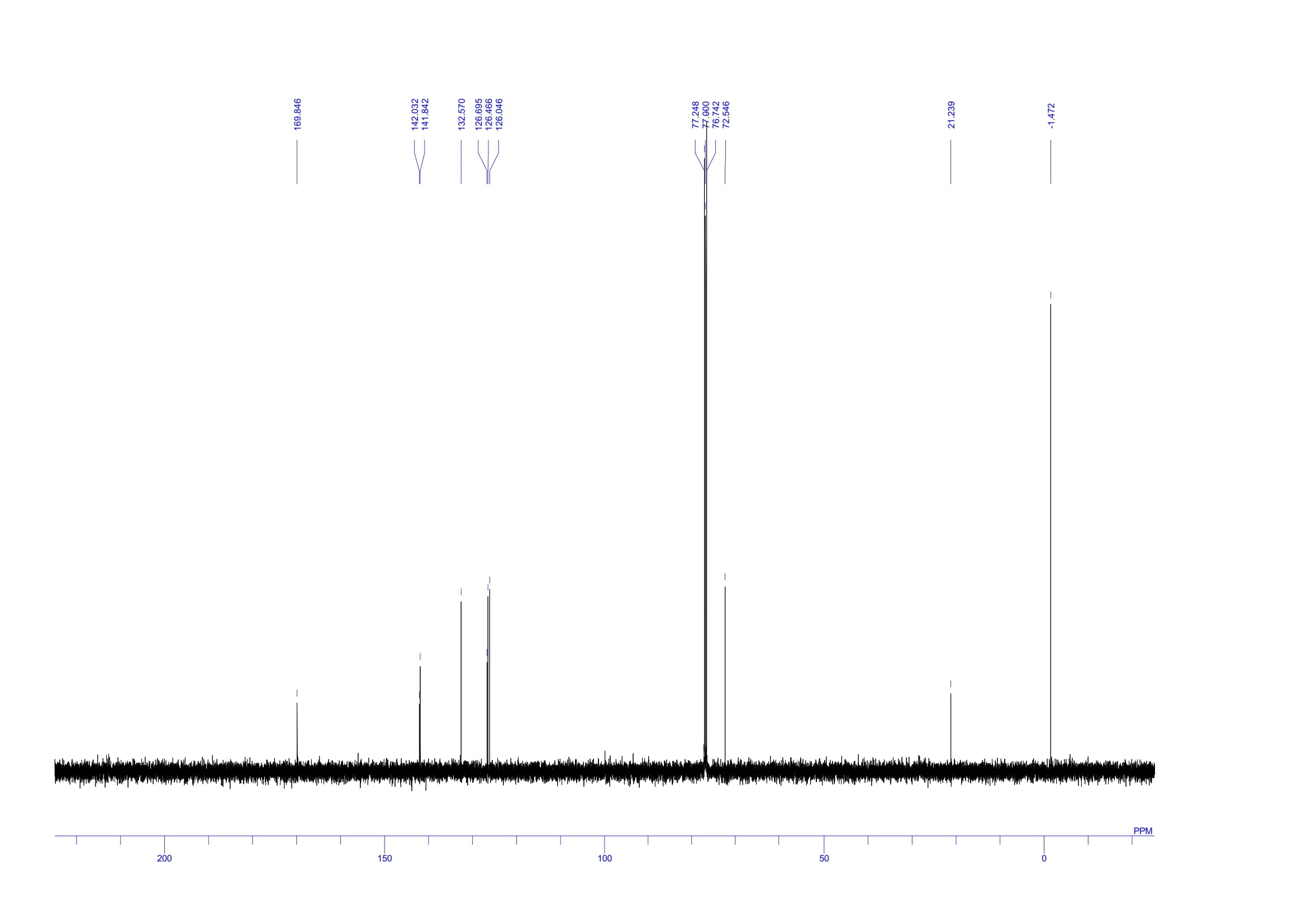
**


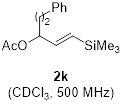
**
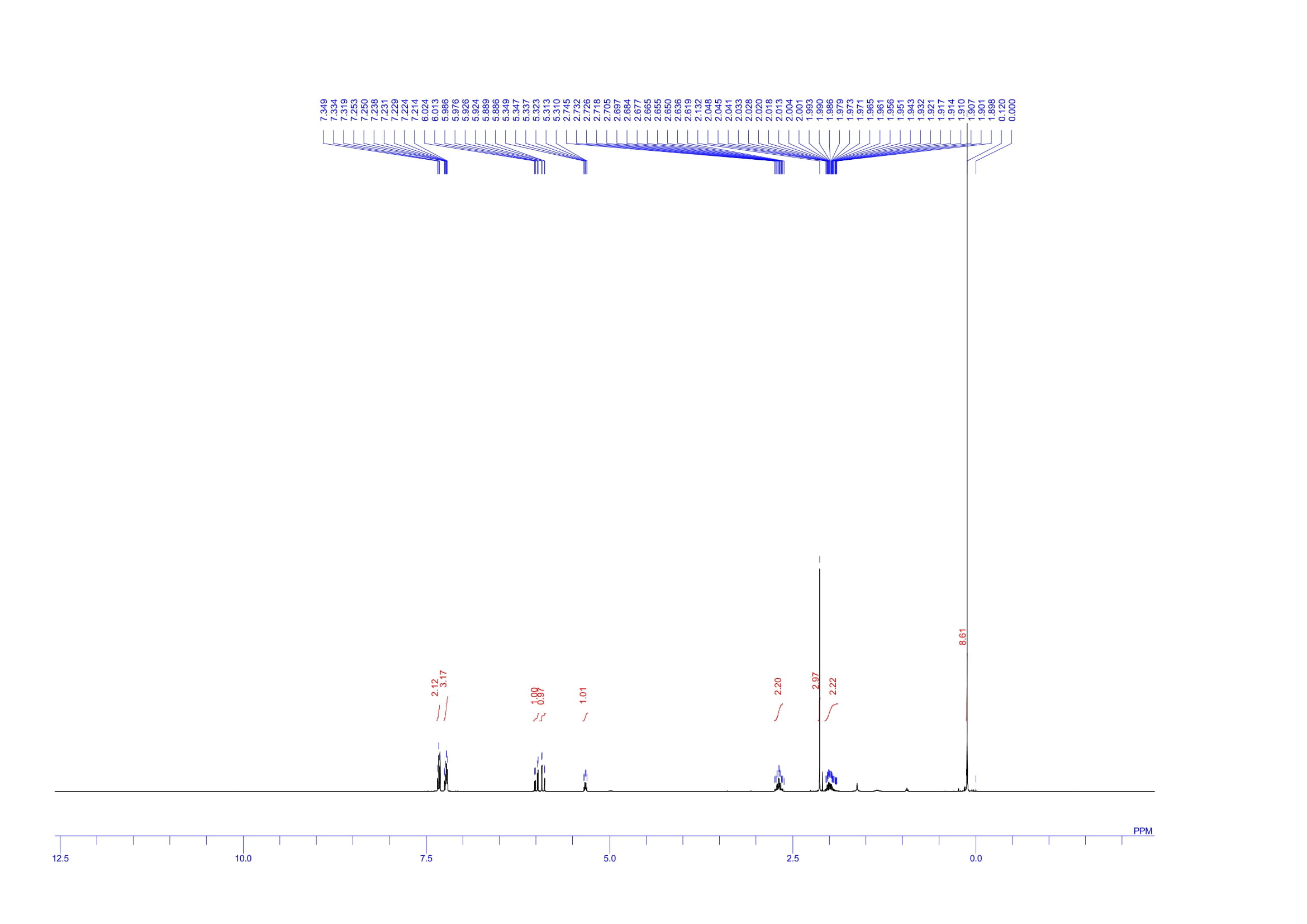
**


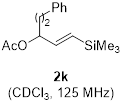
**
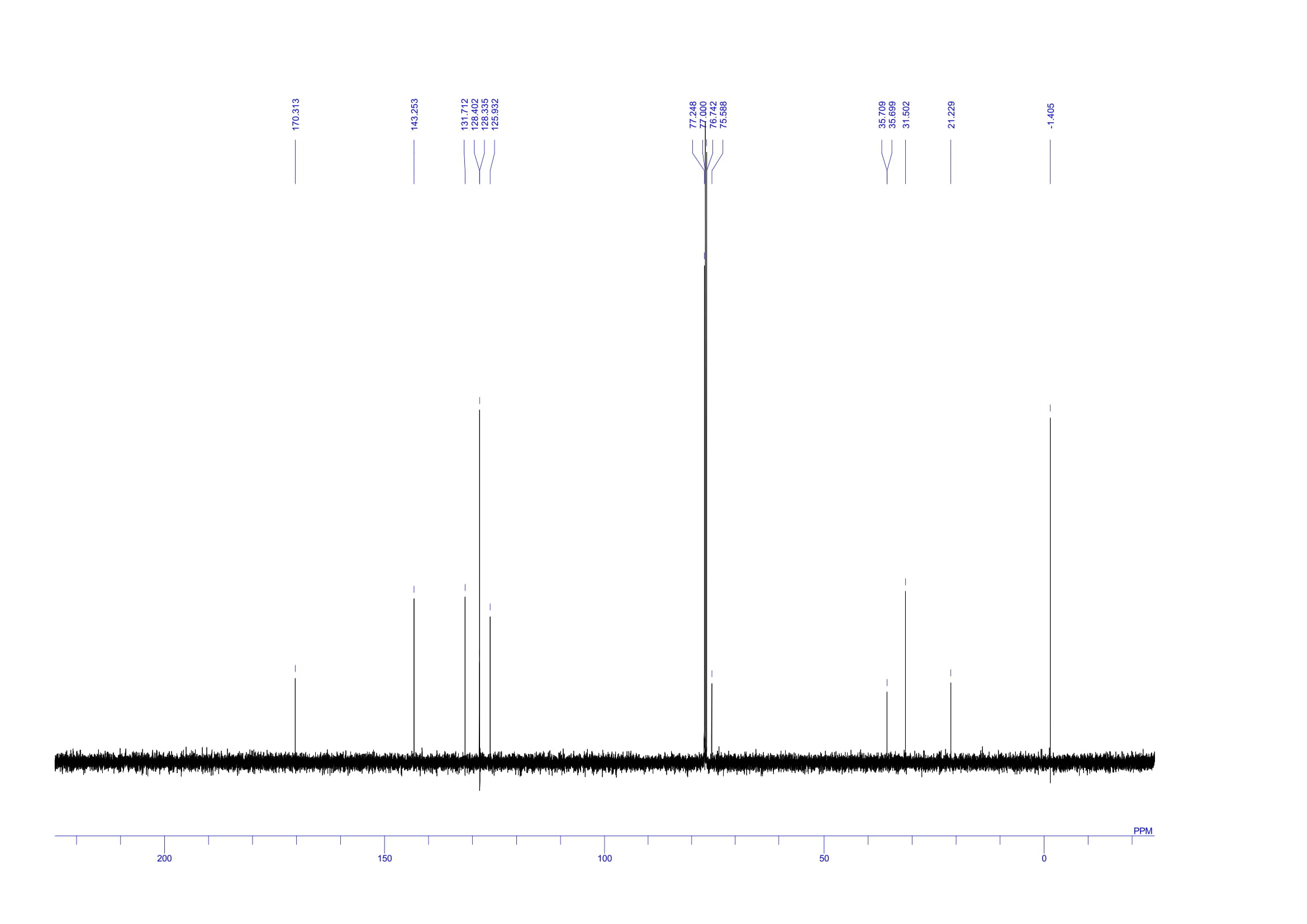
**


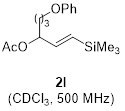
**
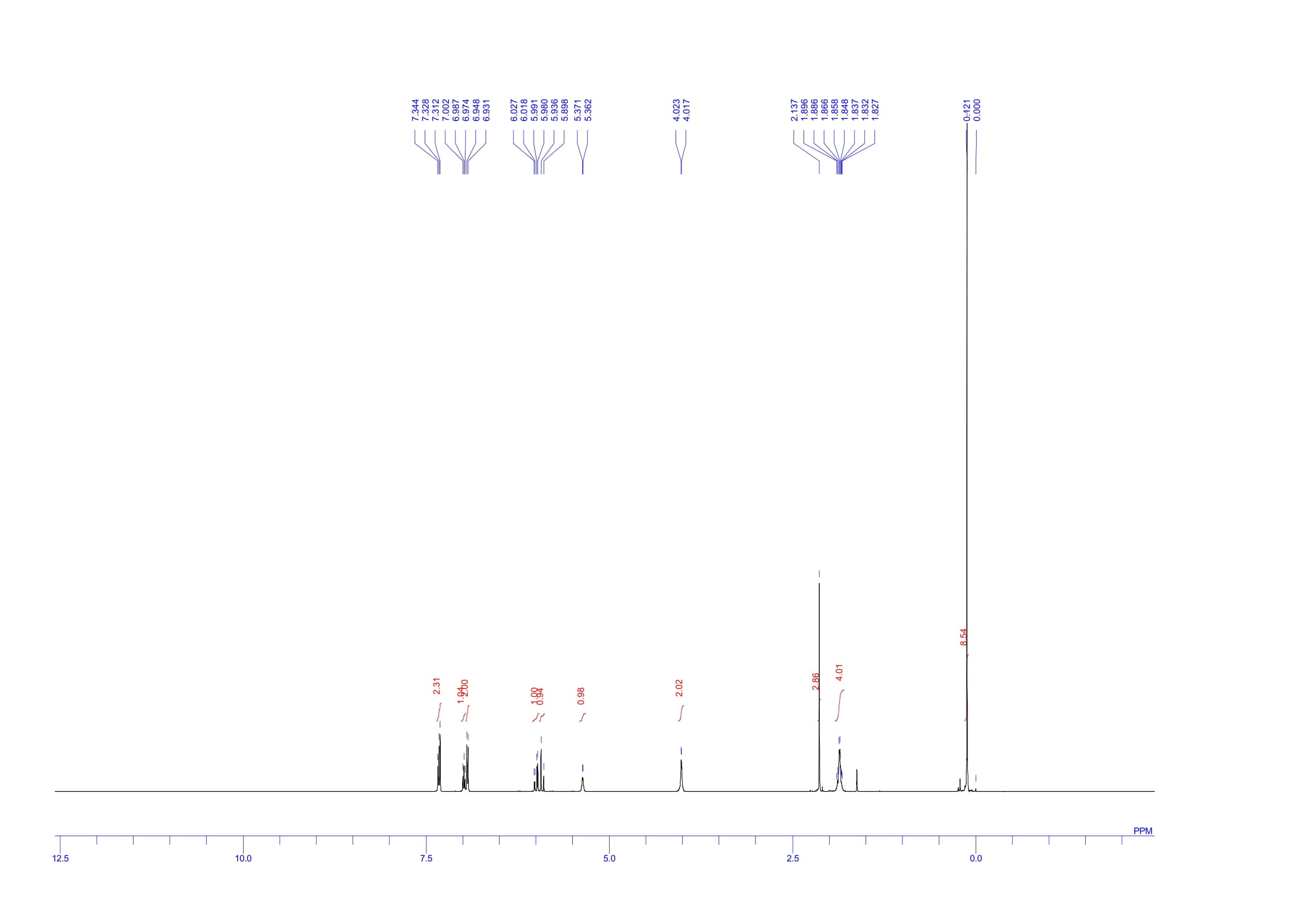
**


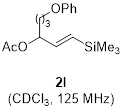
**
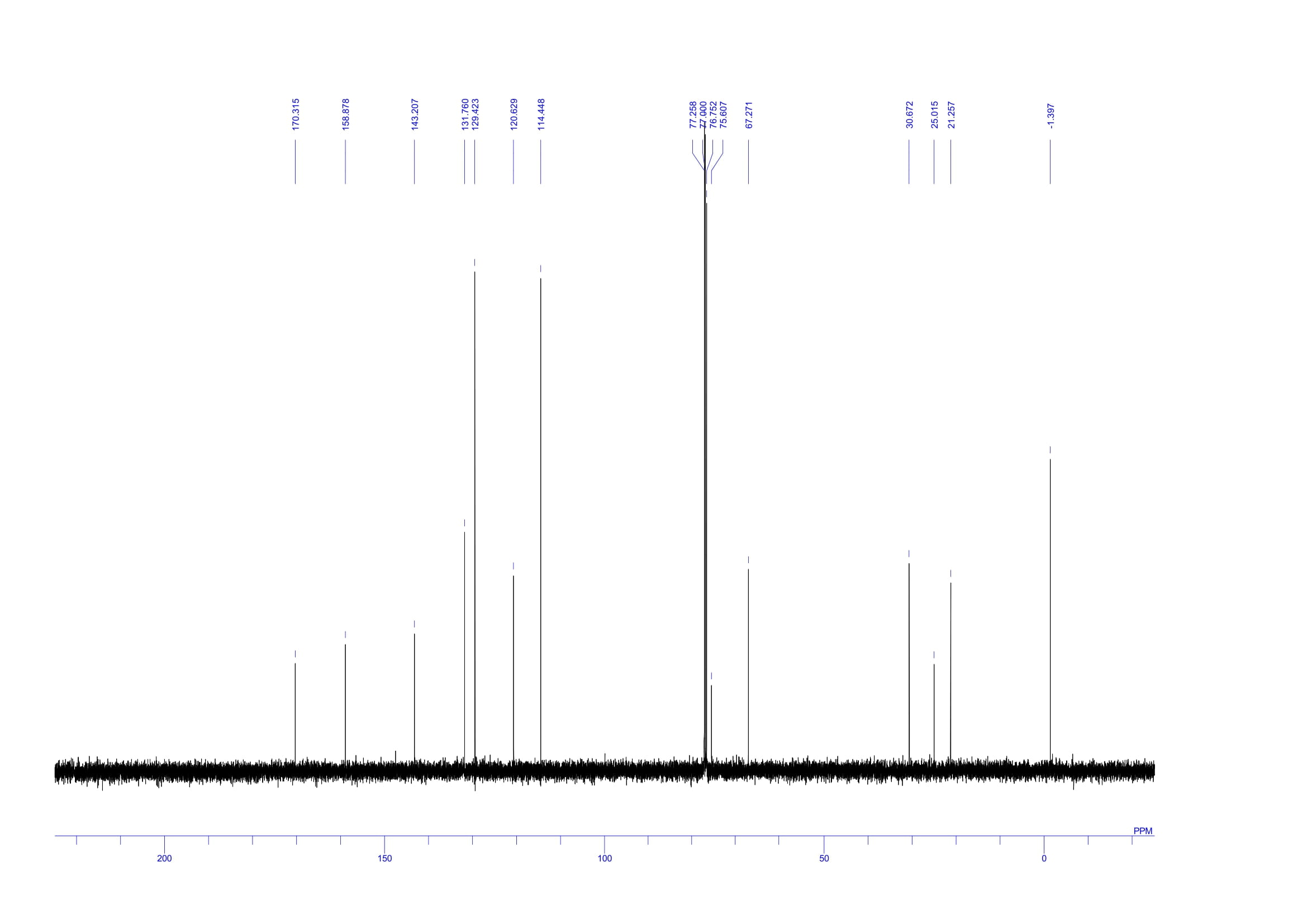
**
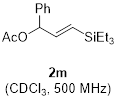
**
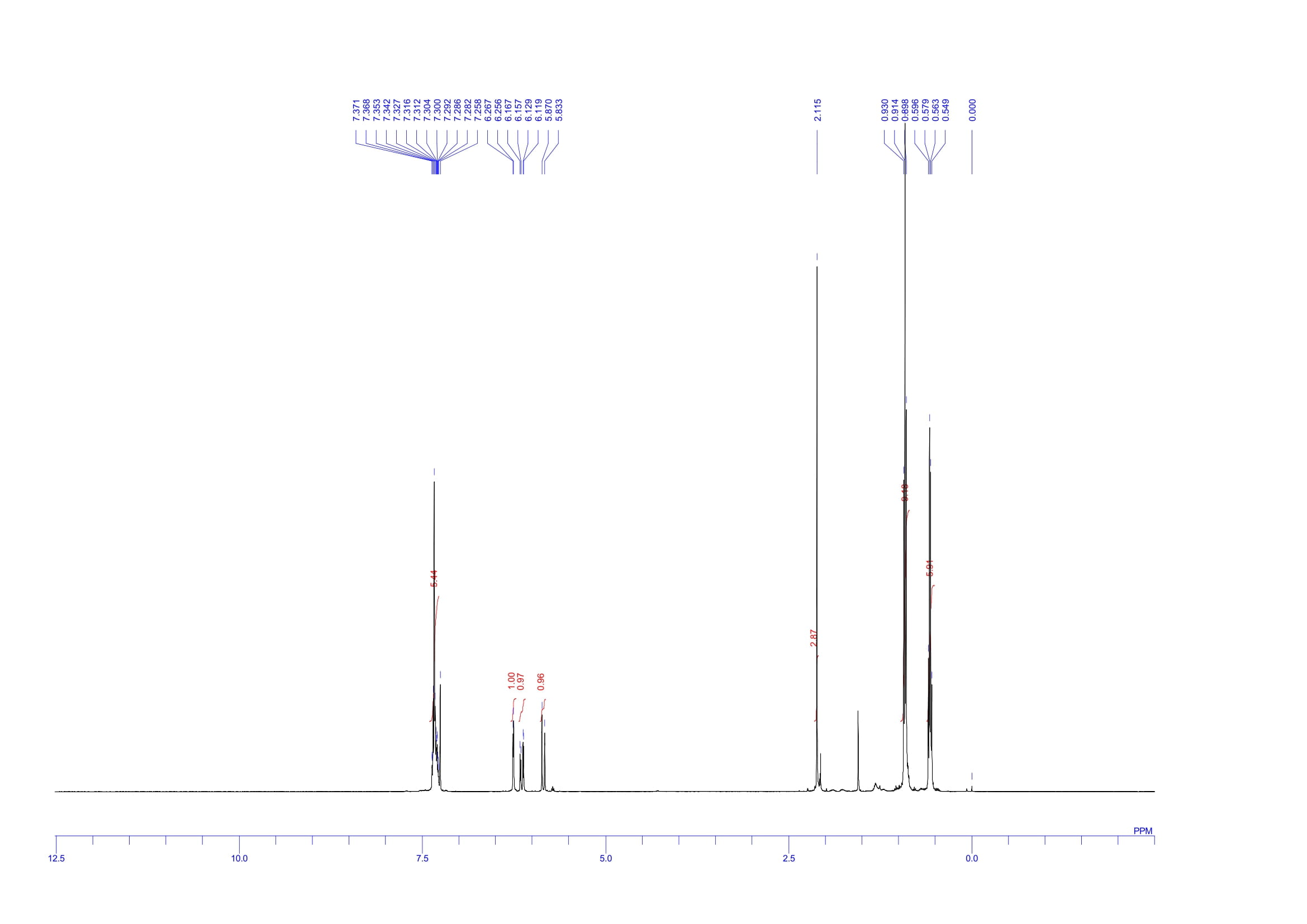
**


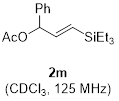
**
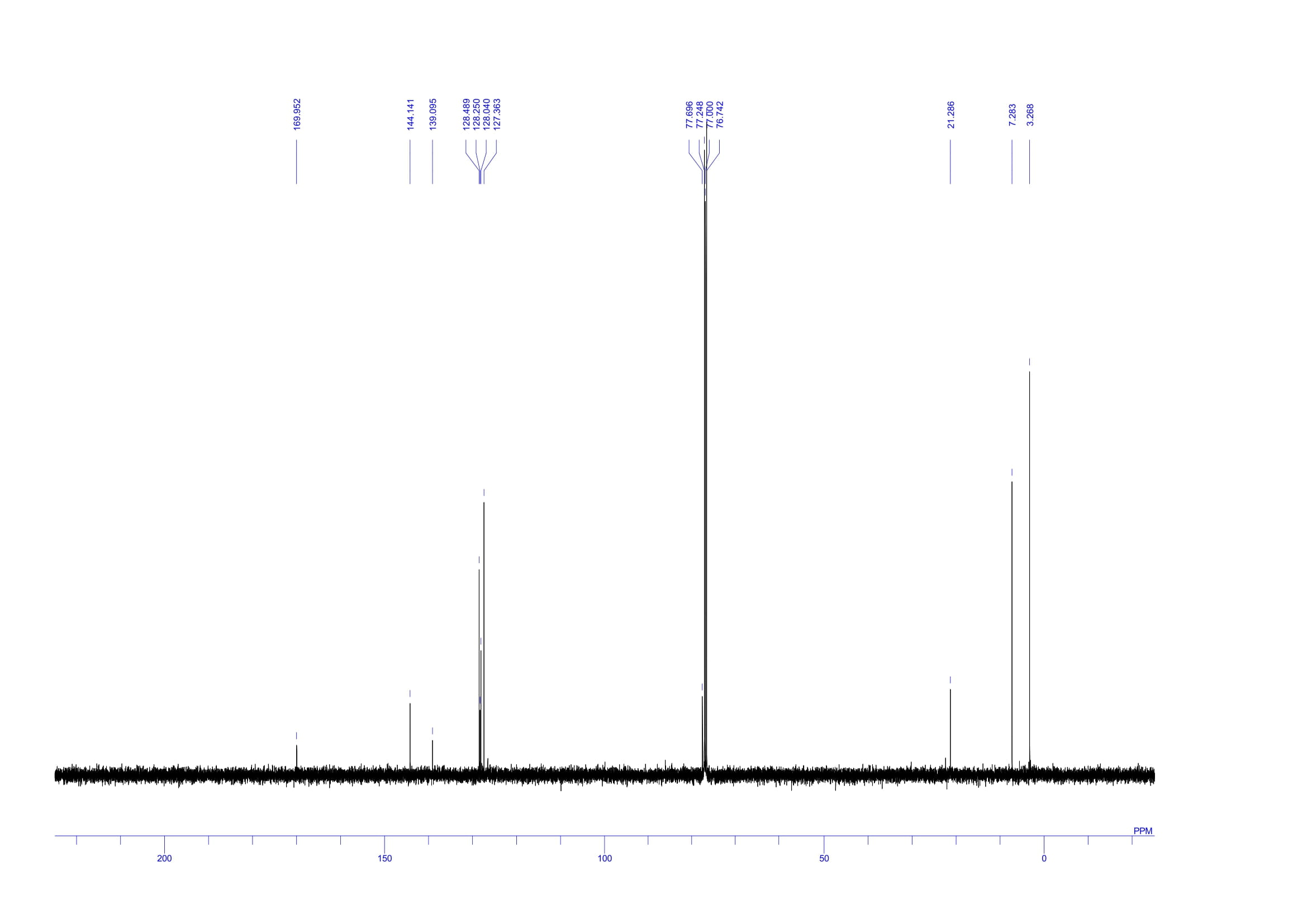
**


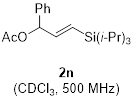
**
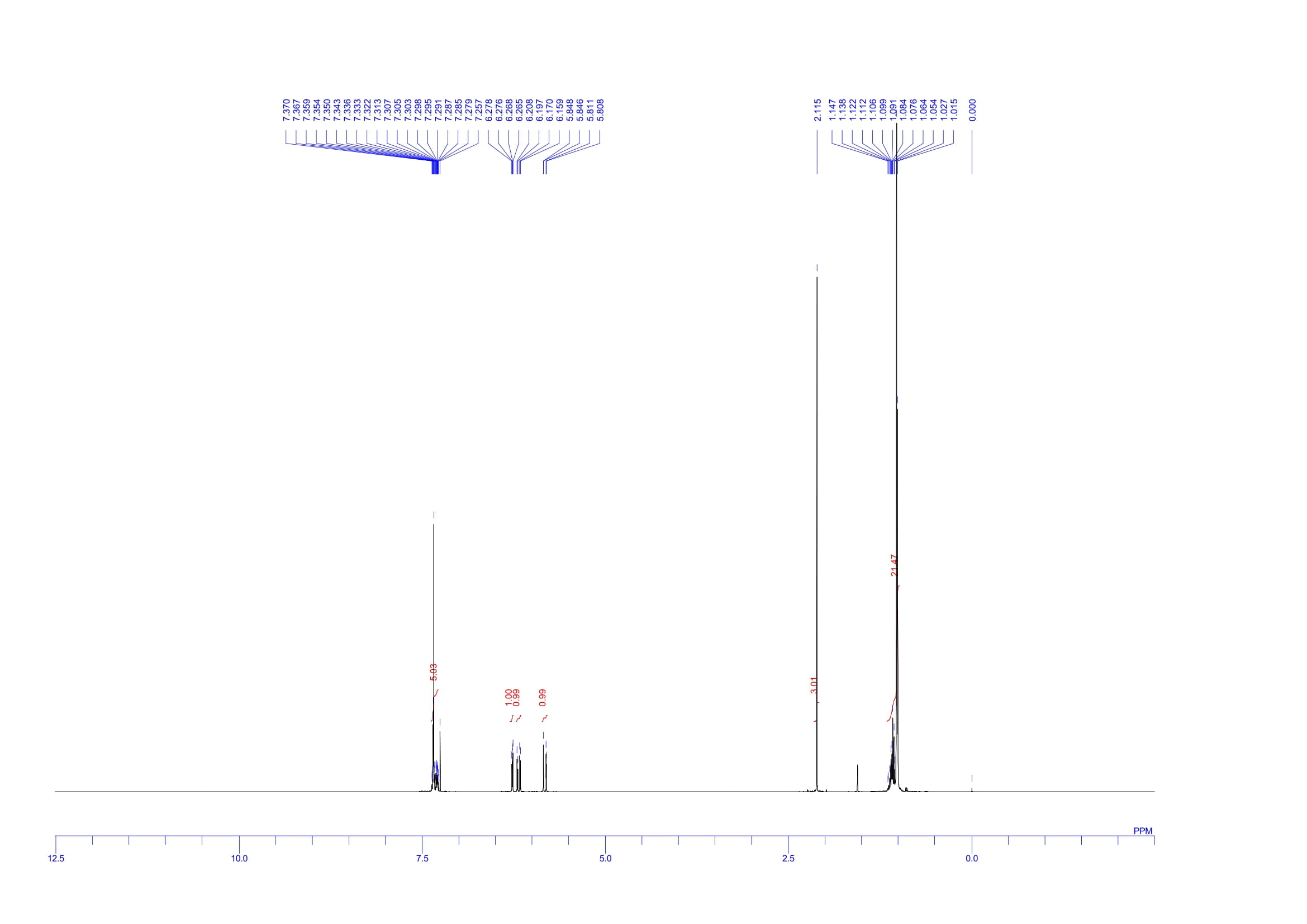
**


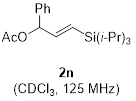
**
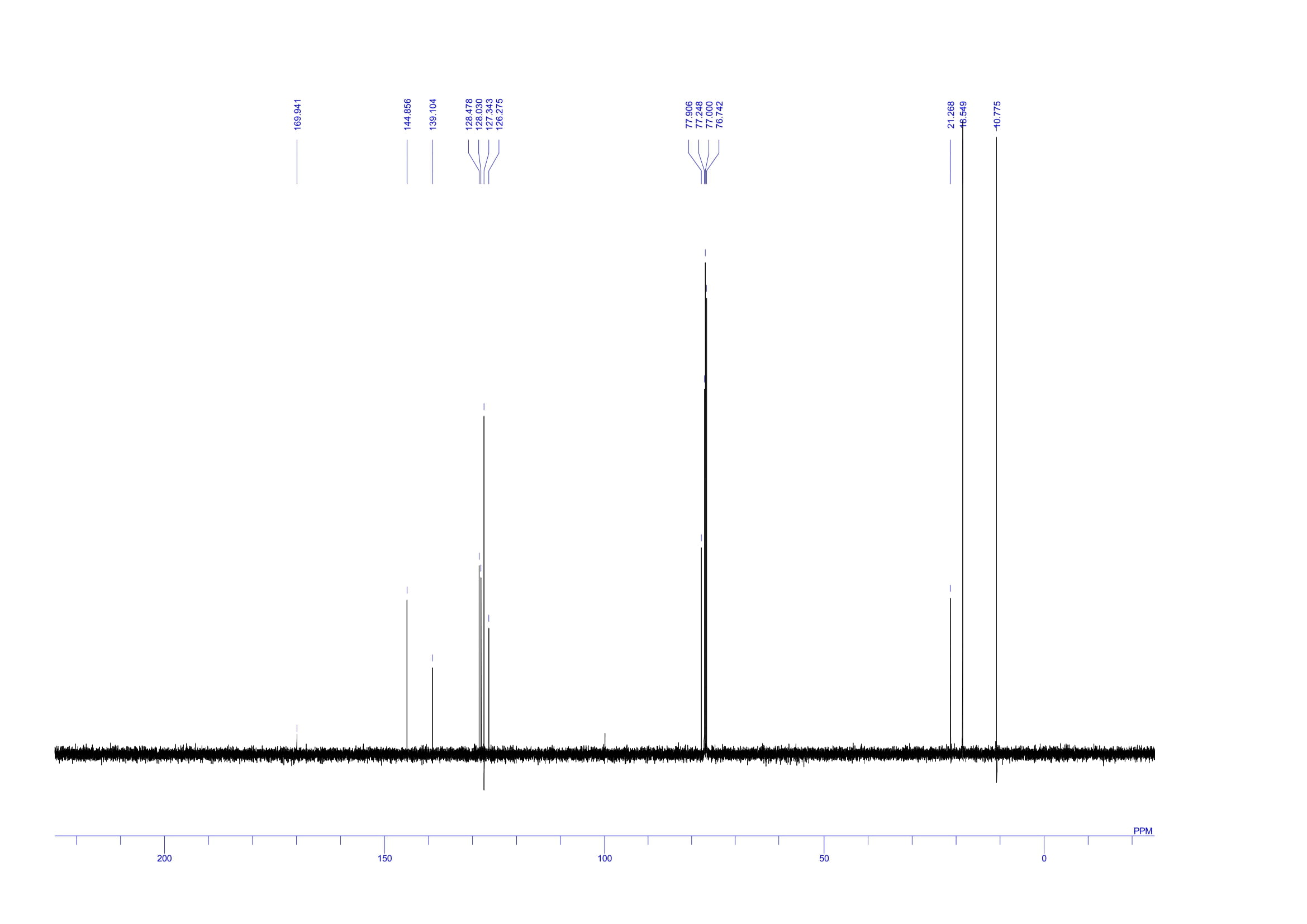
**


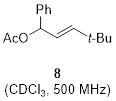
**
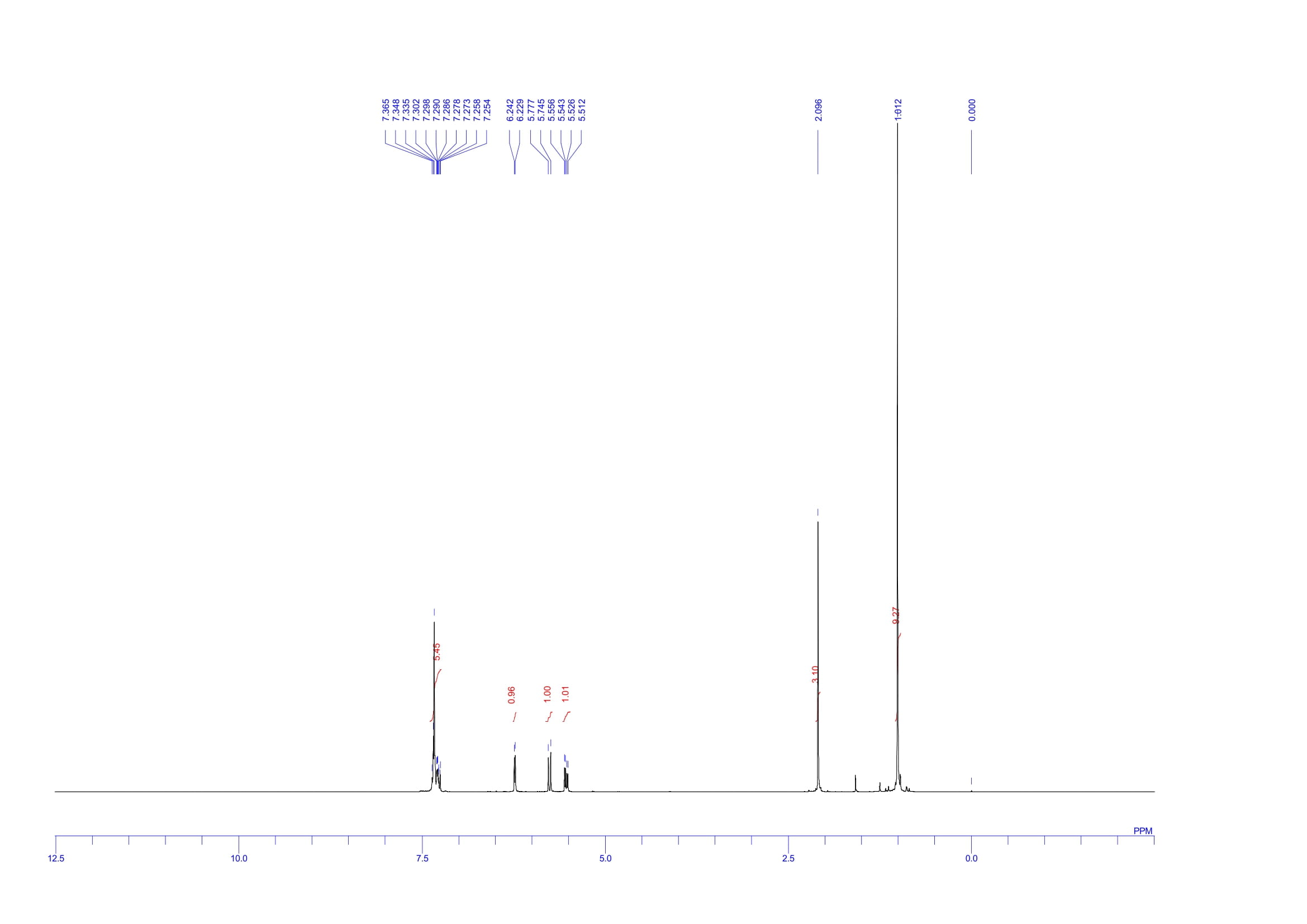
**


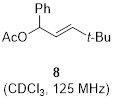
**
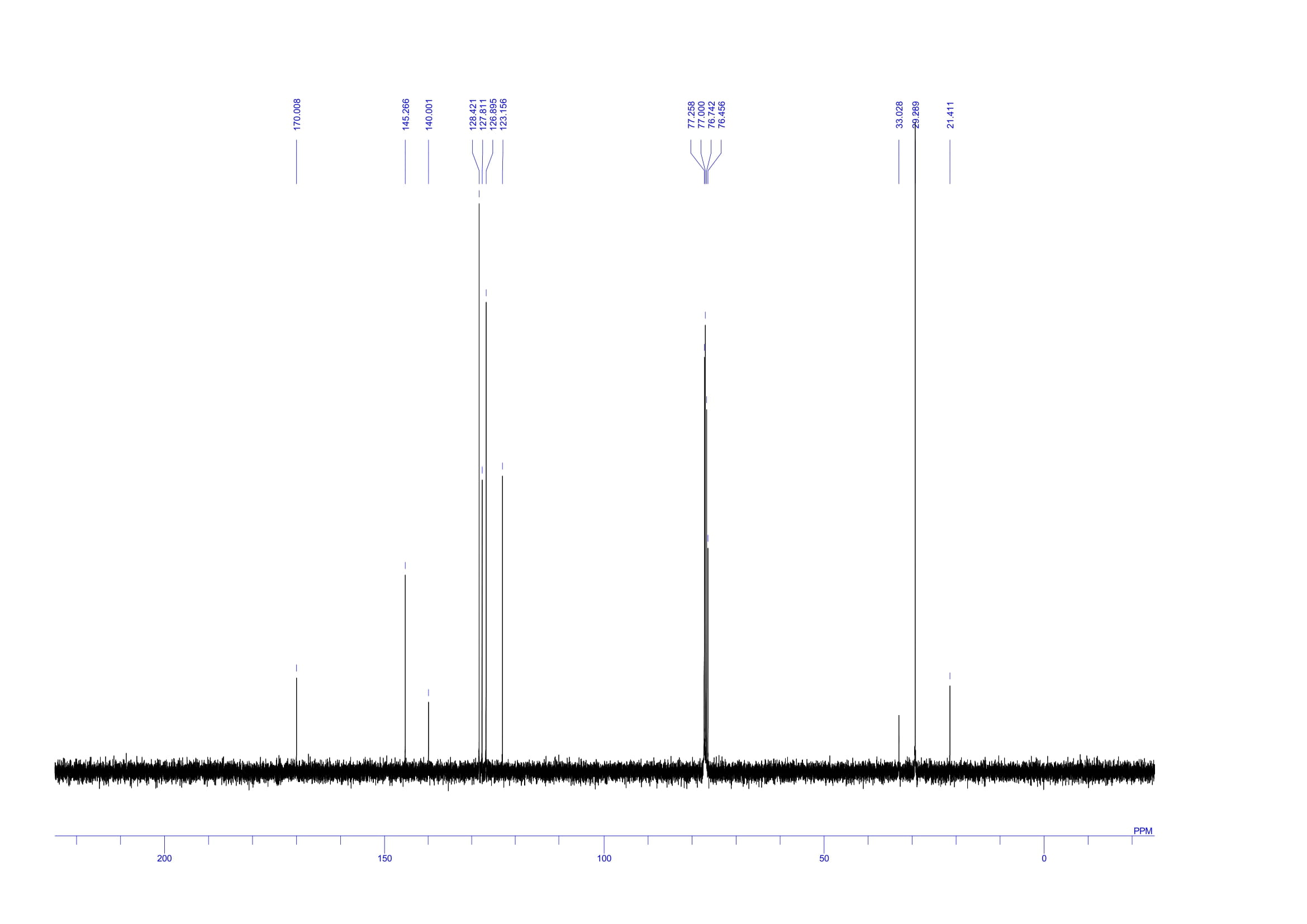
**


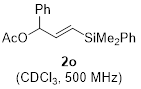
**
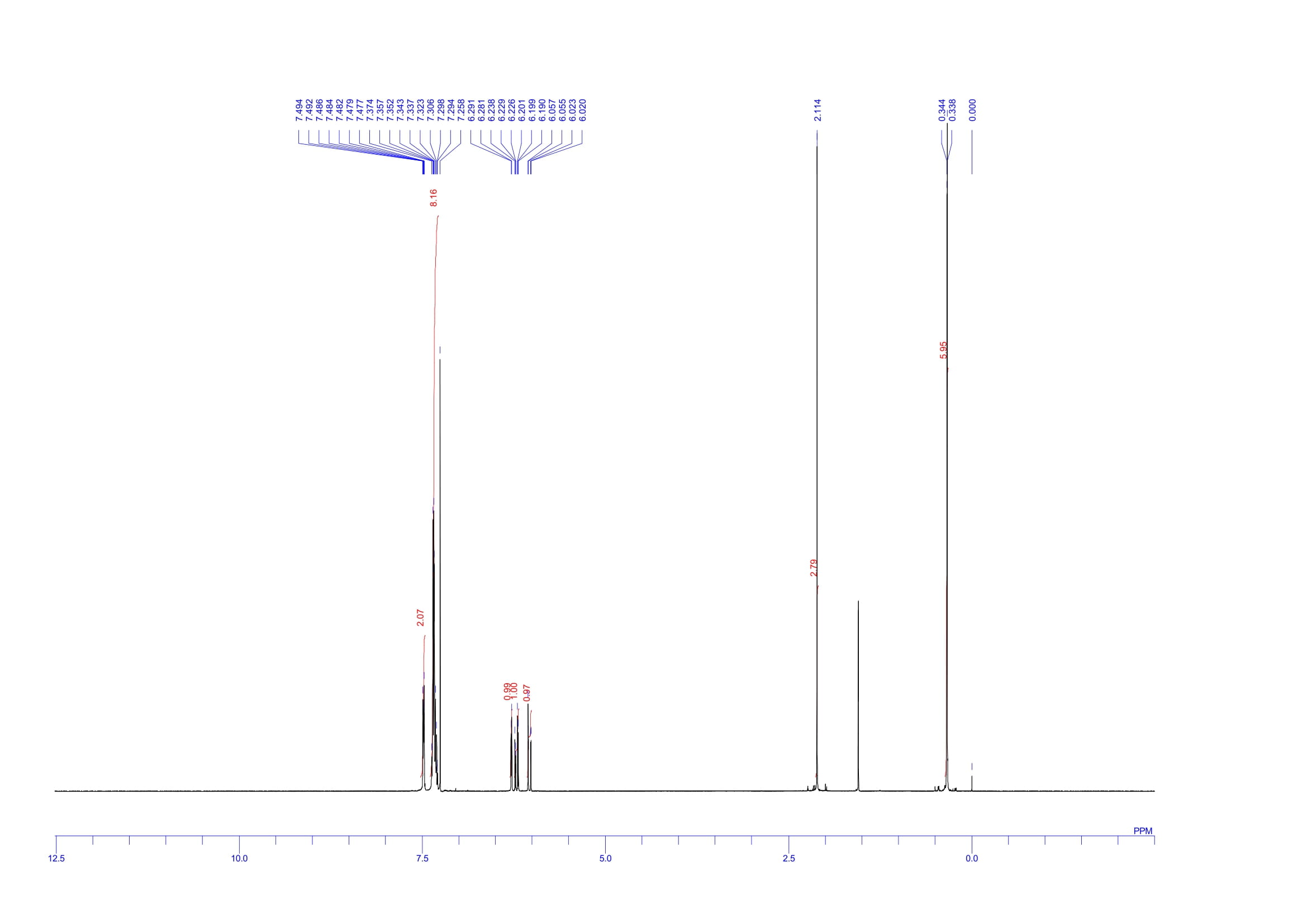
**


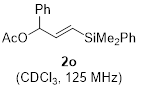
**
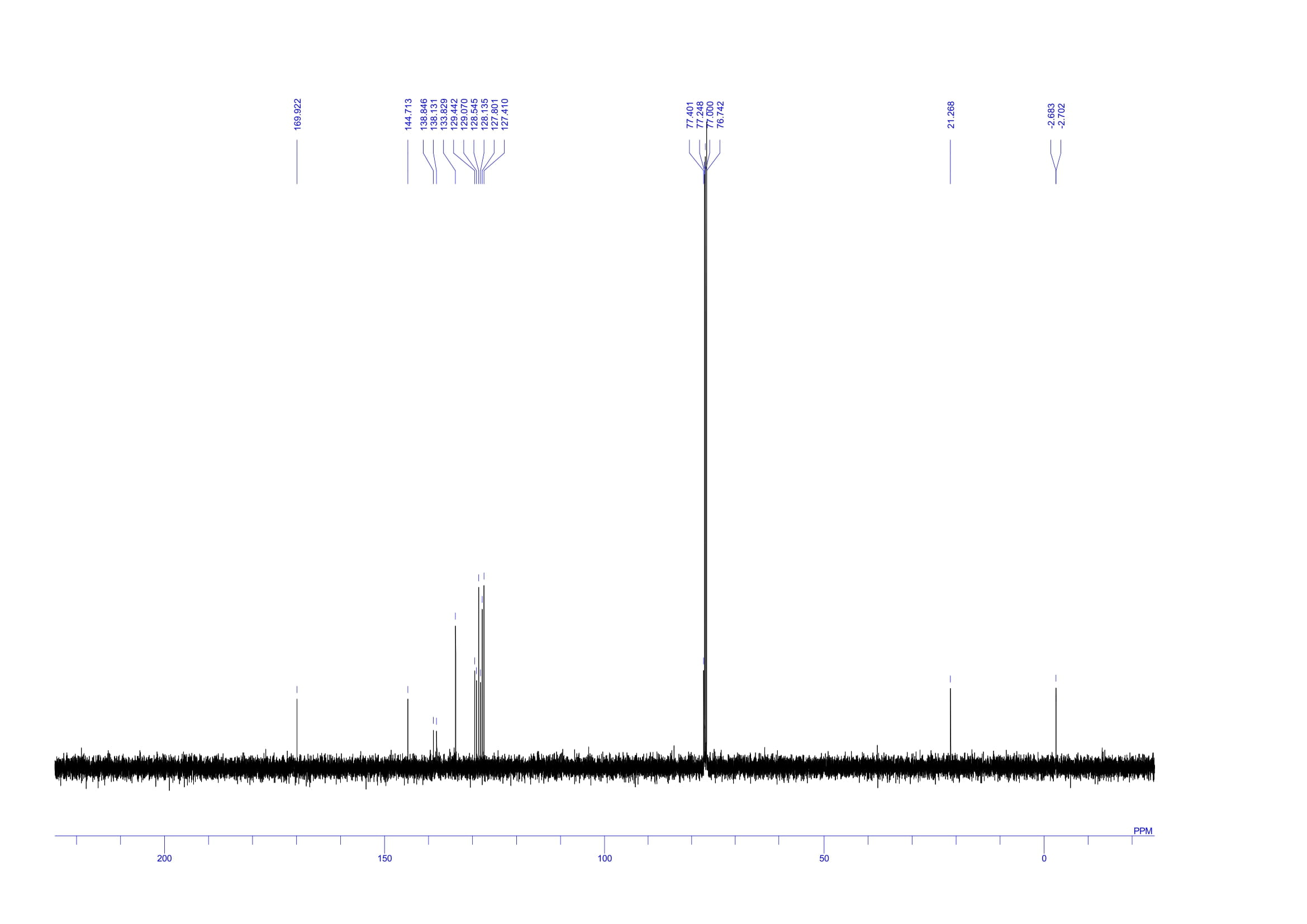
**

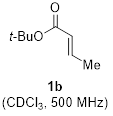
**
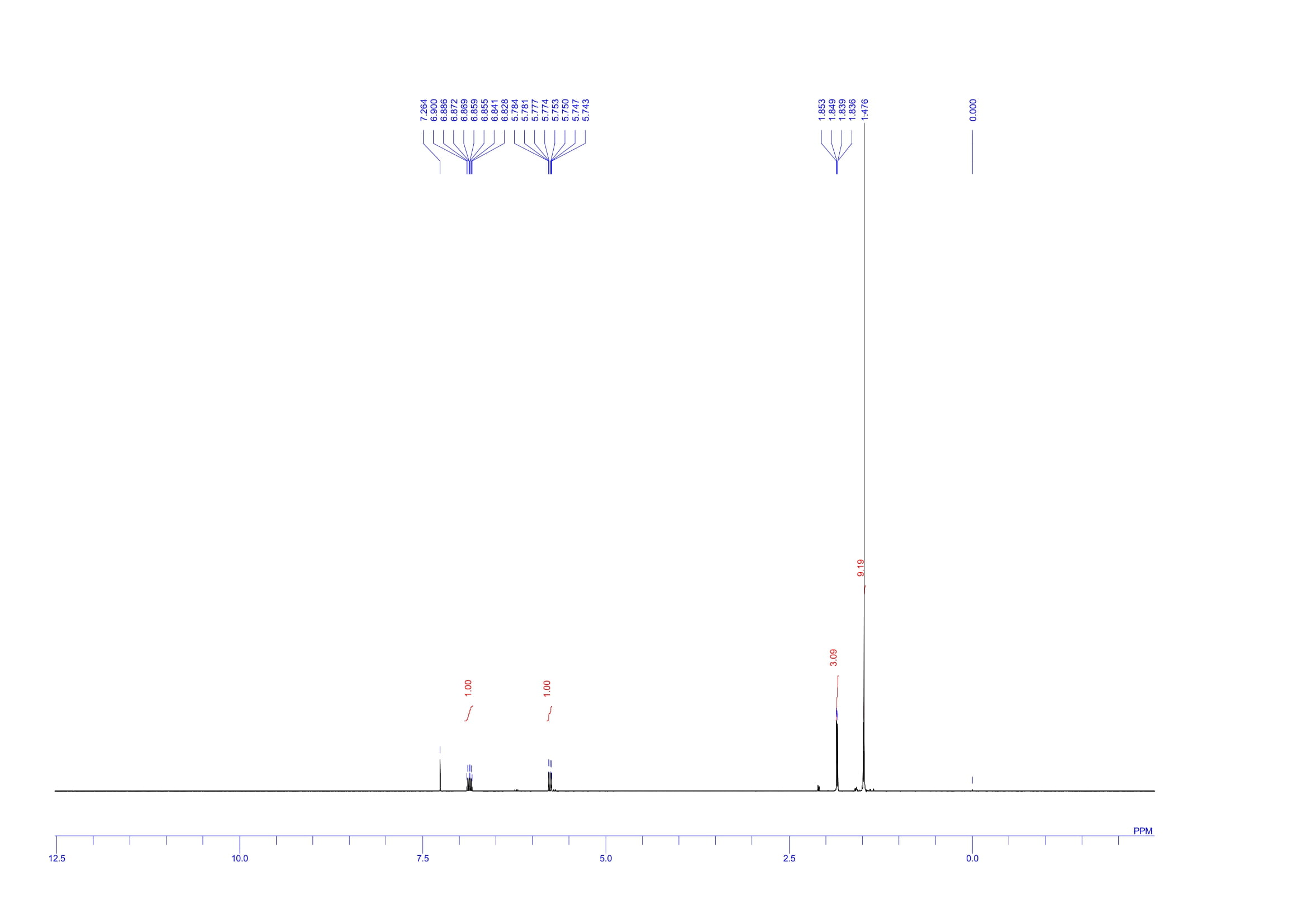
**


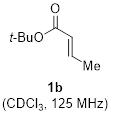
**
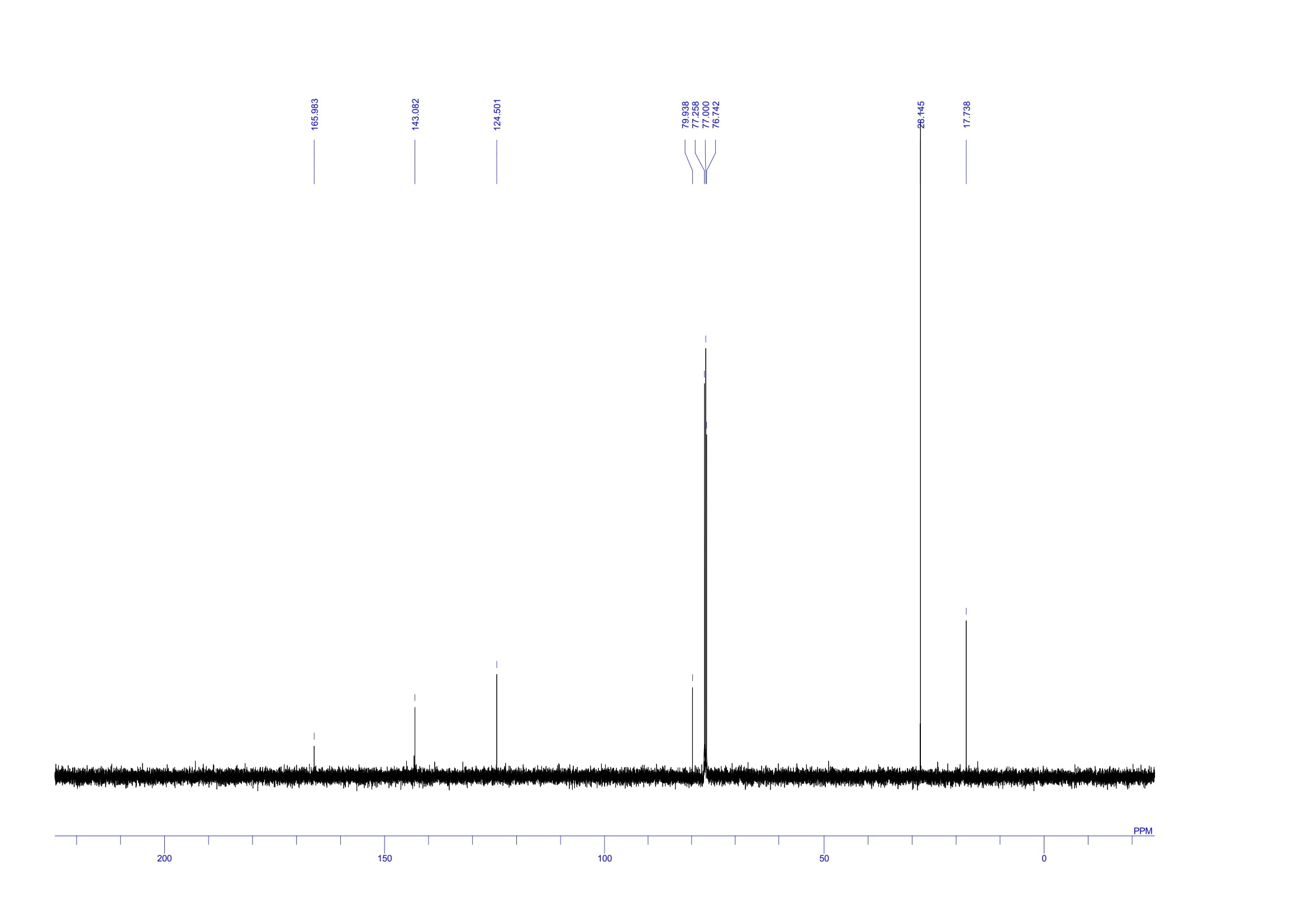
**


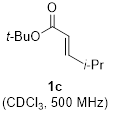
**
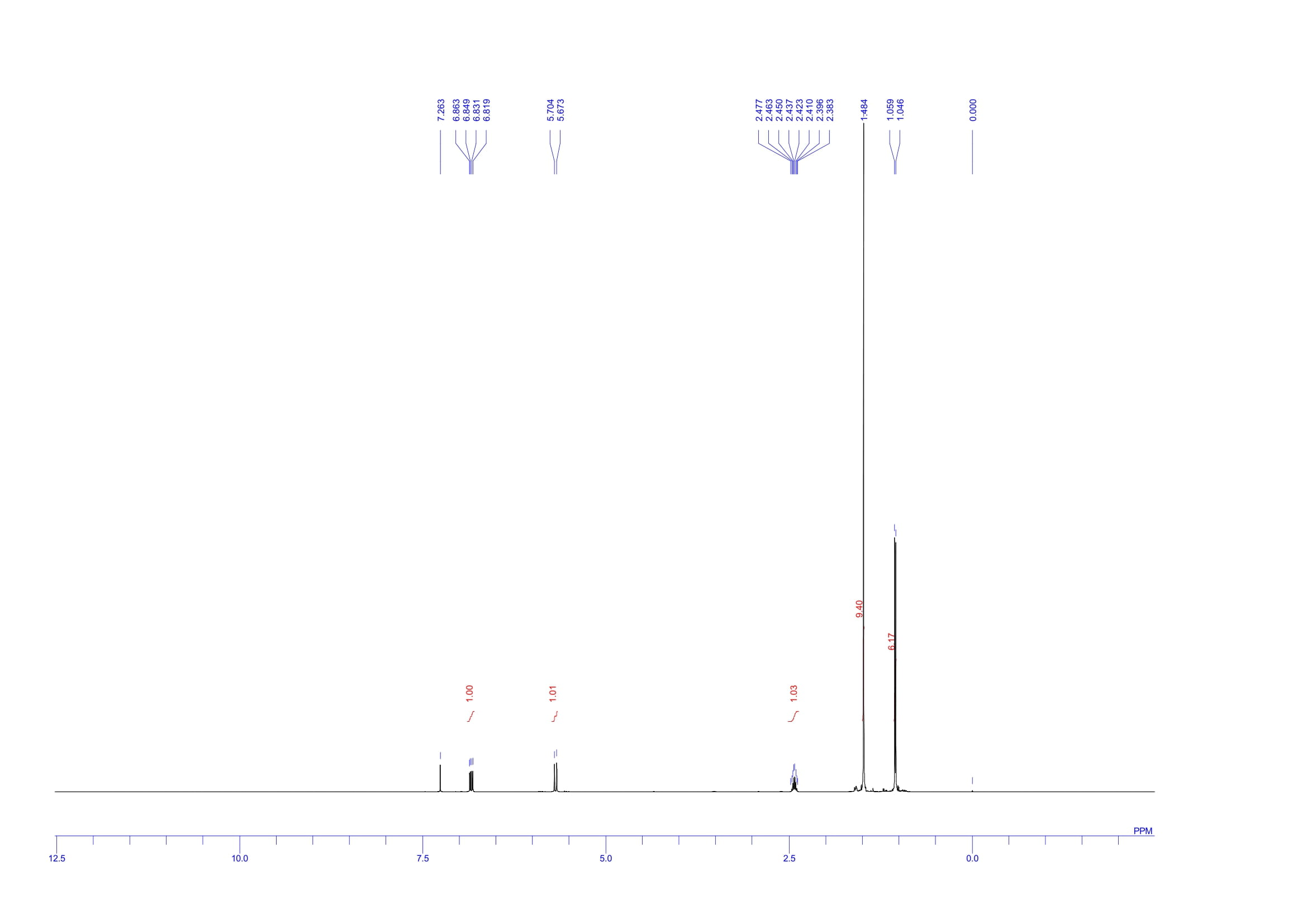
**
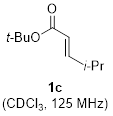
**
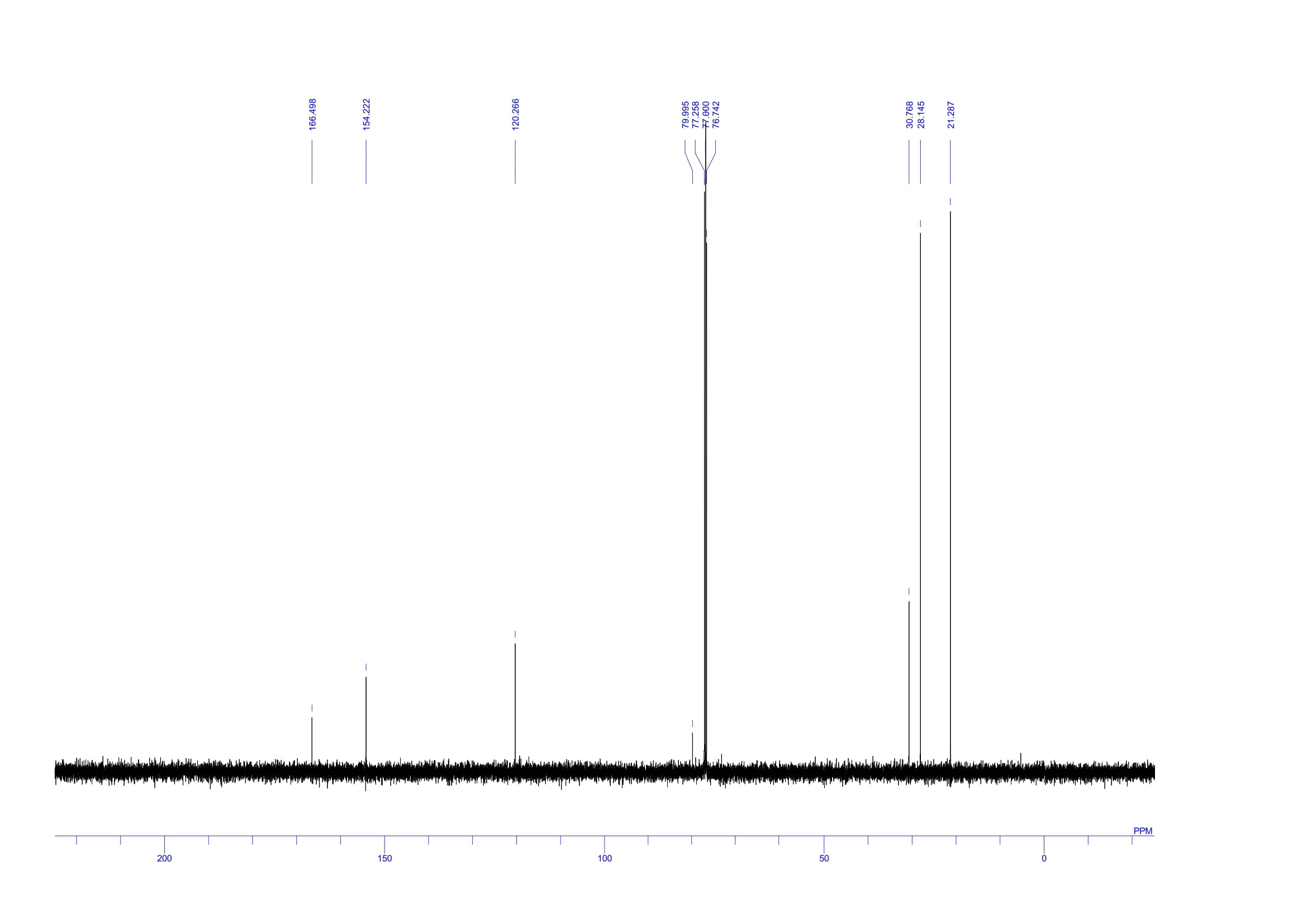
**


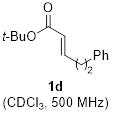
**
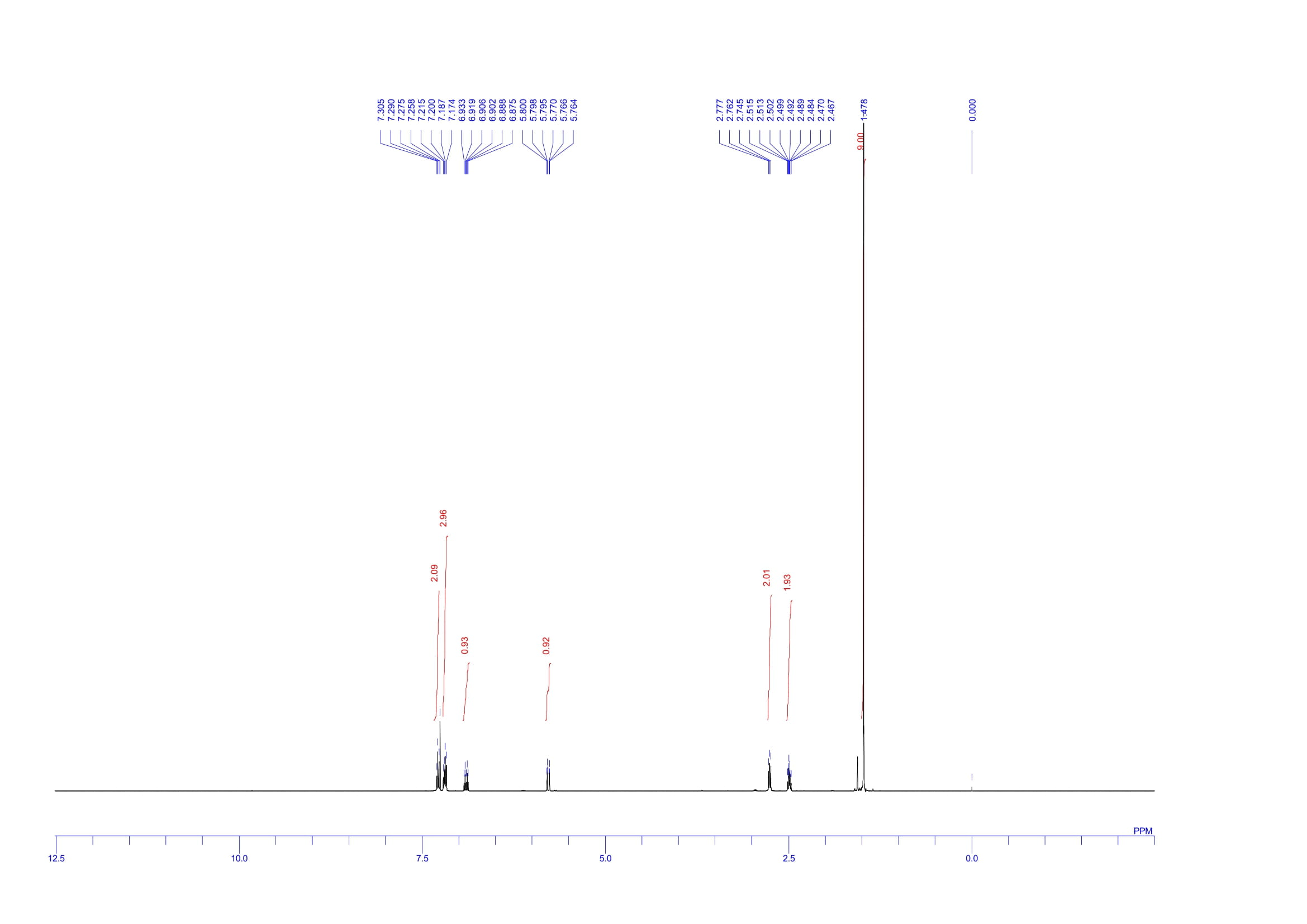
**


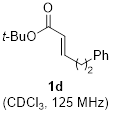
**
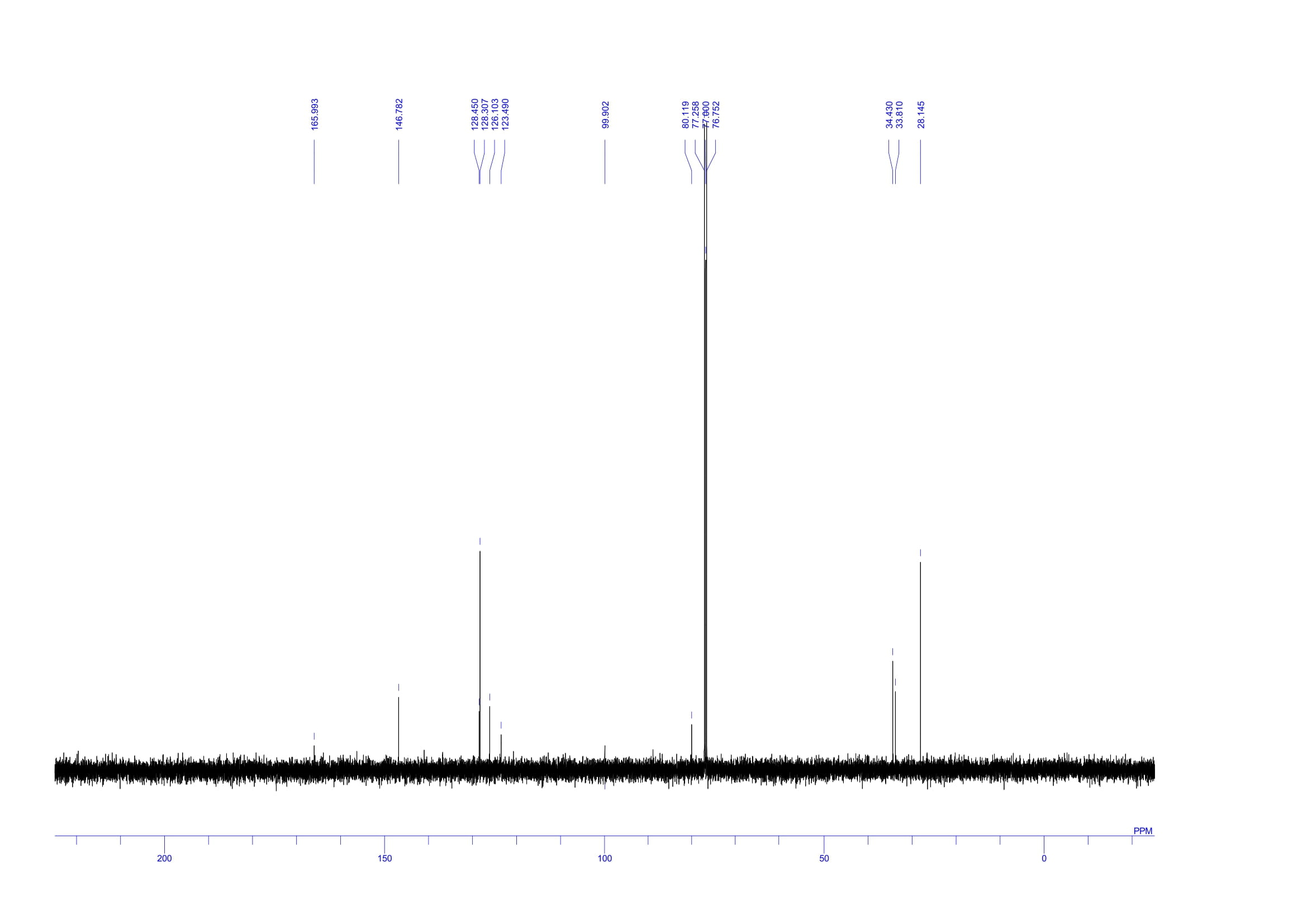
**


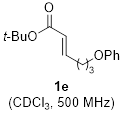
**
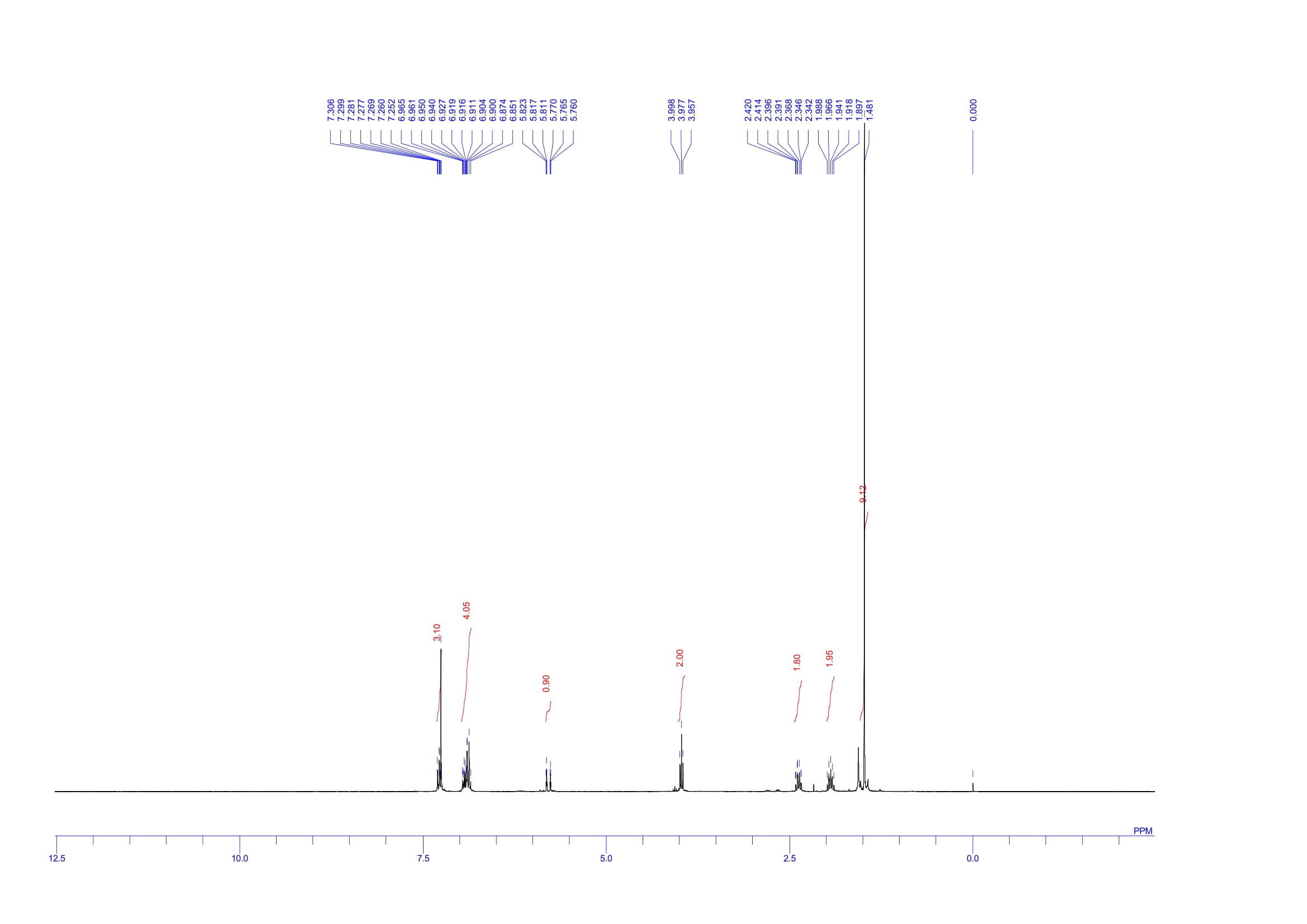
**


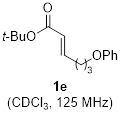
**
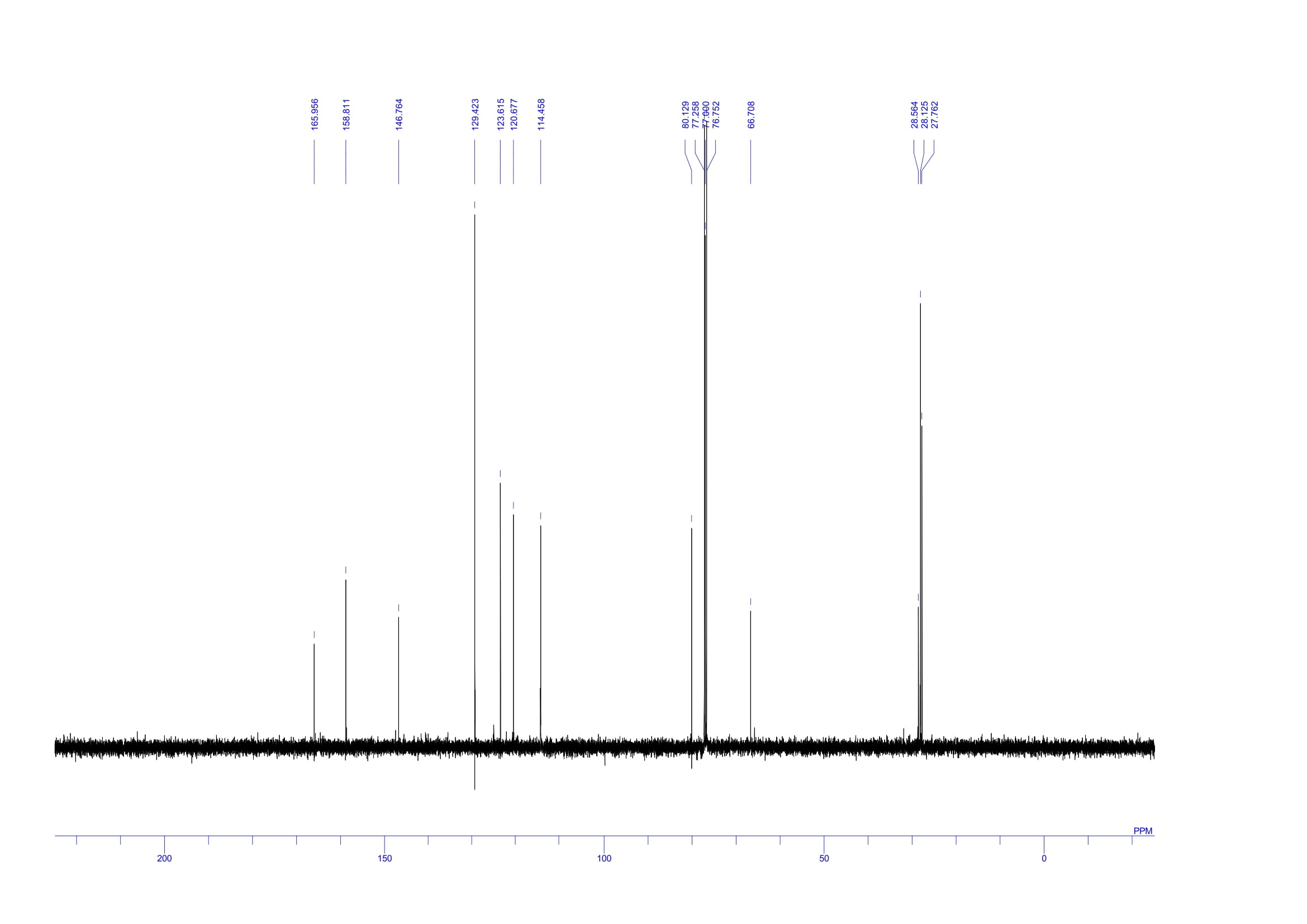
**


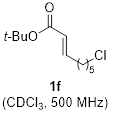
**
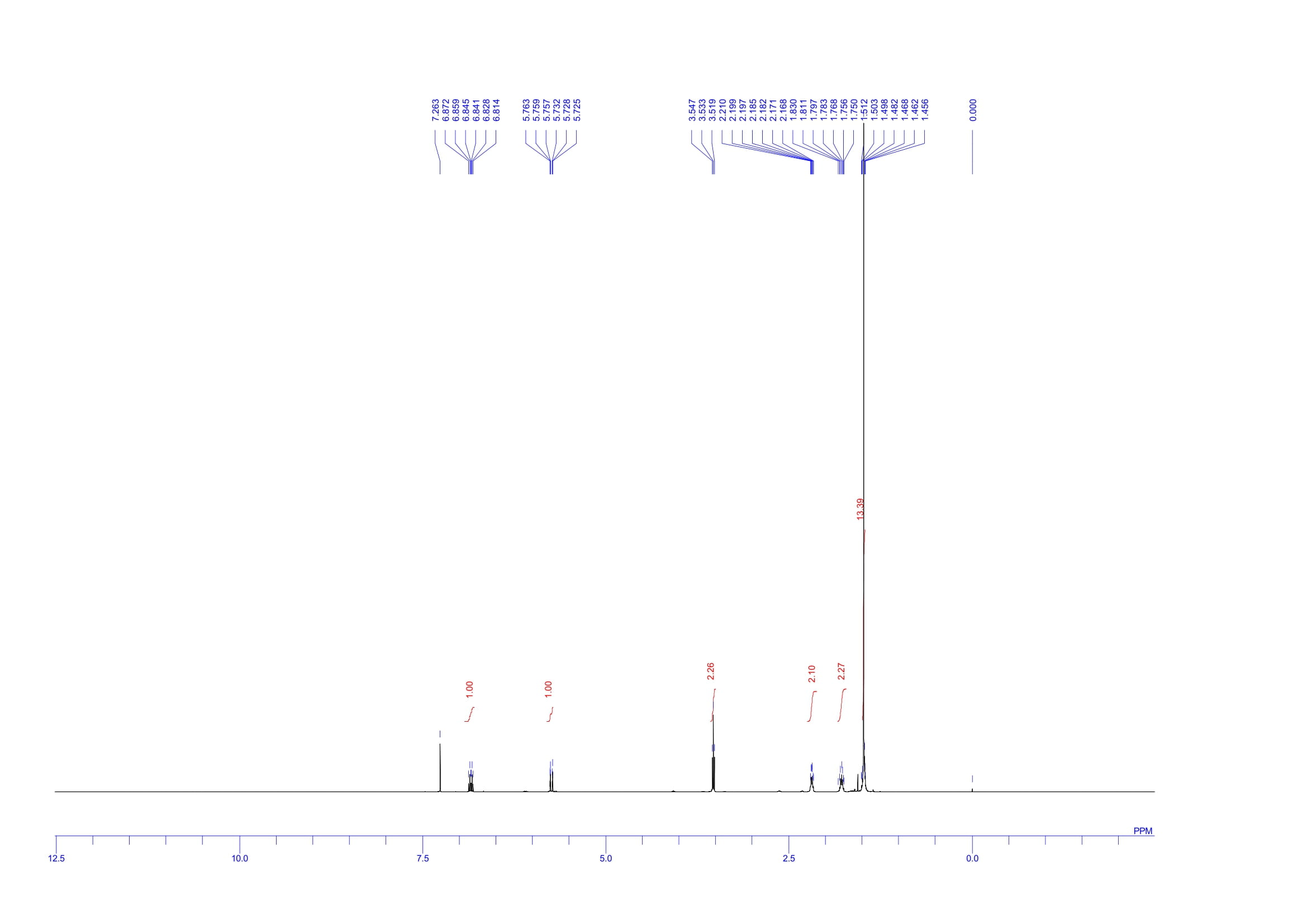
**


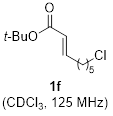
**
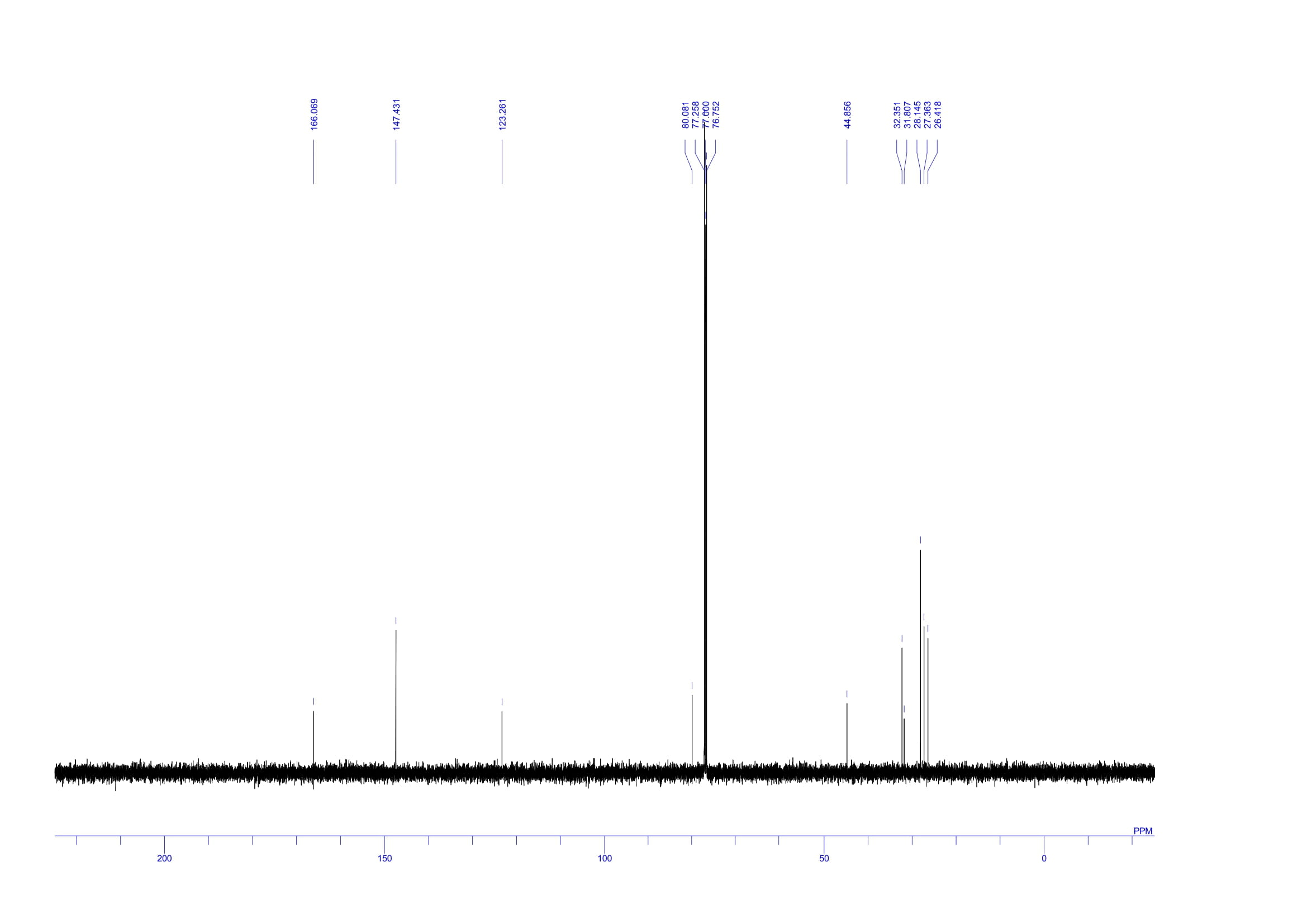
**


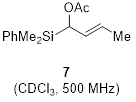
**
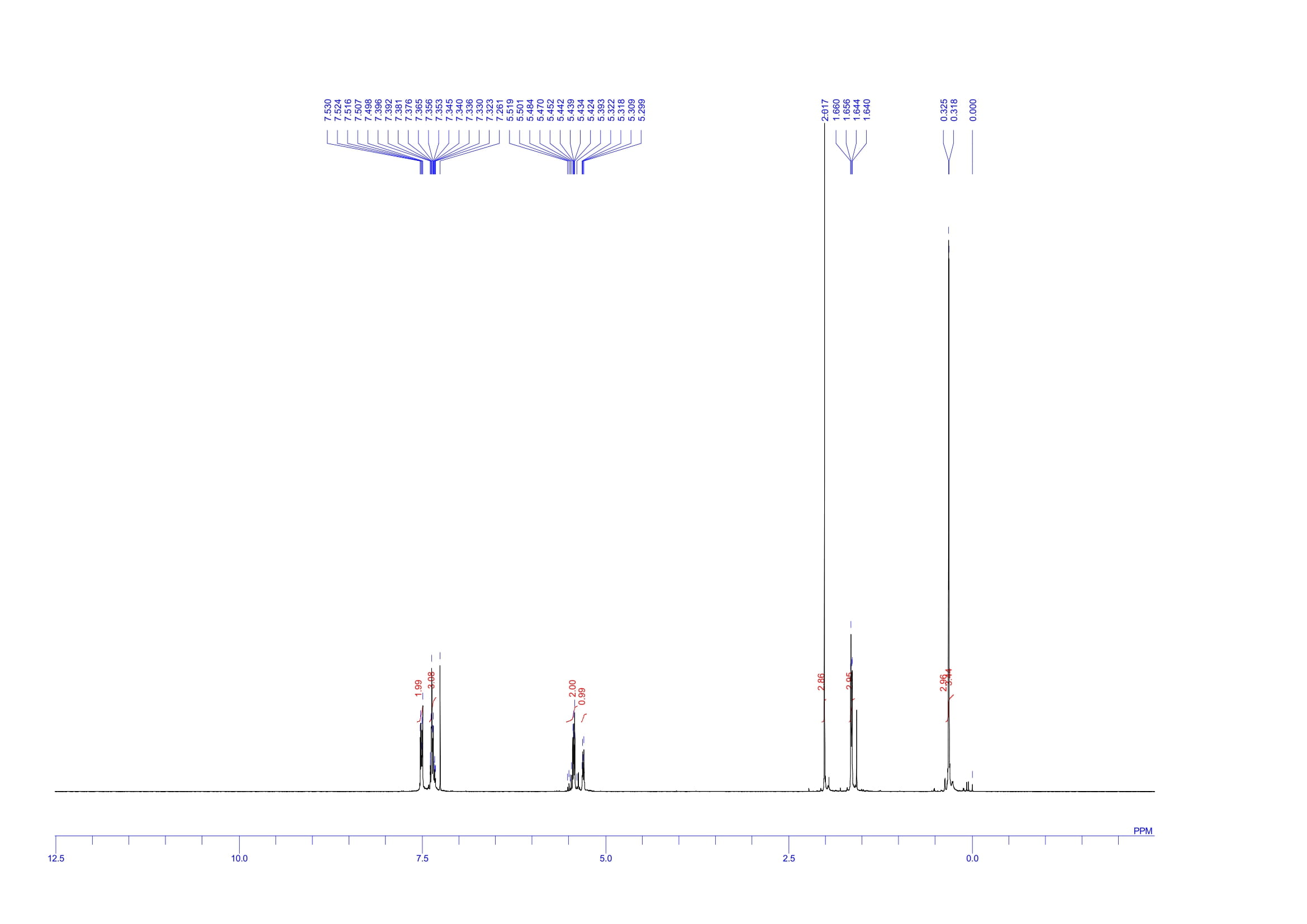
**


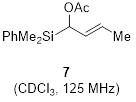
**
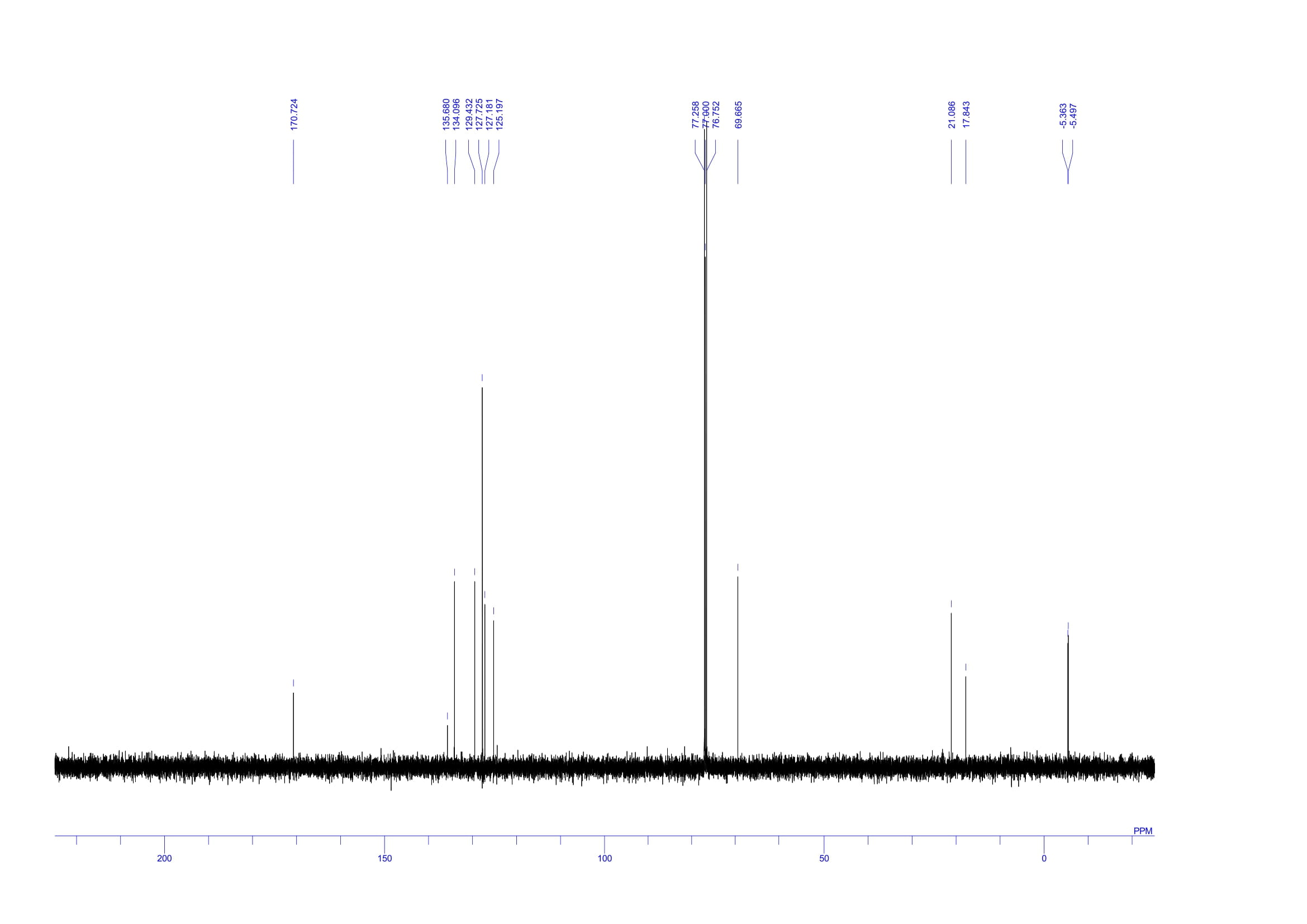
**


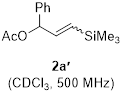
**
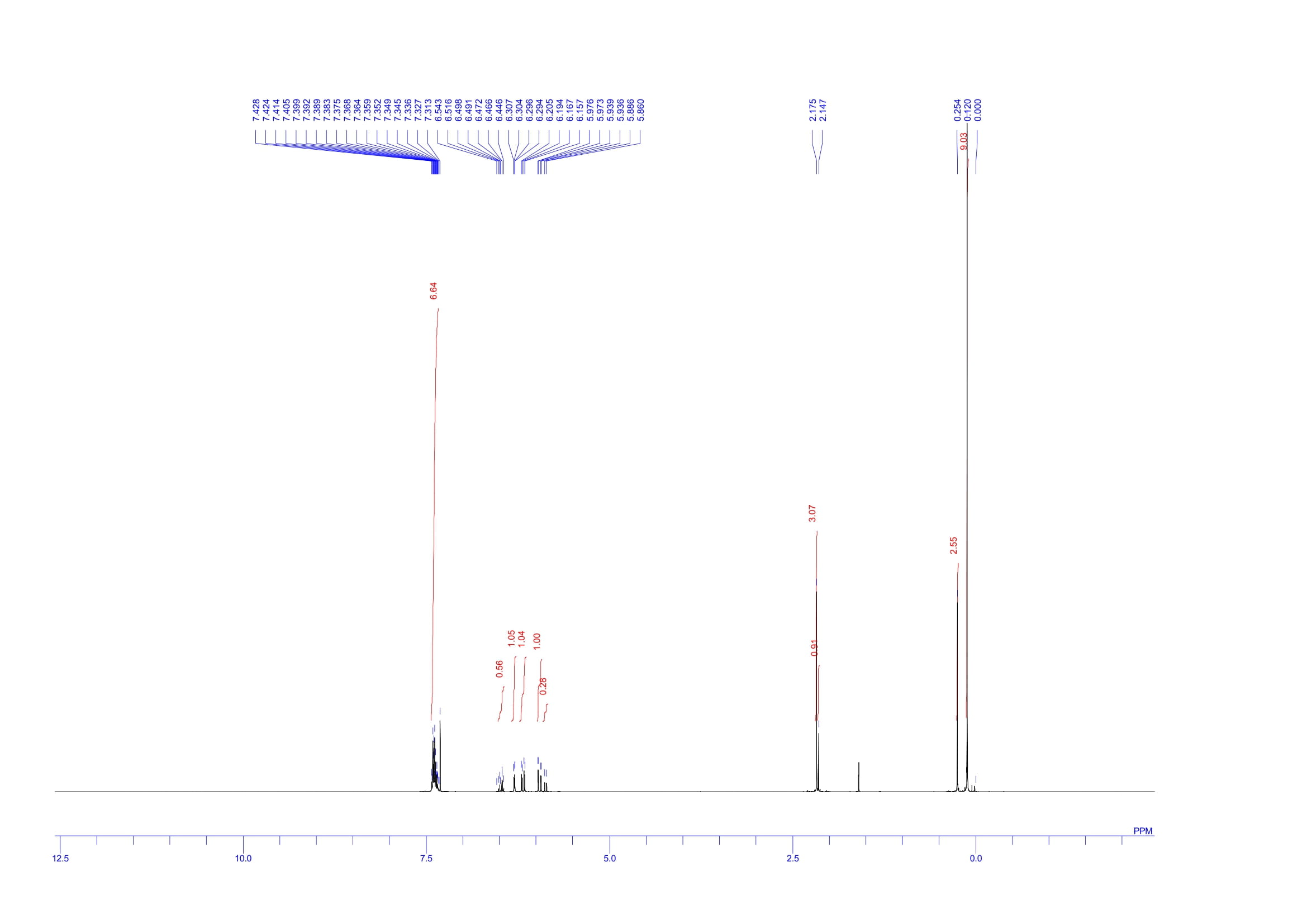
**


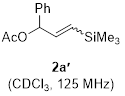
**
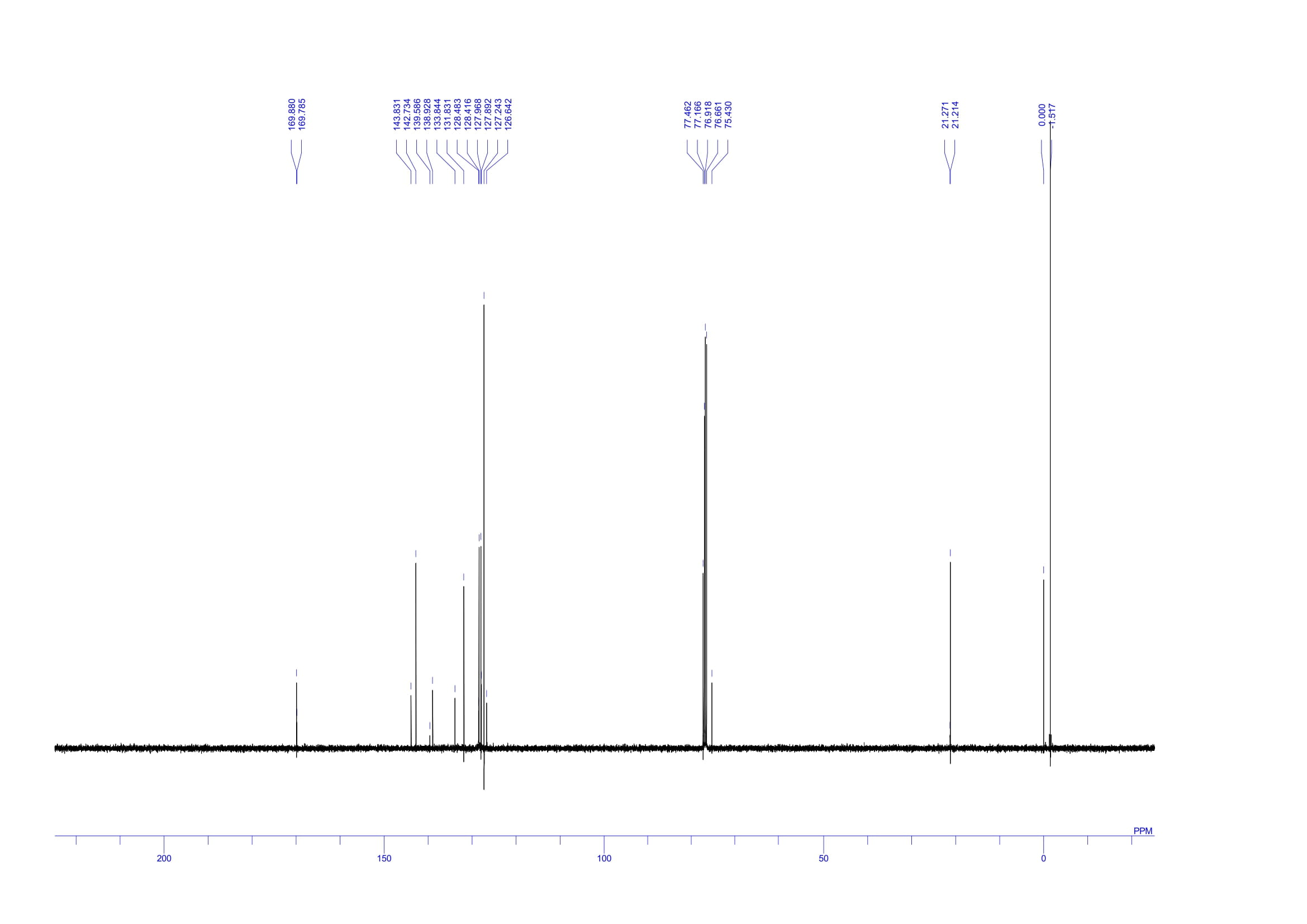
**
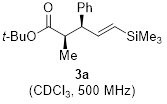
**
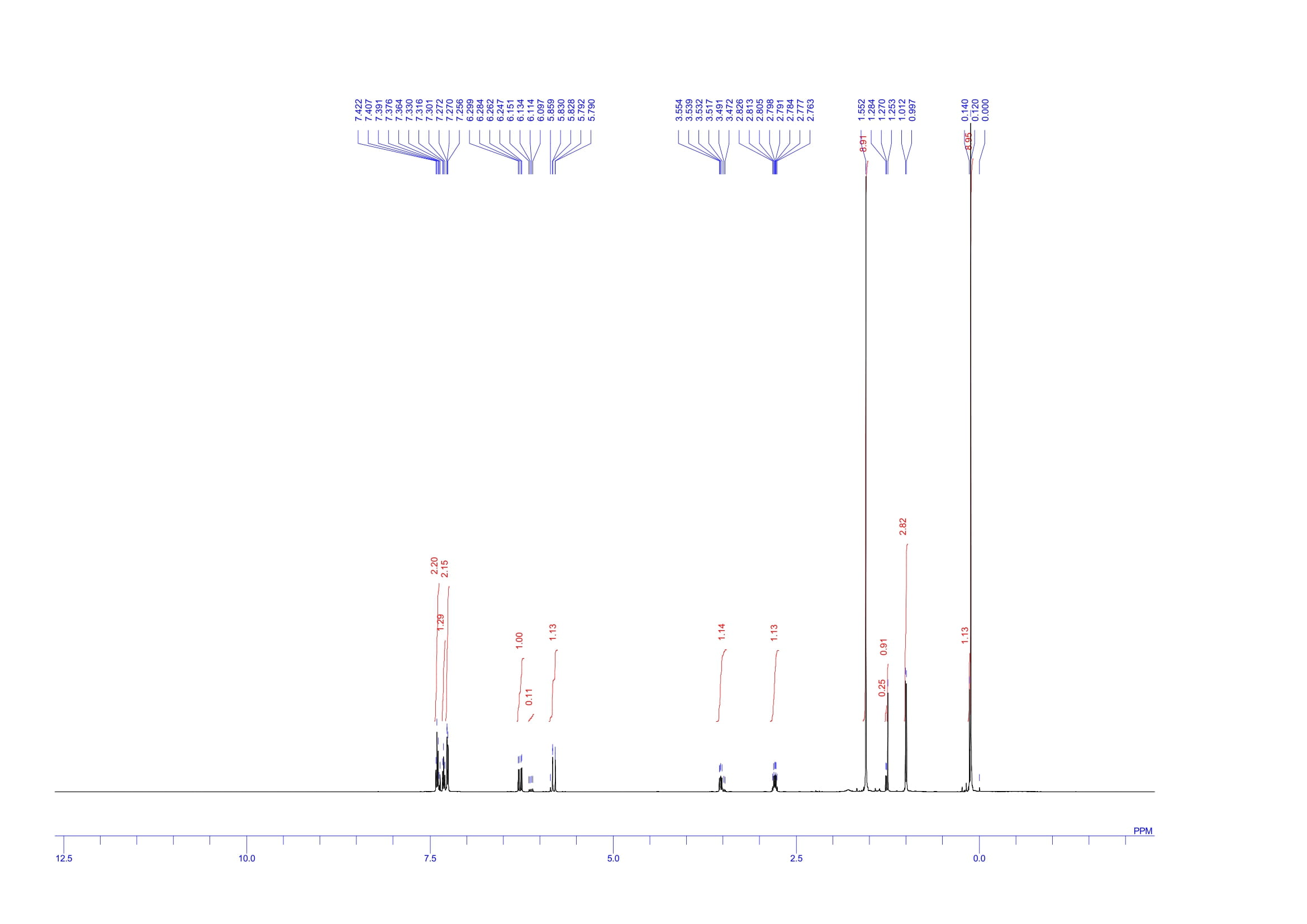
**


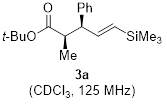


1. **Copies of HPLC Charts for the Products**
